# Supplementary material for: Utilisation and costs of mental health-related service use among adolescents
Source: PLoS One. 2022 Sep 9;17(9):e0273628. doi: 10.1371/journal.pone.0273628 (PMC9462733; doi:10.1371/journal.pone.0273628)

S1 Fig. Flow chart of Brazilian High-Risk Cohort participants included in the mental-health related service use study.

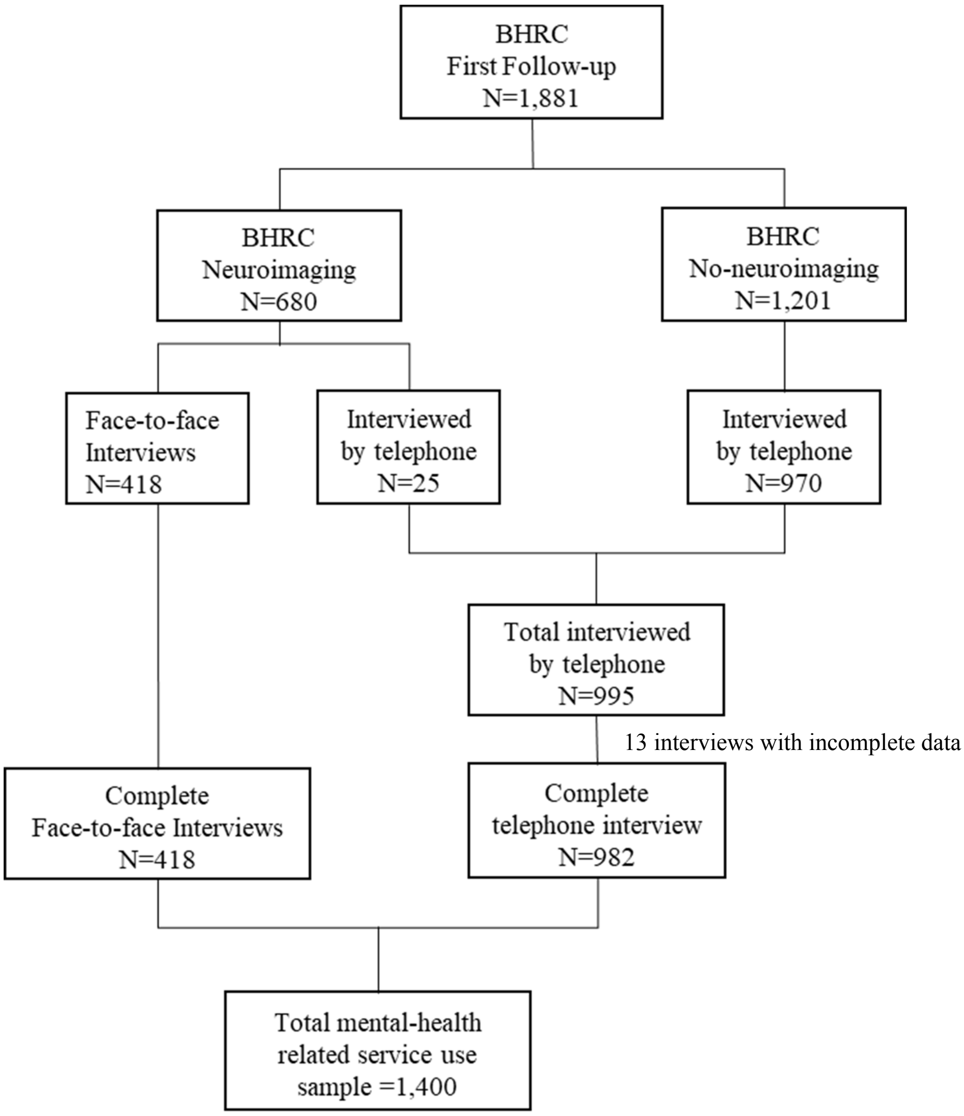

Supplement: S1 Fig — (PDF) [file pone.0273628.s001.pdf]
