# Supplementary material for: Utilisation and costs of mental health-related service use among adolescents
Source: PLoS One. 2022 Sep 9;17(9):e0273628. doi: 10.1371/journal.pone.0273628 (PMC9462733; doi:10.1371/journal.pone.0273628)
Supplement: S1 Table — (PDF) [file pone.0273628.s002.pdf]

S1 Table. Unit costs of health, educational, social care and criminal justice related services.

Public Services	2018 value BR$	2018 value USD$[1]	Notes
Impatient health services			
Psychiatric Hospital, Porto Alegre	257.38 per night	66.42 per night	Based on federal government published data (DATASUS)[2] on average cost per admission to psychiatric hospital of adolescents between 10-19 years, due to any Chapter V ICD-10 diagnosis in Porto Alegre, divided by the average duration of admissions (82.36 BR$ per night). Literature suggests that federal government payment per services is 32% of total costs, when considering state and municipality hospitals funding.[3] DATASUS published figures were adjusted to reflect the total cost (82.36*100/32= 257.38 BR$ per night).
Psychiatric Hospital, São Paulo	132.60 per night	34.22 per night	Estimated from DATASUS[2] 2018 data on average cost per admission to psychiatric hospital of adolescents between 10-19 years, due to any Chapter V ICD-10 diagnosis in São Paulo, divided by the average duration of admissions (42.43 BR$ per night), adjusted to reflect the total cost (42.43*100/32= 132.60 BR$ per night).
Psychiatric bed in General Hospital, Porto Alegre	156.89 per night	40.49 per night	Estimated from DATASUS[2] 2018 data on average cost per admission to general hospital of adolescents between 10-19 years, due to any Chapter V ICD-10 diagnosis in Porto Alegre, divided by the average duration of admissions (50.20 BR$ per night), adjusted to reflect the total cost (50.20*100/32= 156.89 BR$ per night).
Psychiatric bed in General Hospital, São Paulo	182.86 per night	47.19 per night	Estimated from DATASUS[2] 2018 data on average cost per admission to general hospital of adolescents between 10-19 years, due to any Chapter V ICD-10 diagnosis in São Paulo, divided by the average duration of admissions (58.52 BR$ per night), adjusted to reflect the total cost (58.52*100/32 = 182.86 BR$ per night).
Alcohol and Drug clinic, Porto Alegre	123.91 per night	31.98 per night	Estimated from DATASUS[2] 2018 data on average cost per admission to general or psychiatric hospital of adolescents between 10-19 years, related with Mental and behavioral disorders due to psychoactive substance use (F10-F19 codes) ICD-10 in Porto Alegre, divided by the average duration of admissions (39.65 BR$ per night), adjusted to reflect the total cost (39.65*100/32= 123.91 BR$ per night).
Alcohol and Drug clinic, São Paulo	157.34 per night	40.61 per night	Estimated from DATASUS[2] 2018 data on average cost per admission to general or psychiatric hospital of adolescents between 10-19 years, related with Mental and behavioral disorders due to psychoactive substance use (F10-F19 codes) ICD-10 in São Paulo, divided by the average duration of admissions (50.35 BR$ per night),

			adjusted to reflect the total cost (50.35*100/32 = 157.34).
Outpatient health Services	**2018 value BR$**	**2018 value USD$[1]**	**Notes**
Centre for psychosocial care (CAPS)	95.17 per 30 minutes visit	24.56 per 30 minutes visit	Unit cost estimated by considering: a) the average salary for CAPS psychologist and psychiatrists, taken from published data by Razzouk et al.[3] inflated to 2018 values using the Nationwide Consumer Price Index (IPCA)[4] (53.77 BR$ per visit). According to literature staff salaries represent 56.5% of the total costs of one CAPS visit, when considering revenue costs, consumables, non-healthcare human resources, medication costs and capital costs.[5] b) We included: 1) revenue costs (costs of support services –diet service: lunch and snacks-, utilities -expenses with electricity, telephone and gas consumption-; 2) consumables -medical supplies, catering, stationery and cleaning supplies-; 3) non-healthcare human resources -security and one cleaning professional- and overheads -healthcare manager, one assistant and one receptionist-); 4) medication costs; and 5) capital costs (rent, equipment and building maintenance/repair) (53.77*100/56.5=95.17 BR$ per visit).
Any ambulatory mental health clinic	75.42 per 30 minutes visit	19.46 per 30 minutes visit	Unit cost estimated by considering: 1) the average salaries for psychologist and psychiatrists working in ambulatory mental health clinics, taken from published data by Razzouk et al.[3], inflated to 2018 values using the IPCA (42.61 BR$ per visit). 2) We included non-staff costs of one visit[5] (42.61*100/56.5=75.42 BR$ per visit).
Alcohol and drug outpatient clinic	57.12 per 30 minutes visit	14.74 per 30 minutes visit	Unit cost estimated by considering: 1) one psychiatrist visit to an “Alcohol and drugs centre of psychosocial care” (CAPS-AD, based on psychiatrist salary), taken from Becker et al.[5] , inflated to 2018 values using the IPCA (32.27 BR$ per visit), 2) We included non-staff costs of one visit[5] (32.27 *100/56.5= 57.12 BR$ per visit).
Psychologist	43.72 per 40 minutes visit	11.28 per 30 minutes visit	Taken from Razzouk et al.[3], inflated to 2018 values using the IPCA (24.70 BR$ per visit) and adjusted to include non-staff costs of one visit (24.70*100/56.5= 43.72 BR$ per visit).
Psychiatrist	107.09 per 30 minutes visit	27.64 per 30 minutes visit	Taken from Razzouk et al.[3], inflated to 2018 values using the IPCA (60.51 BR$ per visit) and adjusted to include non-staff costs of one visit (60.51*100/56.5= 107.09 BR$ per visit).
General Practitioner - Primary care	67.94 per 30 minutes visit	17.53 per 30 minutes visit	Based on data published by Razzouk et al.[3], inflated to 2018 values using the IPCA (45.45 BR$ per visit). Staff salaries of one visit in a primary care centre would

			represent 66.9% of total costs when considering non-staff costs.[6] We included non-staff costs of one GP visit (45.45*100/66.9= 67.94 BR$ per visit)
Emergency Room	67.17 per 20 minutes visits	17.34 per 20 minutes visits	Cost per 20 minutes visit considering professional and procedures resources, published by Razzouk et al.[3], inflated to 2018 values using the IPCA.
Paediatrician	46.71 per visit	12.05 per visit	Based on federal government published data (DATASUS)[7] on cost of one visit with a paediatrician in 2018 (10.00 BR$), adjusted to reflect total costs when considering state and municipality funding [3] (10.00*100/32 =31.25 BRS$) and we added non-staff costs[6] (31.25*100/66.9= 46.71 BR$ per visit).
Social care and criminal justice services	**2018 value USD$[1]**	**Notes**	**2018 value BR$**
Shelter Porto Alegre	122.47 per night	31.61 per night	Published data in 2017 by the Department of Social care of the municipality of Porto Alegre on monthly cost per user[8], divided by 30 (117.53 BR$ per night), and inflated to 2018 values using the IPCA.
Shelter São Paulo	88.97 per night	22.96 per night	Published data by the Municipal Secretary for Social Assistance and Development of São Paulo in 2011 on monthly cost per shelter user[9], divided by 30 (85.38 BR$ per night), and inflated to 2018 values using the IPCA.
Guardianship council visit	34.00 per home visit	8.77 per home visit	The guardianship council is a five-member committee elected by the population created to ensure the protection of child and adolescents' rights in each municipality. These committees are responsible for visiting and attending children and adolescents with violated rights; meeting with and providing counseling to parents or caretakers; requesting public services related to health, education, social services, welfare, employment, and safety, among other duties. Guardianship councils provide services all day of the year. We calculated the value of one hour from the average national monthly value of the maintenance of the guardianship councils.[10] We considered that each visit can take in average three hours when considered the time of preparation of the visit and travel (1 hour), the home visit (1 hour) and administrative or technical work after home visit (1 hour).
Probation Programme	908.00 per six months participation	234.44 per six months participation	We consulted to the Municipal Secretary for Social Assistance and Development of São Paulo. They did not have information on the cost per user and the average length of probation measures. They provided public tables with the 2018 monthly cost per probation programme service[11] and the number of monthly users per service.[12] We established the average annual cost per user

			(by dividing the average annual cost of services per number of users). As probation measures have a duration of at least six-months, we calculated the cost of this service considering a duration of 6 months of participation (half of annual cost).
Education Services	**2018 value BR$**	**2018 value USD$[1]**	**Notes**
Special School Porto Alegre, Rio Grande do Sul State	4846.21 per school year	1250.70 per school year	2018 annual value per student enrolled in a special school or receiving special education – special class and class assistant – in regular schools by state, published by the Ministry of Education.[13]
Special class Porto Alegre, Rio Grande do Sul State	4846.21 per school year	1250.70 per school year	
Class Assistant Porto Alegre, Rio Grande do Sul State	4846.21 per school year	1250.70 per school year	
Special School São Paulo State	4478.19 per school year	1155.72 per school year	
Special class São Paulo State	4478.19 per school year	1155.72 per school year	
Class Assistant São Paulo State	4478.19 per school year	1155.72 per school year	

General notes: Conversion to US dollars was based on December 31 2018 conversion rate= 0.2581, according the Brazilian Central Bank[1]. Unit costs were based on 2018 prices or the latest available year converted to 2018 prices using the Central Bank calculator to apply the rate according to the Nationwide Consumer Price Index (IPCA)[4].

References

1 Banco Central do Brasil. Conversor de moedas. 2018. https://www.bcb.gov.br/conversao (accessed Oct 10, 2019).

2 Brasil. Ministério da Saúde. Banco de dados do Sistema Único de Saúde-DATASUS. Sistema de Informações Hospitalares do SUS (SIH/SUS)Morbidade Hospitalar do SUS - por local de internação. 2019. http://www2.datasus.gov.br/DATASUS/index.php?area=0203&id=6926&VObj=http://tabnet.datasus.gov.br/cgi/deftohtm.exe?sih/cnv/ni (accessed Sept 13, 2019).

3 Razzouk D. Accommodation and Health Costs of Deinstitutionalized People with Mental Illness Living in Residential Services in Brazil. *PharmacoEconomics - Open* 2019; **3**: 31–42.

4 Banco Central do Brasil. Calculadora do Ciudadão. Correção de valores. 2018. https://www3.bcb.gov.br/CALCIDADAO (accessed Sept 10, 2019).

5 Becker P, Razzouk D. Cost of a community mental health service: a retrospective study on a psychosocial care center for alcohol and drug users in São Paulo. *Sao Paulo Med J* 2018; **136**: 433–41.

6 Curtis L, Burns A. Unit Costs of Health and Social Care 2018. *Univ Kent* 2018.

7 Brasil. Ministério da Saúde. SIGTAP - Sistema de Gerenciamento da Tabela de Procedimentos, Medicamentos e OPM do SUS. 2018. http://sigtap.datasus.gov.br/tabela-unificada/app/sec/procedimento/exibir/0301010072/12/2018.

8 Prefeitura de Porto Alegre. Fundação de Assistência Social e Cidadania. Edital de chamamento publico 0001/2017. Diario oficial Porto Alegre Ed. 5490 p. 54, 2017 http://www2.portoalegre.rs.gov.br/portal_pmpa_novo/default.php?p_noticia=999189701&FASC.

9 Prefeitura de São Paulo. Assistência Social. Planilha-Padrão para Cálculo de Custo dos Serviço.

2011. https://www.prefeitura.sp.gov.br/cidade/secretarias/upload/assistencia_social/arquivos/portarias/PlanilhaCustoServicoAcolhimentoCrAd_final.pdf.

10 Confederação nacional de municípios. A manutenção do Conselho Tutelar nos Municípios. Estudo Técnico. 2017 https://www.cnm.org.br/cms/biblioteca/A manutenção do Conselho Tutelar.pdf.

11 Prefeitura de São Paulo. Secretária Municipal de Assistência e Desenvolvimento Social. Relação de Convênios 2018. 2018. https://www.prefeitura.sp.gov.br/cidade/secretarias/assistencia_social/entidades_sociais/index.php?p=270624 (accessed Sept 17, 2019).

12 Prefeitura de São Paulo. Secretária Municipal de Assistência e Desenvolvimento Social. Indicadores Trimestrais 2018. 2018.

13 Brasil. Ministério da Educação. Portaria Interministerial Nº 6, de 26 de Dezembro de 2018. D. Of. da União. 2018. http://pesquisa.in.gov.br/imprensa/jsp/visualiza/index.jsp?data=27/12/2018&jornal=515&pagina=83&totalArquivos=250.
