# Supplementary material for: Utilisation and costs of mental health-related service use among adolescents
Source: PLoS One. 2022 Sep 9;17(9):e0273628. doi: 10.1371/journal.pone.0273628 (PMC9462733; doi:10.1371/journal.pone.0273628)
Supplement: S2 Table — (PDF) [file pone.0273628.s003.pdf]

S2 Table. Bivariate analysis: Predictors of 12-month mental health service utilisation.

Predictors	Any service use		Health service use		Education service use		Social care and criminal justice service use	
	OR<br>(95%CI)	P	OR<br>(95%CI)	p	OR<br>(95%CI)	p	OR<br>(95%CI)	p
Sociodemographic characteristics								
Female gender	0.82<br>0.57-1.17	0.271	0.90<br>0.62-1.31	0.583	0.47<br>0.18-1.19	0.111	1.34<br>0.53-3.40	0.535
Age	0.99<br>0.91-1.09	0.892	0.97<br>0.88-1.06	0.498	0.97<br>0.79-1.20	0.770	1.22<br>0.96-1.55	0.101
SEG High	(Reference)							
Low	1.16<br>0.81-1.67	0.408	1.00<br>0.67-1.45	0.979	2.39<br>0.88-6.47	0.087	3.32<br>0.96-11.51	0.059
Ethnicity								
White	(Reference)							
Non-White	1.00<br>0.70-1.41	0.981	0.84<br>0.58-1.23	0.371	0.45<br>0.18-1.16	0.098	2.64<br>0.98-7.06	0.054
Guardian characteristics								
Mother's Education								
No education/ basic	(Reference)							
Secondary	1.27<br>0.88-1.85	0.205	1.40<br>0.94-2.01	0.100	1.86<br>0.744.69	0.190	0.45<br>0.15-1.29	0.136
University	1.34<br>0.76-2.39	0.315	1.52<br>0.84-2.78	0.168	1.21<br>0.25-5.88	0.815	0.76<br>0.17-3.48	0.728
Lower parental stigma-RIBS	**1.08**<br>**1.02-1.15**	**0.010**	**1.07**<br>**1.00-1.14**	**0.038**	1.20<br>0.99-1.46	0.060	1.04<br>0.89-1.21	0.619
Clinical characteristics								
Psychiatric diagnosis trajectories								
No diagnosis	(Reference)							
Incident	**4.20**<br>**2.57-6.86**	**<0.001**	**4.49**<br>**2.67-7.55**	**<0.001**	**4.95**<br>**1.23-20.00**	**0.025**	**3.96**<br>**1.05-14.89**	**0.042**
Remittent	**2.76**<br>**1.66-4.58**	**<0.001**	**2.77**<br>**1.61-4.79**	**<0.001**	**6.21**<br>**1.73-22.21**	**0.005**	2.45<br>0.58-10.32	0.223
Persistent	**7.22**<br>**4.50-11.58**	**<0.001**	**7.85**<br>**4.77-12.90**	**<0.001**	**13.77**<br>**4.19-45.33**	**<0.001**	**7.18**<br>**2.16-23.85**	**0.001**
SDQ impact score	**1.49**<br>**1.36-1.62**	**<0.001**	**1.51**<br>**1.38-1.65**	**<0.001**	**1.55**<br>**1.35-1.78**	**<0.001**	**1.31**<br>**1.01-1.57**	**0.003**
Interview method								
In-person	(Reference)							
Telephone	**0.43**<br>**0.30-0.60**	**<0.001**	**0.40**<br>**0.28-0.58**	**<0.001**	0.55<br>0.24-1.26	0.156	0.42<br>0.17-1.07	0.068
