# Supplementary material for: Utilisation and costs of mental health-related service use among adolescents
Source: PLoS One. 2022 Sep 9;17(9):e0273628. doi: 10.1371/journal.pone.0273628 (PMC9462733; doi:10.1371/journal.pone.0273628)
Supplement: S3 Table — (PDF) [file pone.0273628.s004.pdf]

S3 Table. Bivariate analysis: Predictors of cost of mental health service use in the last 12 months

Predictors	Total cost N=133		Health services costs N=115		Education services costs N=22		Social care and criminal justice services costs N=14	
	β (95%CI)	P	β (95%CI)	P	β (95%CI)	p	β (95%CI)	p
Sociodemographic Characteristics								
Female gender	-0.02<br>-0.64-0.59	0.940	0.18<br>-0.47-0.83	0.596	-0.04<br>-0.24-0.16	0.663	1.07<br>-0.46-2.60	0.170
Age	0.01<br>-0.14-0.16	0.899	0.06<br>-0.11-0.22	0.524	0.02<br>-0.063-0.0	0.432	**-0.40<br>-0.73- -0.07**	**0.019**
SEG High	Reference							
Low	0.30<br>-0.29-0.88	0.323	0.18<br>-0.46-0.83	0.577	**-0.18<br>-0.35-<br>-0.003**	**0.047**	3.71<br>-0.62-8.05	0.093
Ethnicity								
White	(Reference)							
Non-White	-0.48<br>-1.07-0.11	0.108	0.03<br>-0.65-0.71	0.927	-0.08<br>-0.27-0.12	0.429	-1.35<br>-2.74-0.04	0.058
Guardian characteristics								
Mother's Education								
No education/basic	(Reference)							
Secondary	0.12<br>-0.55-0.78	0.736	-0.01<br>-0.75-0.74	0.990	-0.03<br>-0.13-0.08	0.643	-0.82<br>-3.32-1.69	0.523
University	0.11<br>-0.91-1.13	0.827	0.03<br>-1.05-1.10	0.960	**0.42<br>-0.24-0.60**	**<0.00<br>1**	-3.85<br>-7.85-0.14	0.059
Lower parental stigma-RIBS	**0.12<br>0.02-0.22**	**0.018**	0.06<br>-0.05-0.17	0.250	-0.01<br>-0.06-0.04	0.697	0.21<br>-0.06-0.48	0.130
Clinical characteristics								
Psychiatric diagnosis trajectories								
No diagnosis	(Reference)							
Incident[3]	0.58<br>-0.17-1.32	0.128	0.54<br>-0.31-1.38	0.212	-0.02<br>-0.28-0.24	0.883	1.23<br>-0.27-2.73	0.109
Remittent	0.68<br>-0.12-1.48	0.096	0.40<br>-0.51-1.32	0.390	0.16<br>-0.08-0.40	0.181	-0.35<br>-1.97-1.27	0.671
Persistent	0.66<br>-0.07-1.38	0.077	0.54<br>-0.26-1.35	0.187	-0.01<br>-0.23-0.22	0.954	**-2.59<br>-4.21-0.97**	**0.002**
SDQ impact score	**0.22<br>0.09-0.35**	**0.001**	**0.18<br>0.06-0.30**	**0.004**	0.01<br>-0.03-0.04	0.747	0.23<br>-0.47-0.93	0.516
Interview method								
In-person	Reference							
Telephone	-0.01<br>-0.60-0.58	0.978	-0.32<br>-0.98-0.35	0.348	0.10<br>-0.07-0.27	0.231	1.27<br>-0.49-3.02	0.156
