# Supplementary material for: Utilisation and costs of mental health-related service use among adolescents
Source: PLoS One. 2022 Sep 9;17(9):e0273628. doi: 10.1371/journal.pone.0273628 (PMC9462733; doi:10.1371/journal.pone.0273628)
Supplement: S4 Table — (PDF) [file pone.0273628.s005.pdf]

S4 Table. Logistic regression models: 12-month mental health service utilisation predicted by psychiatric diagnosis trajectories.

Predictor<br><br>Psychiatric diagnosis trajectories	Any service use		Health service use		Education service use		Social care and criminal justice service use	
	OR 95%CI	P	OR 95%CI	p	OR 95%CI	p	OR 95%CI	p
No diagnosis	Reference							
Incident	**3.73 2.24-6.20**	**<0.001**	**3.91 2.28-6.70**	**<0.001**	**4.70 1.13-19.57**	**0.033**	3.22 0.81-12.83	0.097
Remittent	**2.51 1.49-4.22**	**0.001**	**2.60 1.49-4.56**	**0.001**	**4.73 1.29-17.34**	**0.019**	2.14 0.49-9.36	0.311
Persistent	**6.00 3.67-9.81**	**<0.001**	**6.76 4.04-11.30**	**<0.001**	**9.98 2.85-34.98**	**<0.001**	**5.80 1.66-20.28**	**0.006**
Test statistics								
x^2	88.83		87.98		36.03		24.69	
p value	<0.001		<0.001		0.0002		0.010	
Pseudo R^2	0.10		0.11		0.16		0.13	

Models adjusted by gender, age, SEG, ethnicity, mother's education, city and method of interview.
