# Supplementary material for: Utilisation and costs of mental health-related service use among adolescents
Source: PLoS One. 2022 Sep 9;17(9):e0273628. doi: 10.1371/journal.pone.0273628 (PMC9462733; doi:10.1371/journal.pone.0273628)
Supplement: S5 Table — (PDF) [file pone.0273628.s006.pdf]

S5 Table. Logistic regression models: 12-month mental health service utilisation predicted by impact of behavioural and emotional difficulties on child' life.

Predictor	Any service use		Health service use		Education service use		Social care and criminal justice service use	
	OR (95%CI)	P	OR (95%CI)	p	OR (95%CI)	p	OR (95%CI)	p
**SDQ impact score**	**1.45 1.33-1.58**	**<0.001**	**1.46 1.33-1.60**	**<0.001**	**1.58 1.35-1.86**	**<0.001**	**1.34 1.10-1.64**	**0.004**
Test statistics								
x^2	95.61		93.81		48.32		23.40	
p value	<0.001		<0.001		<0.001		0.005	
Pseudo R^2	0.11		0.11		0.21		0.12	

Models adjusted by gender, age, SEG, ethnicity, mother's education, city and method of interview.
