# Supplementary material for: Utilisation and costs of mental health-related service use among adolescents
Source: PLoS One. 2022 Sep 9;17(9):e0273628. doi: 10.1371/journal.pone.0273628 (PMC9462733; doi:10.1371/journal.pone.0273628)
Supplement: S6 Table — (PDF) [file pone.0273628.s007.pdf]

**S6 Table. Logistic regression models: 12-month mental health service utilisation predicted by parental stigma.**

| Predictor | Any service use | | Health service use | | Education service use | | Social care and criminal justice service use | |
|---|---|---|---|---|---|---|---|---|
| | OR (95%CI) | p | OR (95%CI) | p | OR (95%CI) | p | OR (95%CI) | p |
| **Lower parental stigma-RIBS** | **1.12 1.05-1.19** | **0.001** | **1.10 1.03-1.18** | **0.004** | **1.22 1.00-1.50** | **0.047** | 1.08 0.93-1.26 | 0.303 |
| Test statistics | | | | | | | | |
| $x^2$ | 43.74 | | 38.16 | | 25.90 | | 18.06 | |
| p value | <0.001 | | <0.001 | | 0.002 | | 0.035 | |
| Pseudo $R^2$ | 0.05 | | 0.05 | | 0.12 | | 0.09 | |

Models adjusted by gender, age, SEG, ethnicity, mother's education, city and method of interview
