# Supplementary material for: Utilisation and costs of mental health-related service use among adolescents
Source: PLoS One. 2022 Sep 9;17(9):e0273628. doi: 10.1371/journal.pone.0273628 (PMC9462733; doi:10.1371/journal.pone.0273628)
Supplement: S7 Table — (PDF) [file pone.0273628.s008.pdf]

S7 Table. Generalised linear models: cost of 12-month mental health service utilization predicted by psychiatric diagnosis trajectories.

Predictor<br><br>Psychiatric diagnosis trajectories	Any service costs		Health service costs		Education service costs		Social care and criminal justice service costs	
	β<br>95%CI	p	β<br>95%CI	p	β<br>95%CI	p	β<br>95%CI	p
No diagnosis	Reference							
Incident	0.36<br>-0.45-1.17	0.385	0.54<br>-0.37-1.44	0.245	0.08<br>-1.12-0.28	0.416	**-19.78**<br>**-35.40- -4.16**	**0.013**
Remittent	0.66<br>-0.45-1.33	0.145	0.41<br>-0.58-1.40	0.418	0.0004<br>-0.19-0.19	0.997	**-2.65**<br>**-5.26- -0.04**	**0.047**
Persistent	0.50<br>-0.36-1.36	0.252	0.38<br>-0.49-1.25	0.389	0.17<br>-0.01-0.40	0.141	**-15.13**<br>**-24.64- -5.62**	**0.002**
Test statistics								
AIC	17.21741		16.35117		20.08232		15.64249	
BIC	-327.5553		-301.5434		-30.76184		-2.758111	
R^2	0.09		0.07		0.77		0.86	

Models adjusted by gender, age, SEG, ethnicity, mother's education, city and method of interview.
