# Supplementary material for: Utilisation and costs of mental health-related service use among adolescents
Source: PLoS One. 2022 Sep 9;17(9):e0273628. doi: 10.1371/journal.pone.0273628 (PMC9462733; doi:10.1371/journal.pone.0273628)
Supplement: S8 Table — (PDF) [file pone.0273628.s009.pdf]

S8 Table. Generalised linear models: cost of 12-month mental health service utilisation predicted by impact of behavioural and emotional difficulties on child' life.

Predictor	Any service use		Health service use		Education service use		Social care and criminal justice service use	
	β (95%CI)	p	β (95%CI)	p	β (95%CI)	p	β (95%CI)	p
**SDQ impact score**	**0.24 0.12-0.37**	**<0.001**	**0.17 0.04-0.30**	**0.011**	0.01 -0.01-0.04	0.213	0.26 -0.69-1.21	0.588
Test statistics								
AIC	17.00148		16.2201		19.90144		16.54766	
BIC	-361.593		-322.1063		-36.9233		8.636024	
R^2	0.18		0.12		0.74		0.41	

Models adjusted by gender, age, SEG, ethnicity, mother's education, city and method of interview.
