# Supplementary material for: Utilisation and costs of mental health-related service use among adolescents
Source: PLoS One. 2022 Sep 9;17(9):e0273628. doi: 10.1371/journal.pone.0273628 (PMC9462733; doi:10.1371/journal.pone.0273628)
Supplement: S9 Table — (PDF) [file pone.0273628.s010.pdf]

S9 Table. Generalised linear models: cost of 12-month mental health service utilisation predicted by parental stigma.

Predictor	Any service costs		Health service costs		Education service costs		Social care and criminal justice service costs	
	β (95%CI)	p	β (95%CI)	p	β (95%CI)	p	β (95%CI)	p
**Lower parental stigma-RIBS**	**0.13 0.02-0.24**	**0.019**	0.08 -0.04-0.20	0.207	0.02 -0.03-0.07	0.421	0.35 -0.08-0.79	0.113
Test statistics								
AIC	17.15546		16.31905		19.90198		16.23348	
BIC	-341.422		-310.7278		-36.9114		4.237509	
R^2	0.10		0.07		0.72		0.53	

Models adjusted by gender, age, SEG, ethnicity, mother's education, city and method of interview.
