# Supplementary material for: Utilisation and costs of mental health-related service use among adolescents
Source: PLoS One. 2022 Sep 9;17(9):e0273628. doi: 10.1371/journal.pone.0273628 (PMC9462733; doi:10.1371/journal.pone.0273628)
Supplement: S10 Table — (PDF) [file pone.0273628.s011.pdf]

S10 Table. Generalised linear models: cost of 12-month mental health service utilisation predicted by broad diagnosis categories.

Broad diagnosis categories	β	95%CI			p
**No diagnosis**	Reference				
**Distress and Externalizing**	-0,90	-2,70	-	0,89	0,325
**Fear and Distress**	0,22	-0,77	-	1,21	0,666
**Fear and Externalizing**	0,61	-0,80	-	2,02	0,396
**Full Comorbid**	0,53	-1,27	-	2,32	0,566
**Only Distress**	0,60	-0,43	-	1,63	0,252
**Only Externalizing**	0,17	-0,57	-	0,90	0,658
**Only Fear**	0,62	-0,41	-	1,65	0,235
