# Supplementary material for: Utilisation and costs of mental health-related service use among adolescents
Source: PLoS One. 2022 Sep 9;17(9):e0273628. doi: 10.1371/journal.pone.0273628 (PMC9462733; doi:10.1371/journal.pone.0273628)
Supplement: Attachment — Submitted filename: PONE-D-21-10663_R1_WC.pdf [file pone.0273628.s013.pdf]

PLOS ONE

Utilisation and costs of mental health-related service use among adolescents

--Manuscript Draft--

**Manuscript Number:**	PONE-D-21-10663R1
**Article Type:**	Research Article
**Full Title:**	Utilisation and costs of mental health-related service use among adolescents
**Short Title:**	Adolescent mental health service use and costs
**Corresponding Author:**	Sara Evans-Lacko<br>London School of Economics<br>London, UNITED KINGDOM
**Keywords:**	Service Utilisation, Mental Health, Adolescence, Health Economics
**Abstract:**	Background: The high burden of care for adolescents with mental health disorders represents a challenge to the public sector, especially in low and middle-income countries. We aimed to estimate the costs to the public purse of health, education, criminal justice and social care service use associated with psychiatric disorders among adolescents in Brazil; and to examine whether the trajectory of psychopathology and its impact on daily life, and parental stigma towards mental illness may be associated with service utilisation and costs.<br>Methods: Data on reported service use among adolescents from a prospective community cohort (n=1,400) were combined with Brazilian unit costs. Logistic regression and generalised linear models were used to examine predictors of service use and associated costs, respectively.<br>Results: Twenty-two percent of those who presented with a psychiatric disorder used some type of service for their mental health in the previous twelve months. Higher odds of service use were associated with having a diagnosed mental disorder (either incident, [OR=2.49, 95%CI=1.44-4.30, p=0.001], remittent [OR=2.16, 95%CI=1.27-3.69, p=0.005] or persistent [OR=3.01, 95%CI=1.69-5.36, p<0.001]), higher impact of symptoms on adolescent's life (OR=1.32, 95%CI=1.19-1.47, p<0.001) and lower parental stigma toward mental illness (OR=1.12, 95%CI=1.05-1.20, p=0.001). Average annual cost of service use was 527.14 USD (s.d.= 908.10). Higher cost was predicted by higher disorder impact (b=0.25, 95%CI=0.12-0.39, p<0.001), lower parental stigma (b=0.12, 95%CI=0.02-0.23, p=0.020) and white ethnicity (b=0.55, 95%CI=0.04-1.07, p=0.036). Conclusion: The impact of emotional and behavioural symptoms on adolescents' lives and parental stigmatising attitudes toward mental illness were the main predictors both of service use and costs.
**Order of Authors:**	Carolina Ziebold
	Wagner Silva-Ribeiro
	Derek King
	David McDaid
	Maurício Hoffmann
	Renee Romeo
	Pedro Pan
	Euripides Miguel
	Rodrigo Bressan
	Luis Augusto Rohde
	Giovanni Salum
	Jair Mari
	Sara Evans-Lacko
**Response to Reviewers:**	We appreciate the careful revision of our manuscript and the comments of the

Powered by Editorial Manager® and ProduXion Manager® from Aries Systems Corporation

reviewers. We are pleased to be invited to submit the revised version of our paper to PLOS ONE.

Please find attached both an unmarked version of the revised manuscript and one version with changes marked in red. Our point-by-point responses to the reviewers' comments (unquoted italics) and details of the changes we have performed to our revised manuscript are given below.

Reviewer #1:
General Comment: Very relevant and interesting study. Well written paper, I found it pleasant to read. I would recommend some minor adjustments
Response: We appreciate your positive feedback, the careful revision of our manuscript and your comments.

Comment 1: Abstract- When only reading the abstract, the distinction between incident, remittent and persistent disorder in the Results section is a bit confusing. For the abstract, I would recommend rewriting this sentence for example: "Higher odds of service use were associated with having a diagnosed mental disorder (either incident, remittent or persistent), higher impact of symptoms etc."
Response: Thank you for your comment. We have rewritten this sentence as follows:
Higher odds of service use were associated with having a diagnosed mental disorder (either incident [OR=2.49, 95%CI=1.44-4.30, p=0.001], remittent [OR=2.16, 95%CI=1.27-3.69, p=0.005] or persistent [OR=3.01, 95%CI=1.69-5.36, p<0.001]), higher impact of symptoms..

Comment 2: Introduction- This study focuses on the economic cost of mental disorders in young people (line 52). Therefore, it should be better introduced why, in addition to (mental) health services, also education, criminal justice and social care services were investigated.
Response: We have edited the introduction as follows:
The high prevalence and potentially enduring nature of these impacts make addressing youth mental health conditions particularly important, but this is a challenge for public systems with limited resources (Knapp M; Evans-Lacko S, 2015). Economic costs associated with youth mental health conditions involve a wide range of sectors including health, educational, social care, and criminal justice services [9,10]. This can represent a substantial cost to the public purse, yet it could also be considered a wise investment given the evidence that effective treatment can mitigate the impact of poor mental health (Knapp et al., 2011).

Comment 3: Line 55: male gender is mostly not associated with higher use of mental health services. Please specify the association between these factors and specific services.
Response: We appreciate your suggestion. We have edited this paragraph in the revised version of the manuscript:
Some studies from high–income countries suggest that lower socioeconomic status, as well as clinical features (illness severity and impact of disorders) are associated with use of health, special education, and social care services, while male gender and older age are associated with more criminal justice services contacts [11,13,14]. These sociodemographic and clinical characteristics are also associated with greater mental health-related treatment costs among young people [11,14,15]

Comment 4: Methods: Data and participants. I understand that not all information about the Brazilian High-Risk Cohort was included in this paper. I would want to know, however, based on what information the children became part of this high risk cohort. Are they COPMI?
Response: Thanks for the important point you raised. We have added information in the methods on the Brazilian High-Risk cohort sampling procedures as follows:
This study is nested within the Brazilian High-Risk Cohort (BHRC), which is an ongoing prospective longitudinal study that comprises a community sample and a high-risk sub-sample (a sample at increased risk of mental disorders) of young people from Sao Paulo and Porto Alegre, Brazil. A detailed description of the sample and procedures can be found elsewhere [25]. Briefly, during the registry day, 12,500 parents of young people aged 6 to 14 years attending 57 schools (22 in Porto Alegre and 35 in São Paulo) were invited to a screening of mental health disorders using the Family History

Screen (FHS) [26]. A total of 8,012 families (9,937 eligible children, 45,394 family members) were interviewed. Based on the percentage of members in the family that screened positively for psychiatric disorders, an index of family load for each potential eligible child was computed. The final cohort comprised 2,511 young people; 957 were randomly selected, and 1,554 were a sub-sample at increased risk of mental disorders based on the FHS.

Comment 5: Methods: Measures. Why only maternal educational level?
- Furthermore, this paragraph forms a clear description of appropriate measures.
Response: As stated in the methods section, the socioeconomic group variable comprised head of household educational level in addition to other household socioeconomic indicators. As some research suggests that mothers educational level is particularly important for recognition and help-seeking, we also included this variable as a separate indicator. As the vast majority of caregiver respondents were mothers (in 93% of cases the biological mother [information included in the revised manuscript]) we focused on maternal education rather than estimating the educational level of other caregivers.

Comment 6: Results. Very clear description and informative tables.
Response: Thank you very much for your positive feedback.

Comment 7: Discussion- Line 325: “We found that the health sector was clearly the main sector providing mental health care for youth.” That’s quite obvious. I would recommend rewriting this, for example: “We found that the health sector was clearly the main sector accessed by youth with mental disorders.”
Response: Thank you very much for your suggestion. We rewrote this sentence as follows:
We found that the health sector was clearly the main sector accessed by youth with mental disorders.

Comment 8: In the present study, only 20% of young people with a diagnosed mental disorder received any form of care. In addition to reducing inequality in service use among children, these data also argue for lowering barriers to care for young people in general. I would recommend stating this in the conclusion as well.
Response: Thanks for your suggestion. We have edited the first paragraph of the conclusions as follows:
Our findings suggest that the main drivers of health-related service use costs among adolescents in Brazil were impact of mental health problems, in addition to lower stigma toward people with mental illness among guardians and White ethnicity. In the present study, only 22.4% of young people with a diagnosed mental disorder received any form of care. In addition to reducing inequality in service use among children, our findings also argue for lowering barriers to care, in particular addressing caregiver stigma. Furthermore, because lower use of services in adolescence may be associated with worse outcomes across the life course [47], it is needed to further explore measures to reduce inequalities in service utilisation by young people, even though this implies higher short-term costs.

Comment 9: Line 329: “The lack of youth-oriented primary care mental health programmes”. Is this also the reason why GP’s/family doctors were less frequently visited?
Response: We appreciate your comment, and we agree with your interpretation of this result. We have edited the referred sentence:
The lack of youth-oriented primary care mental health programmes limits access to treatment when symptoms start to have an impact on adolescent functioning. This can explain why we found a low rate of mental health-related contacts with GP/ family doctors. As a result, contact with specialist mental health services only happens when the disorder has significant negative impact on the lives of young people.

Comment 10: Line 359-361: this reads like the impact of mental health problems on children’s lives should be increased because it would support help-seeking. Please, rewrite.
Response: We have rewritten this paragraph:
Guardian’s lower stigmatising attitudes towards mental disorders may be crucial to support young people in accessing, engaging and maintaining contact with mental

Powered by Editorial Manager® and ProduXion Manager® from Aries Systems Corporation

health-related services. Various anti-stigma interventions have demonstrated effectiveness for improving help-seeking [49], but few have been implemented in LMICs. Further studies are needed to design and implement anti-stigma interventions in LMICs. On the other hand, health and education policies need to better support guardians to access appropriate and timely services in their communities, before the symptoms have a significant impact on adolescent functioning.

Comment 11: Line 363: effectives should be effective
Response: Thank you very much, we have corrected this error.

Comment 12: In future research, it would be interesting to not only assess parental stigma but also stigma among the adolescents themselves.
Response: We agree with you, and we are planning to evaluate the association between mental health-related service use and youth stigma towards mental illness in future cohort's assessments.

Reviewer #2:

General comment: It's good to see more representative research from LMICs, trying to bridge the existing knowledge gap. This study's most significant plus point is that it looks at service use and service cost from multiple angles, shedding light on demographic, clinical and systemic factors that contribute to service use cost. However, this manuscript does require significant improvement in language and content. Here are my main suggestions:
Response: We appreciate your positive opinion of our work, the careful revision of our manuscript and your valuable comments.

Comment 1: The language of the manuscript can be crisper. Multiple places sentences look disjointed or elongated. The paragraphs are changed too frequently in some places, with each of these paragraphs containing only one or two sentences.
Response: Thanks for your comment. We have revised and edited the language through the manuscript.

Comment 2: Introduction: In line 57, please clarify whether by 'education services' authors mean remedial education services or some other kind of services?
Response: Thanks for your comment. We have indicated 'special education' in the revised version of the manuscript.

Comment 3: Introduction: The lines 55-58 are difficult to follow: authors claim that certain demographic and clinical characteristics are associated with a greater likelihood of using certain services as per existing research. However, it's not clear how this connects with the assertion about young people in the same sentence.
Response: Thanks for your comment. We have edited and separated these sentences: Some studies from high–income countries suggest that lower socioeconomic status, as well as clinical features (illness severity and impact of disorders) are associated with use of health, special education, and social care services, while male gender and older age are associated with more criminal justice service contacts [11,13,14]. These sociodemographic and clinical characteristics are also associated with greater mental health-related treatment costs among young people [11,14,15].

Comment 4: Introduction: The importance of studying parental stigma needs to be built better.
Response: We appreciate your suggestion. We have included the following changes: Families also play a central role in young people's contact with services. One study from the UK found that lower mental illness-related stigma among caregivers was associated with an increased likelihood of young people's mental health service use [16]. Stigmatising attitudes toward mental illness amongst parents may influence service contacts due to shame and fears of labelling their child's mental health condition [16]. There are clear links between stigma and reduced help-seeking [17], reduced adherence to treatment and early withdrawal from services [17,18]. However, little is known about/ how parental stigma could impact on young people service use and costs.

Comment 5: Introduction: I'm not sure what is meant by 'beyond diagnosis', are authors implying the existing studies cover the cost of diagnosis only or for limited kinds of disorders. Some clarification here would be helpful.
Response: Thanks for your suggestion. We have edited this sentence as follows:
Additionally, little is known about how, in addition to the type of disorder, whether persistence of psychopathology from childhood to adolescence, disorders' impact on adolescent's daily life (i.e., functioning), and key barriers to care such as stigma, could influence costs.

Comment 6: Introduction: The way lines 72-73 are written makes it sound like Brazil is a high-income country
Response: We appreciate your comment. We have deleted 'Similar to most high income countries' in the revised version of the manuscript.

Comment 7: Introduction: In line 88, it's unclear what characteristics the authors are referring to and whether the following hypothesis is related to a subset of these characteristics?
Response: We have rewritten this sentence to clarify the characteristics under study:
Second, we examine how costs vary according to: mental health trajectories, impact of the disorder on everyday life, and parent/guardian stigma towards mental illness.

Comment 8: Methods: In line 96, some information on how these children were classified as high risk will be helpful. The authors have said the details are somewhere else, but a brief description here will make it easier for the reader to understand the sample.
Response: Thanks for your suggestion. As explained in response to Reviewer 1's comment 4, we have included a brief description of the Brazilian High-Risk Cohort sampling procedures.

Comment 9: Methods: In line 99, it was slightly hard to follow study timelines. Was this study carried out after the first follow-up in 2014-2015 or as part of the follow-up?
Response: We have tried to clarify this including the following information:
Cohort participants were interviewed at baseline (aged 6-14 years, calendar year:2010-2011, n=2,511), and at first follow-up (N=2010, aged 9-17 years, calendar year 2014). After completing the BHRC first follow-up interview, 1,881 parents/guardians were invited to respond to a supplementary interview which included a comprehensive assessment of mental health related service use (calendar year: 2014-2015, young people participants aged 10-18 years).

Comment 10: Methods: The authors can use consistent terminology: children or young people. As of now, this has varied from one sentence to another.
Response: Thanks for your comment. We have revised and edited the methods section in order to use consistently the term young people.

Comment 11: The '-' in line 102 seems typo.
Response: We appreciate your comment. We have deleted this typo.

Comment 12: Methods: In lines 127-130, it's unclear why young people were not interviewed at baseline but were included during the 3-year follow-up?
Response: This was because participants were younger at baseline and so we relied on parent's report, given limitations in funding and resources. Given that older adolescents are better at reporting internalising symptoms, both guardian and youth interviews were performed at 3-year follow-up. We included this explanation in the revised version of the manuscript:
At baseline, diagnostic assessment and interviews were performed with guardians only. Previous literature has found that self-reports on internalising conditions during adolescence is higher compared with parental report. This can be explained because internalising problems, such as anxiety or depression, would be less observable by guardians, being advisable to consider both reports to reach a reliable evaluation of adolescent mental health [30,31]. For this reason, diagnostic assessment at 3-year follow-up was performed considering guardian reports and additional information from interviews with the young people about internalising conditions.

Powered by Editorial Manager® and ProduXion Manager® from Aries Systems Corporation

Comment 13: Methods: Do authors have any psychometric properties of the adapted version of Service Assessment for Children and Adolescents that can be reported in this publication?
Response:
The parent-report SACA has been shown to be a valid measure of young people's service use (kappa = 0.76; [Hoagwood et al., 2000]) with test-retest reliability for past-year reports (ranging from 0.75 to 0.86; [Horwitz et al., 2001]). We have not assessed the psychometric properties of the adapted version of the Service Assessment for Children and Adolescents for Brazilian participants yet. We have included this limitation in the revised version of the manuscript.

Comment 14: Results: In line 224, the authors refer to Table 1. However, without any commentary on the significance of data in this table, the authors jump to a new set of findings. All this makes it slightly hard to follow what is being presented.
Response: We appreciated your comment. We have edited this paragraph:
Table 1 describes sociodemographic and clinical characteristics of participants. The sample comprised 1,400 adolescents with a mean age of 14 years (s.d=1.98). The majority were white males from low SEG, and only 10% of mothers had university education. 23.3% (n= 326) of adolescents had a psychiatric disorder in the previous 12 months, of which 177 (54.3%) were incident and 149 (45.7%) persistent cases since baseline. 213 (15.2%) participants had remitted from a baseline psychiatric diagnosis. Participants with externalising disorders were more likely to have persistent trajectories (RR=2.19, 95%CI=1.38-3.48, p<0.001). Participants categorised as persistent also reported greater disorder impact (=2.34, 95%CI=2.11-2.58, p<0.001). 22.4% of those who presented with a psychiatric disorder reported using some type of service for their mental health in the previous twelve months. The proportion of service use among those who presented a persistent psychiatric condition was 27%. Table 1 also describes the mean costs of mental health-related service use in the past year, by psychiatric trajectory (from no diagnosis to persistent psychiatric diagnosis). Bivariate analyses showed a non-significant association between psychiatric trajectory and mean annual costs.

Comment 15: The 12-month service use and service use cost means are presented in Tables 1 and 2. Repeating the same findings across two tables should be avoided
Response: We have deleted the last line of Table 2 (overall services cost).

Comment 16: The paragraph on page 12 lacks a description of the cost associated with each service? For e.g., although CAPS is not a highly prevalent service, the associated cost makes for a lion contribution to the public purse. This needs to be presented and discussed.
Response: Thank you very much for your suggestion. We edited this paragraph as follows:
Utilisation of mental health services in the previous 12 months and associated cost by type of service are presented in Table 2. Overall, 10.0% of the sample (n=143) used some sort of health, education, criminal justice or social care service for mental health problems. Disaggregating by sectors, the health sector had highest proportion of service users (9%), while the education and social care and criminal justice sectors were less frequently contacted with a 1.8% and 1.3% of users, respectively. Outpatient mental health services, most notably psychologists and psychiatrists in settings other than community mental health clinics, were the most frequently used services/professionals. Inpatient services and general health services such as GP/family doctor, paediatrician and emergency department, were less frequently used. In the education sector, school assistant was the most type of service used by young people, while guardianship council was the most frequently social care service contacted. The total cost of 12-month mental health-related service use for the public purse was 70,110.23 USD. The sector that presented higher total annual cost was the health sector, followed by the education and finally the social care and criminal justice sectors. The services that generated the greatest total costs for the heath sector were psychologist (11,339.64 USD) and CAPS (9,628.01 USD). Among those who used services, the average annual cost of service use amounted to 527.14 USD (SD= 908.10 USD, range=8.77- 7,605.58 USD, median=221.10 USD, interquartile range=545.28) per user. Individuals using CAPS (specialty mental health) services (1.1% of the sample) had the highest mean number of visits during the previous year and the highest associated costs among health services. The second highest mean

costs in the health sector were related to hospitalizations in psychiatric hospitals and alcohol and drugs clinics, while the lowest mean costs were attributed to emergency department, paediatrician, outpatient alcohol and drugs and GP/family doctor contacts. Although only 0.1% of individuals used shelters, this type of social service had the highest associated mean cost. Education services were used by 1.8% of individuals and these services had the second highest associated mean costs.

Comment 17: Discussion: In line 288, the use of the terms 'above and beyond' doesn't convey much. To the best of my knowledge, the current analysis nowhere helps to reach this conclusion of above and beyond. I am requesting authors to look at terminology closely.
Response: Thanks for your suggestion. We have removed this language.
We found that impact of mental health problems on daily life and parental stigma were the most consistent and robust drivers of mental health service use and associated costs..

Comment 18: Discussion: Lines 301-312 can be streamlined and better organised.
Response: We have edited the cited lines as follows:
We did not find any study exploring the impact of parental stigmatising attitudes toward mental illness on child treatment costs. Other research has shown that parental stigma can impede problem recognition and help-seeking [17,43]. Higher stigma amongst parents and caregivers may discourage or delay service access for their children [16], which may reduce the short-term public sector direct costs of treatment but be detrimental in the long run. Future research needs to further explore the mechanisms through which parental stigma may be related to service/treatment selection and treatment adherence, in order to explain its impact on treatment costs. Moreover, as lower parental stigma may facilitate earlier service contact, it would be interesting to investigate if lower parental stigma may result in lower costs in the longer term.

Comment 19: Discussion: Line 327: The number of CAPS users was less, but the number of visits and costs for those who used it were very high. These were not reflected in the discussion, nor were its implication for the restructuring health system.
Response: We appreciate your comment. We have edited the discussion as suggested:
In Brazil, access to CAPS does not require any referral. However, the number of CAPS services are limited, and they are focused on treatment of severe mental disorders [24]. The high costs incurred by the mental health sector for the treatment of psychiatric disorders in CAPS may be a result of both, the severity of patients consulting these services and because these services provide intensive outpatient treatments (reflected by the highest number of visits we found), which is costly compared with no-specialized services. It is important to highlight that the lack of youth-oriented primary care mental health services in Brazil which limits access to treatment. This could explain why we found low frequency of mental health-related contacts with GP/ family doctors. As a result, contact with specialist mental health services only happens when the disorder has significant negative impact on the lives of young people. (Moved from the conclusion as suggested in your last comment). In this sense, the organisation of a mental health network of care for adolescents, integrating primary care, social care, education, criminal justice and community youth-specialist services, according to the impact of cases, must be considered in Brazil to adequately plan and allocate scarce public budgets [47].

Comment 20: Discussion: The hypothesis stated that researchers were interested in examining the impact of persistence of psychiatric disorders from childhood to adolescence on service costs; however, the discussion did not give much attention to this part.
Response: Thanks for rising this important comment. We have included the following paragraph:
Contrary to what we expected, we did not find an association between disorder persistence and costs. Our analyses instead found that impact of the disorder on adolescent’s life was the most important clinical predictor and that this was what seemed to drive service use rather than type or persistence of diagnosis. Nevertheless, it is important to consider that we have estimated annual costs, and these do not necessarily reflect the cumulative economic costs of persistent cases across childhood and adolescence.

Powered by Editorial Manager® and ProduXion Manager® from Aries Systems Corporation

	Comment 21: Conclusion: Some of the text in the last paragraph of the conclusion, i.e. those referring to implications, can be moved to discussion and expanded further. I'm not able to comment on cost analysis as this is not my area of expertise.<br>Response: Thanks for your suggestions, we have moved some conclusions to the discussion as explained in response to your Comment #20.
**Additional Information:**	
**Question**	**Response**
**Financial Disclosure**<br><br>Enter a financial disclosure statement that describes the sources of funding for the work included in this submission. Review the submission guidelines for detailed requirements. View published research articles from *PLOS ONE* for specific examples.<br><br>This statement is required for submission and **will appear in the published article** if the submission is accepted. Please make sure it is accurate.<br><br>**Unfunded studies**<br>Enter: *The author(s) received no specific funding for this work.*<br><br>**Funded studies**<br>Enter a statement with the following details:<br>• Initials of the authors who received each award<br>• Grant numbers awarded to each author<br>• The full name of each funder<br>• URL of each funder website<br>• Did the sponsors or funders play any role in the study design, data collection and analysis, decision to publish, or preparation of the manuscript?<br>• **NO** - Include this sentence at the end of your statement: *The funders had no role in study design, data collection and analysis, decision to publish, or preparation of the manuscript.*<br>• **YES** - Specify the role(s) played.<br><br>* typeset	The research presented in this article was funded by the European Research Council under the European Union's Seventh Framework Programme (FP7/2007-2013)/ERC grant agreement no 337673, and supported by the National Institute of Developmental Psychiatry for Children and Adolescents, a science and technology institute funded by Conselho Nacional de Desenvolvimento Cienfifico e Tecnológico (CNPq; National Council for Scientific and Technological Development; grant numbers 573974/2008-0 and 465550/2014-2) and Fundação de Amparo à Pesquisa do Estado de São Paulo (FAPESP grant number 2008/57896-8 and 2014/50917-0). SEL receives support from the UK Medical Research Council in relation to the Mentalkit-Brazil project (MR/R022763/1) and the Economic and Social Research Council. CZ received a doctoral scholarship and research abroad scholarship by the Fundação de Amparo à Pesquisa do Estado de São Paulo (grant number 2018/05586-7 and 2019/08731-0). MSH was supported by the Newton International Fellowship (Ref: NIF\R1\181942), awarded by the Academy of Medical Sciences through the UK Government's Newton Fund Programme. The funding organisations had no role in the study design; collection, analysis or interpretation of data; in the writing of this article; or in the decision to submit the article for publication.
**Competing Interests**<br><br>Use the instructions below to enter a competing interest statement for this submission. On behalf of all authors, disclose any competing interests that	All authors report no conflict of interest associated with this publication. Luis Augusto Rohde has received grant or research support from, served as a consultant to, and served on the speakers' bureau of Aché, Bial, Medice, Novartis/Sandoz, Pfizer/Upjohn, and Shire/Takeda in the last three years. The ADHD and Juvenile Bipolar Disorder Outpatient Programs chaired by Dr Rohde have received unrestricted educational and research support from the following pharmaceutical companies in the last three years:

could be perceived to bias this work—acknowledging all financial support and any other relevant financial or non-financial competing interests.<br><br>This statement is required for submission and **will appear in the published article** if the submission is accepted. Please make sure it is accurate and that any funding sources listed in your Funding Information later in the submission form are also declared in your Financial Disclosure statement.<br><br>View published research articles from *PLOS ONE* for specific examples.<br><br>**NO authors have competing interests**<br><br>Enter: *The authors have declared that no competing interests exist.*<br><br>**Authors with competing interests**<br><br>Enter competing interest details beginning with this statement:<br><br>*I have read the journal's policy and the authors of this manuscript have the following competing interests: [insert competing interests here]*<br><br>* typeset	Novartis/Sandoz and Shire/Takeda. Dr Rohde has received authorship royalties from Oxford Press and ArtMed. None of these commercial relationships alter our adherence to PLOS ONE policies on sharing data and materials.
**Ethics Statement**<br><br>Enter an ethics statement for this submission. This statement is required if the study involved:<br><br>• Human participants<br>• Human specimens or tissue<br>• Vertebrate animals or cephalopods<br>• Vertebrate embryos or tissues<br>• Field research<br><br>Write "N/A" if the submission does not require an ethics statement.	This research was carried out in accordance with the latest version of the Declaration of Helsinki This research was carried out in accordance with the latest version of the Declaration of Helsinki. Parental written informed consent was obtained from all the research subjects. Young people provided verbally informed assent (documented as part of the consent form, and witnessed by the interviewer), and those who were able to read and write also provided written consent. All procedures were approved by the Ethics Committee of the Federal University of São Paulo-UNIFESP (Nº 2.879.533 and - CAAE 06457219.9.0000.5505), Hospital de Clínicas de Porto Alegre (CAAE 06457219.9.3001.5327) and the European Research Commission

Powered by Editorial Manager® and ProduXion Manager® from Aries Systems Corporation

General guidance is provided below. Consult the submission guidelines for detailed instructions. **Make sure that all information entered here is included in the Methods section of the manuscript.**<br><br>**Format for specific study types**<br><br>**Human Subject Research (involving human participants and/or tissue)**<br>• Give the name of the institutional review board or ethics committee that approved the study<br>• Include the approval number and/or a statement indicating approval of this research<br>• Indicate the form of consent obtained (written/oral) or the reason that consent was not obtained (e.g. the data were analyzed anonymously)<br><br>**Animal Research (involving vertebrate animals, embryos or tissues)**<br>• Provide the name of the Institutional Animal Care and Use Committee (IACUC) or other relevant ethics board that reviewed the study protocol, and indicate whether they approved this research or granted a formal waiver of ethical approval<br>• Include an approval number if one was obtained<br>• If the study involved *non-human primates*, add *additional details* about animal welfare and steps taken to ameliorate suffering<br>• If anesthesia, euthanasia, or any kind of animal sacrifice is part of the study, include briefly which substances and/or methods were applied<br><br>**Field Research**<br><br>Include the following details if this study involves the collection of plant, animal, or other materials from a natural setting:<br>• Field permit number<br>• Name of the institution or relevant body that granted permission	
**Data Availability**<br><br>Authors are required to make all data	No - some restrictions will apply

underlying the findings described fully available, without restriction, and from the time of publication. PLOS allows rare exceptions to address legal and ethical concerns. See the PLOS Data Policy and FAQ for detailed information.<br><br>A Data Availability Statement describing where the data can be found is required at submission. Your answers to this question constitute the Data Availability Statement and **will be published in the article**, if accepted.<br><br>**Important:** Stating 'data available on request from the author' is not sufficient. If your data are only available upon request, select 'No' for the first question and explain your exceptional situation in the text box.<br><br>Do the authors confirm that all data underlying the findings described in their manuscript are fully available without restriction?	
**Describe where the data may be found in full sentences. If you are copying our sample text, replace any instances of XXX with the appropriate details.**<br><br>• If the data are **held or will be held in a public repository**, include URLs, accession numbers or DOIs. If this information will only be available after acceptance, indicate this by ticking the box below. For example: *All XXX files are available from the XXX database (accession number(s) XXX, XXX.*).<br>• If the data are all contained **within the manuscript and/or Supporting Information files**, enter the following: *All relevant data are within the manuscript and its Supporting Information files.*<br>• If neither of these applies but you are able to provide **details of access elsewhere**, with or without limitations, please do so. For example:<br><br>*Data cannot be shared publicly because of [XXX]. Data are available from the*	Access to data and Data sharing: CZ have full access to all the data used in the study and takes responsibility for the integrity of the data and the accuracy of the data analysis. Data were provided by the Brazilian High-Risk Cohort study and are available upon request in the Open Science Framework public repository (https://osf.io/ktz5h/).

XXX Institutional Data Access / Ethics Committee (contact via XXX) for researchers who meet the criteria for access to confidential data.<br><br>*The data underlying the results presented in the study are available from (include the name of the third party and contact information or URL).*<br>• This text is appropriate if the data are owned by a third party and authors do not have permission to share the data.<br><br>* typeset	
Additional data availability information:	

Powered by Editorial Manager® and ProduXion Manager® from Aries Systems Corporation

Utilisation and costs of mental health-related service use among adolescents

Carolina Ziebold[1], Wagner Ribeiro[1,2], Derek King[2], David McDaid[2], Mauricio Hoffmann[2,3,4,5], Renee Romeo[6], Pedro Pan[1,5], Euripides Miguel[5,7], Rodrigo Bressan[1,5], Luis Augusto Rohde[5,8,9], Giovanni Salum[5,9], Jair Mari[1,5], and Sara Evans-Lacko[2*]

[1] Departamento de Psiquiatria, Universidade Federal de São Paulo, São Paulo, Brazil

[2] Care Policy and Evaluation Centre, London School of Economics and Political Science, London, United Kingdom

[3] Universidade Federal de Santa Maria, Santa Maria, Brazil

[4] Hospital de Clínicas de Porto Alegre, Porto Alegre, Brazil

[5] National Institute of Developmental Psychiatry for Children and Adolescents, São Paulo, Brazil

[6] King's College London, London, United Kingdom

[7] Universidade de São Paulo, São Paulo, Brazil

[8] ADHD Outpatient Program & Developmental Psychiatry Program, Hospital de Clínicas de Porto Alegre, Brazil.

[9] Universidade Federal do Rio Grande do Sul, Porto Alegre, Brazil

***Corresponding author: Email: S.Evans-Lacko@lse.ac.uk (SEL)**

Abstract

Background: The high burden of care for adolescents with mental health disorders represents a challenge to the public sector, especially in low and middle-income countries. We aimed to estimate the costs to the public purse of health, education, criminal justice and social care service use associated with psychiatric disorders among adolescents in Brazil; and to examine whether the trajectory of psychopathology and its impact on daily life, and parental stigma towards mental illness, may be associated with service utilisation and costs.

Methods: Data on reported service use among adolescents from a prospective community cohort (n=1,400) were combined with Brazilian unit costs. Logistic regression and generalised linear models were used to examine predictors of service use and associated costs, respectively.

Results: Twenty-two percent of those who presented with a psychiatric disorder used some type of service for their mental health in the previous twelve months. Higher odds of service use were associated with having a diagnosed mental disorder (either incident, [OR=2.49, 95%CI=1.44-4.30, p=0.001], remittent [OR=2.16, 95%CI=1.27-3.69, p=0.005] or persistent [OR=3.01, 95%CI=1.69-5.36, p<0.001]), higher impact of symptoms on adolescent's life (OR=1.32, 95%CI=1.19-1.47, p<0.001) and lower parental stigma toward mental illness (OR=1.12, 95%CI=1.05-1.20, p=0.001). Average annual cost of service use was 527.14 USD (s.d.= 908.10). Higher cost was predicted by higher disorder impact (β=0.25, 95%CI=0.12-0.39, p<0.001), lower parental stigma (β=0.12, 95%CI=0.02–0.23, p=0.020) and white ethnicity (β=0.55, 95%CI=0.04–1.07, p=0.036). **Conclusion:** The impact of emotional and behavioural symptoms on adolescents' lives and parental stigmatising attitudes toward mental illness were the main predictors both of service use and costs.

Key words: Service Utilisation, Mental Health, Adolescence, Health Economics

Introduction

Mental health conditions affect 13.4% of children and adolescents globally, representing the leading cause of disability in this age group [1]. They can have long-term impacts on health and social outcomes into adulthood [2–7]. The high prevalence and potentially enduring nature of these impacts make addressing youth mental health conditions particularly important, but this is a challenge for public systems with limited resources [8]. Economic costs associated with youth mental health conditions involve a wide range of sectors including health, educational, social care, and criminal justice services [9,10]. This can represent a substantial cost to the public purse, yet it could also be considered a wise investment given the evidence that effective treatment can mitigate the impact of poor mental health [2]. Estimating the economic cost of mental disorders in young people from the perspective of the public purse and understanding which factors are associated with these costs could support more effective and efficient policy planning and care delivery [8,11,12].

Some studies from high–income countries suggest that lower socioeconomic status, as well as clinical features (illness severity and impact of disorders) are associated with use of health, special education, and social care services, while male gender and older age are associated with more criminal justice services contacts [11,13,14]. These sociodemographic and clinical characteristics are also associated with greater mental health-related treatment costs among young people [11,14,15]. Families also play a central role in young people's contact with services. One study from the UK found that lower mental illness-related stigma among caregivers was associated with an increased likelihood of young people's mental health service use [16]. Stigmatising attitudes toward mental illness amongst parents may influence service contacts due to shame and fears of labelling their child's mental health condition [16]. There are clear links between stigma and reduced help-seeking [17], reduced adherence to treatment

and early withdrawal from services [17,18]. However, little is known about how parental stigma could impact on young people service use and costs.

Most costing studies have focused on a single disorder, commonly autism, attention deficit hyperactivity disorder or conduct disorders [10]. Additionally, little is known about how, in addition to the type of disorder, whether persistence of psychopathology from childhood to adolescence, disorders' impact on adolescent's daily life (i.e. functioning), and key barriers to care such as stigma, could influence costs.

There are a limited number of studies reporting on prevalence of mental health service use in low and middle-income countries (LMICs) [19–21], however, none use validated service use measures. Moreover, prevalence of any use does not capture the intensity of use (e.g. number or type of visits) needed to understand the economic impact of child mental health problems. From a global mental health perspective, examining this issue in a LMIC context, where resources are scarce, is of major significance. Brazil provides universal access to health services and education for the entire population that is free at the point of use, while private health care and education are used by about 20% of the population [22–24]. Estimating the economic cost of mental disorders among young people to the public purse, and understanding which factors are associated with these costs in Brazil is essential for public policy planning, specifically to optimise investment. This approach could also be of value for similar health and welfare systems.

Furthermore, examining the variation in costs according to clinical characteristics of adolescents, beyond type of diagnosis, is important as the impact of psychopathology on daily life and the trajectory of psychopathology from childhood to adolescence, may support service planning and resource allocation in relation to clinical characteristics in a preventive and responsive way.

The aim of this study is to estimate the costs associated with health, education, criminal justice and social care services among a cohort of young people in Brazil. We first present the annual aggregate cost to the public purse and then disaggregate this impact to reflect and understand the relative costs to different sectors. Second, we examine how costs vary according to: mental health trajectories, impact of the disorder on everyday life, and parent/guardian stigma towards mental illness. We hypothesise that persistence of psychiatric disorders from childhood to adolescence and associated impact on adolescents' lives have the greatest influence on costs. However, we also expect that lower levels of parental stigma towards mental illness will predict greater likelihood of service use and hence higher costs.

Methods

Data and participants

This study is nested within the Brazilian High-Risk Cohort (BHRC), which is an ongoing prospective longitudinal study that comprises a community sample and a high-risk sub-sample (a sample at increased risk of mental disorders) of young people from Sao Paulo and Porto Alegre, Brazil. A detailed description of the sample and procedures can be found elsewhere [25]. Briefly, during the registry day, 12,500 parents of young people aged 6 to 14 years attending 57 schools (22 in Porto Alegre and 35 in São Paulo) were invited to a screening of mental health disorders using the Family History Screen (FHS) [26]. A total of 8,012 families (9,937 eligible children, 45,394 family members) were interviewed. Based on the percentage of members in the family that screened positively for psychiatric disorders, an index of family load for each potential eligible child was computed. The final cohort comprised 2,511 young people; 957 were randomly selected, and 1,554 were a sub-sample at increased risk of mental disorders based on the FHS. Cohort participants were interviewed at baseline (aged 6-14 years,

calendar year:2010-2011, n=2,511), and at first follow-up (N=2010, aged 9-17 years, calendar year 2014). After completing the BHRC first follow-up interview, 1,881 parents/guardians were invited to respond to a supplementary interview which included a comprehensive assessment of mental health related service use (calendar year: 2014-2015, young people participants aged 10-18 years). Among those contacted, 1,400 (74.4%) guardians (in 93.1% of cases the biological mother) completed the interview –982 (70.1%) by telephone and 418 (29.9%) face-to-face (See flow chart in S1 Fig.). There were no significant differences in persistence of psychopathology or impact of psychopathology on adolescents' lives among respondents versus non-respondents.

This research was carried out in accordance with the latest version of the Declaration of Helsinki. Parental written informed consent was obtained from all the research subjects. Young people provided verbally informed assent (documented as part of the consent form, and witnessed by the interviewer), and those who were able to read and write also provided written consent. All procedures were approved by the Ethics Committee of the Federal University of São Paulo-UNIFESP (Nº 2.879.533 and CAAE 06457219.9.0000.5505), Hospital de Clínicas de Porto Alegre (CAAE 06457219.9.3001.5327) and the European Research Commission. Data were provided by the Brazilian High-Risk Cohort study and are available upon request in the Open Science Framework public repository (https://osf.io/ktz5h/).

Measures

Sociodemographic Characteristics

Data on the following sociodemographic characteristics were collected: gender, age at follow-up, ethnicity (white and non-white: black, Asian, indigenous or mixed-race), socioeconomic group (SEG), and maternal educational level (no/basic, secondary or university education).

SEG was defined according to a Brazilian standardized questionnaire [27]. Based on families' assets and head of household's education level, a total score ranging for 0 to 46 is given, where greater scores represent higher socioeconomic status. In this study, SEG was categorised as "low" (0-22) and "high" (23-46).

Psychopathology

Psychiatric diagnosis: Psychiatric diagnoses were assessed at baseline and follow-up using the Brazilian-Portuguese version of the Development and Well-being Assessment (DAWBA) [28,29], which is a highly structured interview used to generate DSM-IV diagnoses. Trained interviewers gathered information on current problems causing significant distress or social impairment. At baseline, diagnostic assessment and interviews were performed with guardians only. Previous literature has found that self-report on internalising conditions during adolescence is higher compared with parental report. This can be explained because internalising problems, such as anxiety or depression, would be less observable by guardians, being advisable to consider both reports to reach a reliable evaluation of adolescent mental health [30,31]. For this reason, diagnostic assessment at 3-year follow-up was performed considering guardian reports and additional information from interviews with the young people about internalising conditions. Computerised diagnostic probabilities were then generated based on responses those were carefully evaluated by 9 trained psychiatrists who determined the diagnosis.

Broad psychiatric diagnostic categories: Based on previous literature [32], follow-up DAWBA diagnoses were grouped into three broad categories: distress-related disorders

(including depression, generalised anxiety disorder, obsessive – compulsive disorder, tic, eating disorder), fear-related disorders (including panic, agoraphobia, social anxiety, specific phobia and separation anxiety) and externalising disorders (including conduct disorder, oppositional defiant disorder and attention deficit/hyperactivity disorder).

Persistence of diagnosis: Four categories of diagnostic persistence were created based on presence of diagnosis at baseline and/or follow-up: 1) no diagnosis (no diagnosis at both time points), 2) incident (no diagnosis at baseline and presence of diagnosis at follow-up), 3) remittent (presence of diagnosis at baseline and no diagnosis at follow-up), 4) persistent (presence of diagnosis at both time points).

Impact of mental health problems at follow-up: was measured according to the 'impact supplement' of the Strength and Difficulties Questionnaire (SDQ) which is part of DAWBA. This supplement assesses the impact of behavioural and emotional difficulties on adolescent's lives according to guardian reports. A total score (0-10) was generated by summing 5 items: distress, social impairment in family life, friendships, learning, and leisure activities [33]. Higher scores represent greater impact. The impact score has demonstrated internal consistency, cross-informant correlations, and stability measured across time [33].

Parent-reported stigma towards mental health problems

To assess parental stigma, we applied the Brazilian Portuguese version of the Reported and Intended Behaviour Scale (RIBS-BP) [34,35]. The intended behaviour subscale assesses future intended stigmatising behaviour across four domains: living with, working with, living nearby and continuing a relationship with someone with a mental health problem. Higher scores

represent lower stigma. The RIBS-BP has demonstrated good internal consistency, and good to excellent construct validity [35].

Service use

The Service Assessment for Children and Adolescents (SACA) [36] was used to ask guardians about service contacts made in the past 12 months in response to concerns regarding their child's emotions and behaviour, including alcohol and drugs. The SACA assesses type, nature, frequency and duration of services used, treatments received and settings in which services were delivered. Overall concordance between parent report and records (kappa=0.76) [36] and test-retest reliability for 12-month (kappa=0.75–0.86) service use on the parent version of the SACA is strong [37].

We received permission from the SACA developers to translate and adapt the instrument to the Brazilian context in consultation with experts in the Brazilian mental health system to ensure we covered the relevant service types and settings in Brazil. The list of services and professionals was grouped into three sectors: 1) health care: inpatient services (psychiatric hospital, psychiatric unit in a general hospital, alcohol and drug clinic); outpatient services (Centre for psychosocial care [CAPS], which are the community mental health services in Brazil; mental health clinics; specialist mental health professionals (psychiatrists and psychologists in settings other than CAPS and mental health clinics); general health services and professionals (emergency room, paediatrician, general practitioner [GP] or family doctor); 2) education: special school and special education in regular school (special room and special needs class assistant); 3) social care and criminal justice: overnight stay in a shelter or detention centre; probation programme contact; and home visit of the guardianship council (services responsible for child-rights protection).

Estimation of costs

Data collected on use of services from the BHRC were combined with unit costs to derive service use costs in Brazilian Reals for the financial year 2018 and then converted to US dollars (based on December 31 2018 conversion rate 1 Real=0.2581 dollars, according to the Brazilian Central Bank) [38].

Unit costs: Detailed information on source of information and unit cost values for each service is available in S1 Table. Where available, we applied unit costs previously reported in the Brazilian literature [39,40]. However, as costs of many services have not previously been reported, we performed a thorough consultation process gathering relevant data from public databases of the Ministries of Education and Health, and the social care departments of the municipalities of Porto Alegre and São Paulo (S1 Table).

Unit costs were attached to data on service use frequencies for each type of service (based on the SACA) based on 2018 prices or the latest available year converted to 2018 prices using the Nationwide Consumer Price Index. The Brazil Central Bank's calculator was used to apply the index [41]. Once obtained, information on the unit cost of each service was used to calculate the total annual cost by sector (health, education, social care and criminal justice) for each participant by multiplying the frequency of use (e.g. number of visits, nights) by unit cost.

Data Analysis

Data were analysed using STATA, version 14. First, we described prevalence of socio-demographic and clinical characteristics overall and by persistence of psychopathology. Between-group differences were compared using chi-squared tests. For interval variables, means and standard deviations were calculated and overall significance was tested using one-

way analysis of variance. A significance parameter of $p < .05$ (two-tailed) was applied for all tests.

Unadjusted odds ratios and coefficients for each predictor and covariate in relation to mental health service use and costs are presented in S2 and S3 Tables. To compare the relative impact between our three main predictors (i.e., psychopathological trajectories, impact of the disorder and parental stigma) of service use and costs we also present logistic regression models for each of these variables adjusting for sociodemographic characteristics (gender, age, mother's education, ethnicity and SEG) and dummy variables (mode of data collection and city of residence) (S4-S6 Tables for service use and S7-S9 Tables for costs). We then used multivariable analyses to examine the association between guardian and adolescent characteristics with service use (logistic regression models) and associated costs (generalised linear models – GLM), overall and by sector: 1) health; 2) education; and 3) social care and criminal justice. All multivariable analyses were adjusted by socio-demographic characteristics, mode of data collection and city. For costs GLM, we analysed the subset of participants who used services in the previous 12 months (n=143). Annual costs for each participant were included in the models as a scalar dependent variable, with a Gamma distribution [42], using the log-link function.

Results

Table 1 describes sociodemographic and clinical characteristics of participants. The sample comprised 1,400 adolescents with a mean age of 14 y[illegible]s (s.d=1.98). The majority were white males from low SEG, and only 10% of mothers had university education. 23.3% (n= 326) of adolescents had a psychiatric disorder in the previous 12 months, of which 177 (54.3%) were incident and 149 (45.7%) persistent cases since baseline. 213 (15.2%) participants had remitted from a baseline psychiatric diagnosis. Participants with externalising disorders were more likely

to have persistent trajectories (RR=2.19, 95%CI=1.38-3.48, p<0.001). Participants categorised as persistent also reported greater disorder impact (β=2.34, 95%CI=2.11-2.58, p<0.001). 22.4% of those who presented with a psychiatric disorder reported using some type of service for their mental health in the previous twelve months. The proportion of service use among those who presented a persistent psychiatric condition was 27%[illegible] Table 1 also describes the mean costs of mental health-related service use in the past year, by psychiatric trajectory (from no diagnosis to persistent psychiatric diagnosis). Bivariate analyses showed a non-significant association between psychiatric trajectory and mean annual costs.

Table 1. Sociodemographic and clinical characteristics by trajectories of psychopathology (n=1,400).

	**No psychiatric diagnosis (n=861)**	**Incident psychiatric diagnosis (n=177)**	**Remittent psychiatric diagnosis (n=213)**	**Persistent psychiatric diagnosis (n=149)**	**Overall sample (n=1,400)**	
	N (%)	N (%)	N (%)	N (%)	N (%)	p
Sociodemographic characteristics						
Male gender	503 (58.4)	81 (45.8)	134 (62.9)	83 (55.7)	801 (57.2)	**0.005**
Female gender	358 (41.6)	96 (54.2)	79 (37.1)	66 (44.3)	599 (42.8)	
Age, mean (s.d)	14.50 (2.02)	14.58 (1.90)	14.39 (1.88)	14.67 (1.99)	14.51 (1.98)	0.564
High SEG	359 (41.7)	63 (35.6)	71 (33.3)	61 (40.9)	554 (39.6)	0.095
Low SEG	502 (58.3)	114 (64.4)	142 (66.7)	88 (59.1)	846 (60.4)	
White ethnicity	484 (56.2)	106 (60.2)	116 (54.5)	84 (57.1)	790 (56.6)	0.704
Non-White ethnicity	377 (43.8)	70 (39.8)	97 (45.5)	63 (42.9)	607 (43.5)	
Guardians characteristics						
Maternal no/basic education	387 (45.1)	78 (44.6)	96 (45.3)	59 (39.9)	620 (44.5)	0.953
Maternal secondary education	384 (44.8)	78 (44.6)	93 (43.9)	71 (48.0)	626 (44.9)	
Maternal university education	87 (10.14)	19 (10.9)	23 (10.9)	18 (12.2)	147 (10.6)	
Clinical characteristics						
Any Psychiatric Diagnosis	-	177 (54.3)	-	149 (45.7)	326 (23.3)	**<0.001**
Fear-related	-	92 (52.0)	-	72 (48.3)	164 (11.7)	**<0.001**
Distress-related	-	70 (40.0)	-	60 (40.3)	130 (9.3)	**<0.001**
Externalising	-	49 (27.7)	-	68 (45.6)	117 (8.4)	**0.001**
SDQ impact mean score (s.d)	0.28 (0.73)	1.49 (1.91)	0.78 (1.51)	2.62 (2.41)	0.78 (1.52)	**<0.001**
Mental health-related service use						
12-months service use	43 (5.0)	32 (18.0)	27 (12.7)	41 (27.5)	143 (10.21)	**<0.001**
Mean service use costs USD$ (s.d)	326.41 (395.53)	581.90 (1360.19)	644.35 (795.50)	628.50 (901.02)	527.14 (908.10)	0.400

Notes: Results in bold are significant. SEG, socioeconomic group; SDQ, Strength and Difficulties Questionnaire. 3 missing data in ethnicity variable, 10 missing data in maternal education variable.

Frequency of mental health-related service use and annual service use costs

Utilisation of mental health services in the previous 12 months and associated cost by type of service are presented in Table 2. Overall, 10.0% of the sample (n=143) used some sort of health, education, criminal justice or social care service for mental health problems. Disaggregating by sectors, the health sector had highest proportion of service users (9%), while the education and social care and criminal justice sectors were less frequently contacted with a 1.8% and 1.3% of users respectively[illegible]utpatient mental health services, most notably psychologists and psychiatrists in settings other than community mental health clinics, were the most frequently used services/professionals. Inpatient services and general health services such as GP/family doctor, paediatrician and emergency department, were less frequently used. In the education sector, school assistant was the most type of service used by young people, while guardianship council was the most frequently social care service contacted. The total cost of 12-month mental health-related service use for the public purse was 70,110.23 USD. The sector that presented higher total annual cost was the health sector, followed by the education and finally the social care and criminal justice sectors. The services that generated the greatest total costs for the heath sector were psychologist (11,339.64 USD) and CAPS (9,628.01 USD). Among those who used services, the average annual cost of service use amounted to 527.14 USD (SD= 908.10 USD, range=8.77- 7,605.58 USD, median=221.10 USD, interquartile range=545.28) per use[illegible]dividuals using CAPS (specialty mental health) services (1.1% of the sample) had the highest mean number of visit[illegible]uring the previous year and the highest associated costs among health services. The second highest mean costs in the health sector were related to hospitalizations in psychiatric hospitals and alcohol and drugs clinics, while the lowest mean costs were attributed to emergency department, paediatrician, outpatient alcohol

and drugs and GP/family doctor contacts. Although only 0.1% of individuals used shelters, this type of social service had the highest associated mean cost. Education services were used by 1.8% of individuals and these services had the second highest associated mean costs.

Table 2. 12-month mental health-related service use and costs by type of service (n = 143).

	**Users**	**Number of visits/ nights**	**Number of nights/visits per user**[a]	**Total annual cost per service**	**Annual cost per user**
**Type of service**	n (%)	Total	Mean (Range; s.d.)	USD[a,b]	Mean (Range; s.d.)
Health Sector					
Inpatient mental health services[3]					
Psychiatric hospital	7 (0.5)	73	10.6 (1 – 30;11.87)	4,015.72	573.67 (66.42-1,992.72;691.76)
Psychiatric unit in general hospital	1 (0.1)	1	1 (1)	40.49	40.49
AD clinic	3 (0.2)	48	16 (6 – 27;10.73)	1,767.90	589.30 (191.87-1,096.36;462.10)
Outpatient mental health services					
Centre for psychosocial care (CAPS)	15 (1.1)	452	30.15(1-180;49.38)	9,628.01	740.62 (24.56-4,421.03;1212.73)
Mental Health clinic	17 (1.2)	308	18.13(1-70;19.08)	5,644.64	352.79 (19.46-1,362.50;371.28)
Psychiatrist	33 (2.4)	217	6.56 (1-48;8.95)	5,803.90	181.37(27.64-1,326.60;247.29)
Psychologist	71 (5.1)	1,081	15.23 (1-60;14.97)	11,339.64	171.81(11.28-676.99;168.91)
AD clinic	2 (0.1)	2	1 (1)	14.74	14.74
General Health					
Emergency department	4 (0.3)	9	2.25 (1-4;1.50)	156.0	39.00 (17.34-69.34;26.00)
Paediatrician	3 (0.2)	10	3.33 (2-4;1.16)	120.54	40.18 (24.11-48.22;13.92)
GP/family doctor	5 (0.4)	23	4.60 (2-9;2.97)	403.25	80.65 (35.07-157.80;52.01)
Overall health service use	126 (9.0)			37,679.94	324.83 (11.28-4575.70;590.55)
**Educational sector**[4]					
Special School	7(0.5)		School Year	8,564.92	1,223.56 (1,155.72-1,250.70; 44.53)
Special Class	5 (0.4)		School Year	6,063.55	1,212.71 (1,155.72-1,250.70; 52.02)
School Assistant	12 (0.9)		School Year	14,723.52	1,226.96 (1,155.72-1,250.70; 42.95)
Overall education service use	23 (1.8)			29,351.94	1,276.17 (1,155.72-2,501.40; 270.73)
**Social care and criminal justice sector**					
Shelter	2 (0.1)	210	105 (90-120;21.21)	5,599.95	2,799.98 (2,755.34-2,888.48; 63,12)
Guardianship Council home visit	11 (0.8)	31	2.85 (1-5;1.73)	201.84	25.23 (8.77-43.87;15.15)
Probation programme	8 (0.6)		Six months	1,875.48	234.44
Overall social care and criminal justice related service use	18 (1.3)			4,687.47	334.82 (8.77-2,888.48;1,155.72)

[a]Total cost health sector N=116, Total cost education sector, N=23, Total cost social care and criminal justice sector, N=14. Total cost, N=133. Cases with missing values in 'frequency of visits' were not included in costs estimates: CAPS=2, mental health clinic=1, psychiatrist=1, psychologist=5, AD clinic=1, guardianship council=3.

[b]Costs are expressed U.S. Dollars, 2018 prices. Brazilian Central Bank conversion rate: Brazilian Real=0.2581, December 31st 2018[38]

Characteristics associated with mental health-related service use

Having an incident, remittent or persistent psychiatric disorder, as well as the higher impact of behavioural and emotional difficulties on the adolescents' lives and lower parental stigma, all predicted higher odds of any 12-month service use (Table 3). Service contacts in the health sector were also predicted by the same factors. Service use in the educational sector was predicted by impact, lower parental stigma and low SEG. There were no factors significantly associated with use of social care and criminal justice services.

Table 3. Multivariable logistic regression models: Predictors of 12-month mental health service utilisation (n=1,390[a]).

Predictors	Any service use		Health service use		Education service use		Social care and criminal justice service use	
	OR (95%CI)	p	OR (95%CI)	p	OR (95%CI)	p	OR (95%CI)	p
Sociodemographic characteristics								
Male gender (Ref)	-		-		-		-	
Female gender	0.87 (0.59-1.28)	0.488	0.98 (0.65-1.46)	0.901	0.67 (0.24-.84)	0.435	1.28 (0.48-3.40)	0.627
Age (in years)	1.02 (0.92-1.12)	0.703	1.00 (0.90-1.11)	0.942	0.98 (0.78-1.25)	0.886	1.25 (0.97-1.60)	0.084
High SEG (Ref)								
Low SEG	1.30 (0.86-1.98)	0.211	1.11 (0.72-1.70)	0.646	**4.31 (1.29-14.39)**	**0.018**	2.97 (0.75-11.77)	0.122
White ethnicity (Ref)	-		-		-		-	
Non-White ethnicity	1.17 (0.79-1.72)	0.442	0.97 (0.64-1.46)	0.869	0.53 (0.19-1.48)	0.225	2.59 (0.92-7.28)	0.071
Guardians characteristics								
Maternal no/basic education (Ref)	-		-		-		-	
Maternal secondary education	1.23 (0.82-1.85)	0.315	1.30 (0.84-1.99)	0.238	1.77 (0.66-4.78)	0.257	0.51 (0.17-1.54)	0.233
Maternal university education	1.14 (0.59-2.20)	0.698	1.17 (0.59-2.31)	0.658	1.33 (0.24-7.53)	0.744	1.02 (0.19-5.54)	0.981
Lower parental stigma (RIBS scores)	**1.12 (1.05-1.20)**	**0.001**	**1.11 (1.03-1.18)**	**0.003**	**1.22 (1.01-1.48)**	**0.042**	1.01 (0.94-1.25)	0.251
Clinical characteristics								
No psychiatric diagnosis (Ref)	-		-		-		-	
Incident psychiatric diagnosis	**2.49 (1.44-4.30)**	**0.001**	**2.57 (1.45-4.58)**	**0.001**	2.29 (0.51-10.97)	0.281	2.54 (0.61-10.52)	0.199
Remittent psychiatric diagnosis	**2.16 (1.27-3.69)**	**0.005**	**2.22 (1.25-3.93)**	**0.006**	3.24 (0.84-12.50)	0.087	1.98 (0.45-8.75)	0.369
Persistent psychiatric diagnosis	**3.01 (1.69-5.36)**	**<0.001**	**3.33 (1.82-6.08)**	**<0.001**	2.82 (0.65-12.37)	0.168	3.65 (0.88-15.09)	0.073
SDQ impact score	**1.32 (1.19-1.47)**	**<0.001**	**1.32 (1.19-1.47)**	**<0.001**	**1.51 (1.24-1.84)**	**<0.001**	1.22 (0.97-1.55)	0.096
Test statistics	LR $x^2(13)$= 129.35 p<0.001 Pseudo-R^2=0.14		LR $x^2(13)$= 122.81, p<0.001 Pseudo-R^2=0.15		LR $x^2(13)$= 57.46, p<0.001 Pseudo-R^2=0.25		LR $x^2(13)$= 28.36, p=0.008 Pseudo-R^2 =0.15	

[a]From the total sample, N=1,400, 10 cases had missing data in mother's education and 3 in ethnicity variables. Results in bold are statistically significant (p<0.05). Models adjusted by collection instrument and city.

Characteristics associated with greater mental health related service use costs

When all three sectors were combined into a single total cost variable, greater impact, lower parental stigma and white ethnicity were associated with higher costs (Table 4). Each additional impact score predicted an increase in mean costs of 142.59 USD ($p<0.001$). For parental stigma, each additional RIBS-BP score (indicating lower stigma) increased mean cost by 69.32 USD ($p=0.020$). White ethnicity was associated with having higher mean costs of 295.49 USD ($p=0.036$), compared with non-white participants. No association was found between broad diagnosis categories and costs (S10 Table).

When looking at predictors of costs according to sector, disorder impact was associated with greater health sector service use (predicted mean cost by each impact score= 66.26 USD, $p=0.019$). We did not find any significant association of psychiatric trajectories, impact of disorder or parental stigma with education or social care/criminal justice sectors' costs.

Table 4. Generalised linear models: parental and clinical characteristics associated with cost of mental health service use in the last 12 months, overall and by sector.

**Predictors**	**Any service use N= 131**		**Health service use N=115**		**Education service use N=22**		**Social care and criminal justice service use N=14**	
	β (95%CI)	p	β (95%CI)	p	β (95%CI)	p	β (95%CI)	p
Sociodemographic characteristics								
Male gender (Ref)	-		-		-		-	
Female gender	0.05 (-0.50-0.59)	0.866	0.06 (-0.61-0.73)	0.854	-0.03 (-0.29-0.24)	0.857	14.41 (-5.34-34.17)	0.153
Age (in years)	-0.05 (-0.19-0.10)	0.522	0.06 (-0.14-0.24)	0.572	0.02 (-0.02-0.06)	0.886	-2.11 (-4.87-0.65)	0.133
High SEG (Ref)								
Low SEG	0.47 (-0.08-1.03)	0.092	-0.13 (-0.78-0.53)	0.706	-0.03 (-0.36-0.29)	0.839	3.32 (-8.52-15.15)	0.583
White ethnicity (Ref)	-		-		-		-	
Non-White ethnicity	**-0.55 (-1.07- -0.04)**	**0.036**	-0.12 (-0.75-0.51)	0.707	0.09 (-0.10-0.27)	0.368	-4.28 (-9.80-1.24)	0.129
Guardians characteristics								
Maternal no/basic education (Ref)	-		-		-		-	
Maternal secondary education	0.27 (-0.29-0.82)	0.341	-0.10 (-0.78-0.58)	0.776	-0.07 (-0.23-0.10)	0.418	4.95 (-2.19-12.08)	0.174
Maternal university education	0.003 (-0.90-0.91)	0.995	-0.34 (-1.38-0.69)	0.515	0.40 (-0.07-0.87)	0.094	-	-
Lower parental stigma (RIBS score)	**0.12 (0.12-0.39)**	**0.020**	0.04 (-0.07-0.16)	0.465	0.002 (-0.06-0.06)	0.948	0.05 (-0.98-1.08)	0.922
Clinical characteristics								
No psychiatric diagnosis (Ref)	-		-		-		-	
Incident psychiatric diagnosis	-0.14 (-0.83-0.55)	0.693	0.15 (-0.71-1.00)	0.735	0.07 (-0.15-0.29)	0.548	-23.61 (-54.48-7.27)	0.134
Remittent psychiatric diagnosis	0.39 (-0.35-1.14)	0.298	0.09 (-0.85-1.04)	0.847	-0.01 (-0.23-0.21)	0.928	-2.17 (-7.42-3.07)	0.417
Persistent psychiatric diagnosis	-0.39 (-1.16-0.38)	0.315	-0.42 (-1.40- 0.58)	0.412	0.14 (-0.11-0.39)	0.276	-17.23 (-36.84-2.39)	0.085
SDQ impact score	**0.25 (0.12-0.39)**	**<0.001**	**0.20 (1.19-1.47)**	**0.019**	0.01 (-0.02-0.04)	0.458	-0.34 (-1.51-0.83)	0.569
Test statistics[a]	AIC 16.97193<br>BIC -353.9633<br>$R^2 = 0.22$		AIC 16.24671<br>BIC -308.0671<br>$R^2 = 0.15$		AIC 20.26365<br>BIC -24.59063<br>$R^2 = 0.79$		AIC 15.83608<br>BIC 1.230216<br>$R^2 = 0.90$	

Notes: Results in bold are significant ($p<0.05$). Models adjusted by city and method of interview.

[a]Cameron & Windmeijer's *R*-squared, measure of goodness of fit for the class of exponential family regression models.

Discussion

We analysed data on mental health-related service use and associated costs among a prospective community cohort of young people in Brazil. We found that impact of mental health problems on daily life and parental stigma were the most consistent and robust drivers of mental health service use and associated costs.

Drivers of mental-health service use costs

The association between disorder impact and mental health-related service use and costs that we found has been observed in previous research, providing further support that impact and impairment tend to be the strongest and most robust predictors of mental health service use [13,33] and costs [14]. Contrary to what we expected, we did not find an association between disorder persistence and costs. Our analyses instead found an that impact of the disorder on adolescent’s life was the most important clinical predictor and that this was what seemed to drive service use rather than type or persistence of diagnosis. Nevertheless, it is important to consider that we have estimated annual costs, and these do not necessarily reflect the cumulative economic costs of persistent cases across childhood and adolescence.

A novel result we found was that lower parental stigma was associated with greater service use and higher costs. Our findings suggest that the ways in which parents perceive mental illness in adolescents may significantly influence help-seeking. We are aware of one study which showed that young people’s likelihood of service use across health and education settings was greater among caregivers who reported less intended stigmatising behaviours [16] Another study indicated that low parental stigmatising attitudes toward mental disorders increased recognition of mental health problems in preadolescents (10-12 years) [43].

We did not find any study exploring the impact of parental stigmatising attitudes toward mental illness on child treatment costs. Other research has shown that parental stigma can

impede problem recognition and help-seeking [17,43]. Higher stigma amongst parents and caregivers may discourage or delay service access for their children [16], which may reduce the short-term public sector direct costs of treatment but be detrimental in the long run. Future research needs to further explore the mechanisms through which parental stigma may be related to service/treatment selection and treatment adherence, in order to explain its impact on treatment costs. Moreover, as lower parental stigma may facilitate earlier service contact, it would be interesting to investigate if lower parental stigma may result in lower costs in the longer term.

Among sociodemographic variables, we found that low SEG predicted higher odds of educational service use. This may be related to the fact that young people living in deprived circumstances are more likely to be affected by developmental problems [44], and, therefore, are more likely to use special education services [11]. Although our study did not identify any differences in service use according to ethnicity, we found white ethnicity was associated with higher service use costs. This may reflect disparities in the type of mental health treatment offered or available to non-white children/adolescents. According to previous studies, non-white children/adolescents are less likely to receive adequate mental health treatment [45], including lower likelihood of psychopharmacological prescriptions [46], compared with white children/adolescents.

The economic impact of adolescent mental health care by sectors

We found that the health sector was clearly the main sector accessed by youth with mental disorders. Within the health sector, specialty mental health care was used more frequently and was more costly than primary care. In Brazil, access to CAPS does not require any referral. However, the number of CAPS services are limited, and they are focused on

treatment of severe mental disorders [24]. [illegible] high costs incurred by the mental health sector for the treatment of psychiatric disorders in CAPS may be a result of both, the severity of patients consulting these services and because these services provide intensive outpatient treatments (reflected by the highest number of visits we found), which is costly compared with no-specialized services. It is important to highlight that the lack of youth-oriented primary care mental health services in Brazil limits access to treatment. This could explain why we found low frequency of mental health-related contacts with GP/ family doctors. As a result, contact with specialist mental health services only happens when the disorder has significant negative impact on the lives of young people. In this sense, the organisation of a mental health network of care for adolescents, integrating primary care, social care, education, criminal justice and community youth-specialist services, according to the impact of cases, must be considered in Brazil to adequately plan and allocate scarce public budgets [47].

We found that mental-health related educational service use was less prevalent compared with health service use, nevertheless –as previous studies have shown– [11,14] educational service use was also associated with higher costs. In Brazil, while special education services are provided in regular schools, their use is restricted to students with disabilities and developmental disorders [48], so only adolescents with severe mental disorders are likely to be eligible.

Limitations

Our study has several limitations. First, the psychometric properties of the adapted version of the SACA have not been evaluated yet. Second, as we were not able to access administrative records, service use assessment was limited to guardians' reports. However, the concordance between parent report and records for service use on the parental version of the

SACA is strong [36]. Third, as most of the unit cost were specifically identified for the cities where the HRC is being conducted, São Paulo and Porto Alegre, they are not necessarily generalisable to the whole country. Fourth, due to the limited number of participants using each type of service, we were unable to compare factors related with use and associated costs of specific types of service. Furthermore, given our estimates come from observational cohort data, we are not able to establish causality.

Conclusions

Our findings suggest that the main drivers of health-related service use costs among adolescents in Brazil were impact of mental health problems, in addition to lower stigma toward people with mental illness among guardians and White ethnicity. In the present study, only 22.4% of young people with a diagnosed mental disorder received any form of care. In addition to reducing inequality in service use among children, our findings also argue for lowering barriers to care, in particular addressing caregiver stigma. Furthermore, because lower use of services in adolescence may be associated with worse outcomes across the life course [47], it is needed to further explore measures to reduce inequalities in service utilisation by young people, even though this implies higher short-term costs.

Guardian's lower stigmatising attitudes towards mental disorders may be crucial to support young people in accessing, engaging and maintaining contact with mental health-related services. Various anti-stigma interventions have demonstrated effectiveness for improving help-seeking [49], but few have been implemented in LMICs. Further studies are needed to design and implement anti-stigma interventions in LMICs. On the other hand, health and education policies need to better support guardians to access appropriate and timely services in their communities, before the symptoms have a significant impact on adolescent functioning. We conclude that the organisation of a culturally sensitive mental health network

of care for adolescents, integrating primary care, social care, education, criminal justice services and CAPS, must be considered in Brazil to adequately plan and allocate scarce public budgets

Financial Support: The research presented in this article was funded by the European Research Council under the European Union's Seventh Framework Programme (FP7/2007-2013)/ERC grant agreement no 337673, and supported by the National Institute of Developmental Psychiatry for Children and Adolescents, a science and technology institute funded by Conselho Nacional de Desenvolvimento Científico e Tecnológico (CNPq; National Council for Scientific and Technological Development; grant numbers 573974/2008-0 and 465550/2014-2) and Fundação de Amparo à Pesquisa do Estado de São Paulo (FAPESP grant number 2008/57896-8 and 2014/50917-0). SEL receives support from the UK Medical Research Council in relation to the Mentalkit-Brazil project (MR/R022763/1) and the Economic and Social Research Council. CZ received a doctoral scholarship and research abroad scholarship by the Fundação de Amparo à Pesquisa do Estado de São Paulo (grant number 2018/05586-7 and 2019/08731-0). MSH was supported by the Newton International Fellowship (Ref: NIF\R1\181942), awarded by the Academy of Medical Sciences through the UK Government's Newton Fund Programme. The funding organisations had no role in the study design; collection, analysis or interpretation of data; in the writing of this article; or in the decision to submit the article for publication.

Conflicts of interests: All authors report no conflict of interest associated with this publication. Luis Augusto Rohde has received grant or research support from, served as a consultant to, and served on the speakers' bureau of Aché, Bial, Medice, Novartis/Sandoz, Pfizer/Upjohn, and Shire/Takeda in the last three years. The ADHD and Juvenile Bipolar Disorder Outpatient Programs chaired by Dr Rohde have received unrestricted educational and research support from the following pharmaceutical companies in the last three years: Novartis/Sandoz and

Shire/Takeda. Dr Rohde has received authorship royalties from Oxford Press and ArtMed. None of these commercial relationships alter our adherence to PLOS ONE policies on sharing data and materials.

Access to data and Data sharing: CZ have full access to all the data used in the study and takes responsibility for the integrity of the data and the accuracy of the data analysis. Data were provided by the Brazilian High-Risk Cohort study and are available upon request in the Open Science Framework public repository (https://osf.io/ktz5h/).

REFERENCES

1. Polanczyk G V., Salum GA, Sugaya LS, Caye A, Rohde LA. Annual Research Review: A meta-analysis of the worldwide prevalence of mental disorders in children and adolescents. J Child Psychol Psychiatry. 2015;56: 345–365. doi:10.1111/jcpp.12381
2. Knapp M, King D, Healey A, Thomas C. Economic outcomes in adulthood and their associations with antisocial conduct, attention deficit and anxiety problems in childhood. J Ment Health Policy Econ. 2011;14: 137–47.
3. Kim-Cohen J, Caspi A, Moffitt TE, Harrington H, Milne BJ, Poulton R. Prior juvenile diagnoses in adults with mental disorder: developmental follow-back of a prospective-longitudinal cohort. Arch Gen Psychiatry. 2003;60: 709–17. doi:10.1001/archpsyc.60.7.709
4. Richards, M; Abbott R. Childhood mental health and life chances in post-war Britain. Insights from three national birth cohort studies. London: The Smith Institute, Sainsbury Centre for Mental Health & Unison; 2009.
5. Chen H, Cohen P, Kasen S, Johnson JG, Berenson K, Gordon K. Impact of Adolescent Mental Disorders and Physical Illnesses on Quality of Life 17 Years Later. Arch Pediatr Adolesc Med. 2006;160: 93. doi:10.1001/archpedi.160.1.93
6. Zechmeister I, Kilian R, McDaid D. Is it worth investing in mental health promotion and prevention of mental illness? A systematic review of the evidence from economic evaluations. BMC Public Health. 2008;8: 20. doi:10.1186/1471-2458-8-20
7. McCrone P, Knapp M, Fombonne E. The Maudsley long-term follow-up of child and adolescent depression. Eur Child Adolesc Psychiatry. 2005;14: 407–413. doi:10.1007/s00787-005-0491-6
8. Knapp M; Evans-Lacko S. Health Economics. 6th ed. In: Thapar A, Pine D, editors. Rutter’s Child and Adolescent Psychiatry. 6th ed. Wiley-Blackwell; 2015. pp. 227–238.

9. Knapp M, Wong G. Economics and mental health: the current scenario. World Psychiatry. 2020;19: 3–14. doi:10.1002/wps.20692

10. Beecham J. Annual Research Review: Child and adolescent mental health interventions: a review of progress in economic studies across different disorders. J Child Psychol Psychiatry. 2014;55: 714–732. doi:10.1111/jcpp.12216

11. Snell T, Knapp M, Healey A, Guglani S, Evans-Lacko S, Fernandez JL, et al. Economic impact of childhood psychiatric disorder on public sector services in Britain: Estimates from national survey data. J Child Psychol Psychiatry. 2013; 1–9. doi:10.1111/jcpp.12055

12. Funk M, Saraceno B, Lund C, Flisher AJ, Green A, Knapp M, et al. Planning and budgeting to deliver services for mental health. Mental health policy and service guidance package. Geneva: World Health Organization; 2003.

13. Evans-Lacko S, DosReis S, Kastelic E, Riley A. Mental Health Service Use Before and After Diagnosis of Early-Onset Bipolar Disorder. J Behav Health Serv Res. 2011;38: 398–413. doi:10.1007/s11414-010-9225-8

14. Knapp M, Snell T, Healey A, Guglani S, Evans-Lacko S, Fernandez J-L, et al. How do child and adolescent mental health problems influence public sector costs? Interindividual variations in a nationally representative British sample. J Child Psychol Psychiatry. 2015;56: 667–676. doi:10.1111/jcpp.12327

15. Beecham J, Green J, Jacobs B, Dunn G. Cost variation in child and adolescent psychiatric inpatient treatment. Eur Child Adolesc Psychiatry. 2009;18: 535–542. doi:10.1007/s00787-009-0008-9

16. Gronholm PC, Ford T, Roberts RE, Thornicroft G, Laurens KR, Evans-Lacko S. Mental Health Service Use by Young People: The Role of Caregiver Characteristics. PLoS One. 2015;10: e0120004. doi:10.1371/journal.pone.0120004

17. Clement S, Schauman O, Graham T, Maggioni F, Evans-Lacko S, Bezborodovs N, et al. What is the impact of mental health-related stigma on help-seeking? A systematic review of quantitative and qualitative studies. Psychol Med. 2015;45: 11–27. doi:10.1017/S0033291714000129

18. Kamaradova D, Latalova K, Prasko J, Kubinek R, Vrbova K, Krnacova B, et al. Connection between self-stigma, adherence to treatment, and discontinuation of medication. Patient Prefer Adherence. 2016;10: 1289–1298. doi:10.2147/PPA.S99136

19. Horton S, Toledo ED la C, Mahon J, Santelli J, Waldfogel J. Identifying an Essential Package for Adolescent Health: Economic Analysis. Child and Adolescent Health and Development. Washington, D.C.: The World Bank; 2017. doi:10.1596/978-1-4648-0423-6_ch26

20. Fatori D, Salum G, Itria A, Pan P, Alvarenga P, Rohde LA, et al. The economic impact of subthreshold and clinical childhood mental disorders. J Ment Heal. 2018;27: 588–594. doi:10.1080/09638237.2018.1466041

21. Paula CS, Bordin IAS, Mari JJ, Velasque L, Rohde LA, Coutinho ESF. The Mental Health Care Gap among Children and Adolescents: Data from an Epidemiological

Survey from Four Brazilian Regions. PLoS One. 2014;9: e88241. doi:10.1371/journal.pone.0088241

22. Ministério da Educação. Notas Estatísticas Censo Escolar 2018. 2019 [cited 20 Sep 2019]. Available: http://download.inep.gov.br/educacao_basica/censo_escolar/notas_estatisticas/2018/notas_estatisticas_censo_escolar_2018.pdf

23. Agência Nacional de Saúde Suplementar. Beneficiários de planos privados de saúde, por cobertura assistencial (Brasil – 2010-2020). 2020 [cited 15 Apr 2020]. Available: http://www.ans.gov.br/index.php/aans/quem-somos/227-dados-gerais

24. Paula CS, Lauridsen-Ribeiro E, Wissow L, Bordin I a. S, Evans-Lacko S. How to improve the mental health care of children and adolescents in Brazil: Actions needed in the public sector. Rev Bras Psiquiatr. 2012;34: 334–341. doi:10.1016/j.rbp.2012.04.001

25. Salum GA, Gadelha A, Pan PM, Moriyama TS, Graeff-Martins AS, Tamanaha AC, et al. High risk cohort study for psychiatric disorders in childhood: rationale, design, methods and preliminary results. Int J Methods Psychiatr Res. 2015;24: 58–73. doi:10.1002/mpr.1459

26. Weissman MM, Wickramaratne P, Adams P, Wolk S, Verdeli H, Olfson M. Brief Screening for Family Psychiatric History: The Family History Screen. Arch Gen Psychiatry. 2000;57: 675–682. doi:10-1001/pubs.Arch Gen Psychiatry-ISSN-0003-990x-57-7-yoa8214

27. Associação Brasileira de Empresas de Pesquisa. Critério de Classificação Econômica Brasil. 2018 [cited 15 Apr 2020]. Available: http://www.abep.org/criterio-brasil

28. Goodman R, Ford T, Richards H, Gatward R, Meltzer H. The Development and Well-Being Assessment: description and initial validation of an integrated assessment of child and adolescent psychopathology. J Child Psychol Psychiatry. 2000;41: 645–55.

29. Fleitlich-Bilyk B, Goodman R. Prevalence of Child and Adolescent Psychiatric Disorders in Southeast Brazil. J Am Acad Child Adolesc Psychiatry. 2004;43: 727–734. doi:10.1097/01.chi.0000120021.14101.ca

30. Sourander A, Helstelä L, Helenius H. Parent-adolescent agreement on emotional and behavioral problems. Soc Psychiatry Psychiatr Epidemiol. 1999;34: 657–663. doi:10.1007/s001270050189

31. Salbach-Andrae H, Klinkowski N, Lenz K, Lehmkuhl U. Agreement between youth-reported and parent-reported psychopathology in a referred sample. Eur Child Adolesc Psychiatry. 2009;18: 136–143. doi:10.1007/s00787-008-0710-z

32. Martel MM, Pan PM, Hoffmann MS, Gadelha A, do Rosário MC, Mari JJ, et al. A general psychopathology factor (P factor) in children: Structural model analysis and external validation through familial risk and child global executive function. J Abnorm Psychol. 2017;126: 137–148. doi:10.1037/abn0000205

33. Stringaris A, Goodman R. The Value of Measuring Impact Alongside Symptoms in Children and Adolescents: A Longitudinal Assessment in a Community Sample. J Abnorm Child Psychol. 2013;41: 1109–1120. doi:10.1007/s10802-013-9744-x

34. Evans-Lacko S, Rose D, Little K, Flach C, Rhydderch D, Henderson C, et al. Development and psychometric properties of the Reported and Intended Behaviour Scale (RIBS): a stigma-related behaviour measure. Epidemiol Psychiatr Sci. 2011;20: 263–271. doi:10.1017/S2045796011000308

35. Ribeiro WS, Gronholm PC, Silvestre de Paula C, Hoffmann MS, Rojas Vistorte AO, Zugman C, et al. Development and validation of the Brazilian Portuguese Version of the Reported and Intended Behaviour Scale (RIBS-BP). Stigma Heal. 2020;In press. doi:10.1037/sah0000224

36. Hoagwood K, Horwitz S, Stiffman A, Weisz J, Bean D, Rae D, et al. Concordance Between Parent Reports of Children's Mental Health Services and Service Records: The Services Assessment for Children and Adolescents (SACA). J Child Fam Stud. 2000;9: 315–331. doi:10.1023/A:1026492423273

37. Horwitz SM, Hoagwood K, Stiffman AR, Summerfeld T, Weisz JR, Costello EJ, et al. Reliability of the Services Assessment for Children and Adolescents. Psychiatr Serv. 2001;52: 1088–1094.

38. Banco Central do Brasil. Conversor de moedas. 2018 [cited 10 Oct 2019]. Available: https://www.bcb.gov.br/conversao

39. Razzouk D. Accommodation and Health Costs of Deinstitutionalized People with Mental Illness Living in Residential Services in Brazil. PharmacoEconomics - Open. 2019;3: 31–42. doi:10.1007/s41669-018-0078-z

40. Becker P, Razzouk D. Cost of a community mental health service: a retrospective study on a psychosocial care center for alcohol and drug users in São Paulo. Sao Paulo Med J. 2018;136: 433–441. doi:10.1590/1516-3180.2018.0164310818

41. Banco Central do Brasil. Calculadora do Ciudadão. Correção de valores. 2018 [cited 10 Sep 2019]. Available: https://www3.bcb.gov.br/CALCIDADAO

42. Mihaylova B, Briggs A, O'Hagan A, Thompson SG. Review of statistical methods for analysing healthcare resources and costs. Health Econ. 2011;20: 897–916. doi:10.1002/hec.1653

43. Villatoro AP, DuPont-Reyes MJ, Phelan JC, Painter K, Link BG. Parental recognition of preadolescent mental health problems: Does stigma matter? Soc Sci Med. 2018;216: 88–96. doi:10.1016/j.socscimed.2018.09.040

44. Blair C, Raver CC. Poverty, Stress, and Brain Development: New Directions for Prevention and Intervention. Acad Pediatr. 2016;16: S30–S36. doi:10.1016/j.acap.2016.01.010

45. Alegria M, Vallas M, Pumariega AJ. Racial and Ethnic Disparities in Pediatric Mental Health. Child Adolesc Psychiatr Clin N Am. 2010;19: 759–774. doi:10.1016/j.chc.2010.07.001

46. Leslie LK, Weckerly J, Landsverk J, Hough RL, Hurlburt MS, Wood PA. Racial/Ethnic Differences in the Use of Psychotropic Medication in High-Risk Children and Adolescents. J Am Acad Child Adolesc Psychiatry. 2003;42: 1433–1442. doi:10.1097/00004583-200312000-00010

47. Knapp M, Ardino V, Brimblecombe N, Evans-Lacko S, Lemmi V, King D, et al. Youth mental health: new economic evidence. London: Personal Social Services Research Unit; 2016.

48. Ministério da Educação. Decreto Nº 7.611. 2011 [cited 20 Sep 2019]. Available: http://www.planalto.gov.br/ccivil_03/_Ato2011-2014/2011/Decreto/D7611.htm#art11

49. Thornicroft G, Mehta N, Clement S, Evans-Lacko S, Doherty M, Rose D, et al. Evidence for effective interventions to reduce mental-health-related stigma and discrimination. Lancet. 2016;387: 1123–1132. doi:10.1016/S0140-6736(15)00298-6

Supporting information

S1 Fig. Flow chart of Brazilian High-Risk Cohort participants included in the mental-health related service use study.

S1 Table. Unit costs of health, educational, social care and criminal justice related services.

S2 Table. Bivariate analysis: Predictors of 12-month mental health service utilisation.

S3 Table. Bivariate analysis: Predictors of cost of mental health service use in the last 12 months.

S4 Table. Logistic regression models: 12-month mental health service utilisation predicted by psychiatric diagnosis trajectories.

S5 Table. Logistic regression models: 12-month mental health service utilisation predicted by impact of behavioural and emotional difficulties on child' life.

S6 Table. Logistic regression models: 12-month mental health service utilisation predicted by parental stigma.

S7 Table. Generalised linear models: cost of 12-month mental health service utilization predicted by psychiatric diagnosis trajectories.

S8 Table. Generalised linear models: cost of 12-month mental health service utilisation predicted by impact of behavioural and emotional difficulties on child' life.

S9 Table. Generalised linear models: cost of 12-month mental health service utilisation predicted by parental stigma.

S10 Table. Generalised linear models: cost of 12-month mental health service utilisation predicted by broad diagnosis categories.

S1 Figure

Click here to access/download
Supporting Information
S1_Fig.pdf

S1 Table

Click here to access/download
Supporting Information
S1_Table.pdf

S2 Table

Click here to access/download
Supporting Information
S2_Table.pdf

S3 Table

Click here to access/download
Supporting Information
S3_Table.pdf

S4 Table

Click here to access/download
Supporting Information
S4_Table.pdf

S5 Table

Click here to access/download
Supporting Information
S5_Table.pdf

S6 Table

Click here to access/download
Supporting Information
S6_Table.pdf

S7 Table

Click here to access/download
Supporting Information
S7_Table.pdf

S8 Table

Click here to access/download
Supporting Information
S8_Table.pdf

S9 Table

Click here to access/download
Supporting Information
S9_Table.pdf

S10 Table

Click here to access/download
Supporting Information
S10_Table.pdf

Utilisation and costs of mental health-related service use among adolescents

Carolina Ziebold, PhD[1], Wagner Ribeiro, PhD[1,2], Derek King, PhD[2], David McDaid, MSc[2], Mauricio Hoffmann, PhD[2,3,4,5], Renee Romeo, PhD[6], Pedro Pan, PhD[1,5], Euripides Miguel, PhD[5,7], Rodrigo Bressan, PhD[1,5], Luis Augusto Rohde, PhD[5,8,9], Giovanni Salum, PhD[5,98], Jair Mari, PhD[1,5], and Sara Evans-Lacko, PhD[2*]

[1] Departamento de PsiquiatriaUniversidade Federal de São Paulo, Departamento de Psiquiatria, Universidade Federal de São Paulo, São Paulo, Brazil

[2] Care Policy and Evaluation Centre, London School of Economics and Political Science, London, United Kingdom

[3] Universidade Federal de Santa Maria, Santa Maria, Brazil

[4] Hospital de Clínicas de Porto Alegre, Porto Alegre, Brazil

[5] National Institute of Developmental Psychiatry for Children and Adolescents, São Paulo, Brazil

[6] King's College London, London, United Kingdom

[7] Universidade de São Paulo, São Paulo, Brazil

[8] ADHD Outpatient Program & Developmental Psychiatry Program, Hospital de Clínicas de Porto Alegre, Brazil.

[9] Universidade Federal do Rio Grande do Sul, Porto Alegre, Brazil

*****Corresponding author: Sara Evans-Lacko, Care Policy and Evaluation Centre, London School of Economics and Political Science. Email: S.Evans-Lacko@lse.ac.uk (SEL)

Abstract

Background: The high burden of care for adolescents with mental health disorders represents a challenge to the public sector, especially in low and middle-income countries. We aimed to estimate the costs to the public purse of health, education, criminal justice and social care service use associated with psychiatric disorders among adolescents in Brazil; and to examine whether the trajectory of psychopathology and its impact on daily life, and parental stigma towards mental illness, may be associated with service utilisation and costs.

Methods: Data on reported service use among adolescents from a prospective community cohort (n=1,400) were combined with Brazilian unit costs. Logistic regression and generalised linear models were used to examine predictors of service use and associated costs, respectively.

Results: Twenty-two percent of those who presented with a psychiatric disorder used some type of service for their mental health in the previous twelve months. Higher odds of service use were associated with having ~~an incident~~a diagnosed mental disorder (either incident, [~~(~~OR=2.49, 95%CI=1.44-4.30, p=0.001]~~)~~, remittent [~~(~~OR=2.16, 95%CI=1.27-3.69, p=0.005]~~)~~ or persistent [~~(~~OR=3.01, 95%CI=1.69-5.36, p<0.001])~~) psychiatric disorder~~, higher impact of symptoms on adolescent's life (OR=1.32, 95%CI=1.19-1.47, p<0.001) and lower parental stigma toward mental illness (OR=1.12, 95%CI=1.05-1.20, p=0.001). Average annual cost of service use was 527.14 USD (s.d.= 908.10). Higher cost was predicted by higher disorder impact (β=0.25, 95%CI=0.12-0.39, p<0.001), lower parental stigma (β=0.12, 95%CI=0.02–0.23, p=0.020) and white ethnicity (β=0.55, 95%CI=0.04–1.07, p=0.036).

Conclusion: The impact of emotional and behavioural symptoms on adolescents' lives and parental stigmatising attitudes toward mental illness were the main predictors both of service use and costs.

Key words: Service Utilisation, Mental Health, Adolescence, Health Economics

Introduction

Mental health conditions affect 13.4% of children and adolescents globally, representing the leading cause of disability in this age group [1]. They can have long-term impacts on health and social outcomes into adulthood [2–7]. The high prevalence and potentially enduring nature of these impacts make addressing youth mental health conditions particularly important, but this is a challenge for public systems with limited resources [8]. ~~Provision of mental health care~~Economic costs associated with youth mental health conditions ~~-~~ involve a wide range of sectors including health, educational, social care, and criminal justice services [9,10]. This can represent a substantial cost to the public purse, yet it could also be considered a wise investment given the evidence that effective treatment can mitigate the impact of poor mental health [2]. Estimating the economic cost of mental disorders in young people from the perspective of the public purse and understanding which factors are associated with these costs could support more effective and efficient policy planning and care delivery [8,11,12].

Formatted: Font color: Red

Some studies from high–income countries suggest that ~~male gender, older age and~~ lower socioeconomic status, as well as clinical features (illness severity and impact of disorders) are associated with use of health, special education, and social care services, while male gender and older age ~~and~~ are associated with more criminal justice services contacts [11,13,14]. These sociodemographic and clinical characteristics are also ~~and greater~~ associated with greater mental health-related treatment costs among young people [11,14,15]. Families also play a central role in young people's contact with services. One study from the UK found that lower mental illness-related stigma among caregivers was associated with an increased likelihood of young people's mental health service use [16]. Stigmatising attitudes toward mental illness amongst parents may influence service contacts due to shame and fears of labelling their child's mental health condition [16]. ~~However, little is known about how parental stigma could impact~~

~~on costs which also reflect intensity of service use.~~ There are clear links between stigma and reduced~~Studies among adults suggest that stigma reduces~~ help-seeking [17]~~, and increases non-~~ reduced adherence to treatment and early withdrawal from services [17,18]. However, little is known about how parental stigma could impact on young people service use and costs~~ which also reflect intensity of service use.~~

Most costing studies have focused on a single disorder, commonly autism, attention deficit hyperactivity disorder or conduct disorders [10]. Additionally, little is known about how, ~~beyond~~ in addition to the type of ~~diagnosis~~disorder, whether persistence of psychopathology from childhood to adolescence, ~~and~~ disorders' impact on adolescent's daily life (i.e. functioning), ~~in addition to~~and key barriers to care such as stigma, could influence costs.

Formatted: Font color: Red

Formatted: Font color: Red

There are a limited number of studies reporting on prevalence of mental health service use in low and middle-income countries (LMICs) [19–21], however, none use validated service use measures. Moreover, prevalence of any use does not capture the intensity of use (e.g. number or type of visits) needed to understand the economic impact of child mental health problems. From a global mental health perspective, examining this issue in a LMIC context, where resources are scarce, is of major significance. ~~Similar to most high-income countries,~~ Brazil provides universal access to health services and education for the entire population that is free at the point of use, while private health care and education are used by about 20% of the population [22–24]. Estimating the economic cost of mental disorders among young people to the public purse, and understanding which factors are associated with these costs in Brazil is essential for public policy planning, specifically to optimise investment. This approach could also be of value for similar health and welfare systems.

Furthermore, examining the variation in costs according to clinical characteristics of adolescents, beyond type of diagnosis, is important as the impact of psychopathology on daily

life and the trajectory of psychopathology from childhood to adolescence, may support service planning and resource allocation in relation to clinical characteristics in a preventive and responsive way.

The aim of this study is to estimate the costs associated with health, education, criminal justice and social care services among a cohort of young people in Brazil. We first present the annual aggregate cost to the public purse and then disaggregate this impact to reflect and understand the relative costs to different sectors. Second, we examine how costs vary according to: mental health ~~child~~trajectories, impact of the disorder on everyday life, and parent/guardian stigma towards mental illness~~characteristics~~. We hypothesise that persistence of psychiatric disorders from childhood to adolescence and associated impact on adolescents' lives have the greatest influence on costs. However, we also expect that lower levels of parental stigma towards mental illness will predict greater likelihood of service use and hence higher costs.

Formatted: Font color: Red

Formatted: Font color: Red

Methods

Data and participants

This study is nested within the Brazilian High-Risk Cohort (BHRC), which is an ongoing prospective longitudinal study that comprises a community sample and a high-risk sub-sample (a sample ~~with children~~ at increased risk of mental disorders) of young people from Sao Paulo and Porto Alegre, Brazil. ~~, who were six to twelve years old at baseline (2010-2011, n=2,511).~~ A detailed description of the sample and procedures can be found elsewhere [25]~~)~~. Briefly, during the registry day, 12,500 parents of young people aged 6 to 14 years attending 57 schools (22 in Porto Alegre and 35 in São Paulo) were invited to a screening of mental health disorders using the Family History Screen (FHS) [26]. A total of 8,012 families (9,937 eligible children, 45,394 family members) were interviewed. Based on the percentage of members in

Formatted: Font color: Red

the family that screened positively for psychiatric disorders, an index of family load for each potential eligible child was computed. The final cohort comprised 2,511 young people; 957 were randomly selected, and 1,554 were a sub-sample at increased risk of mental disorders based on the FHS. Cohort participants were interviewed at baseline (aged 6-14 years, calendar year:2010-2011, n=2,511), and at first follow-up (N=2010, aged 9-17 years, calendar year 2014).

After completing the BHRC first follow-up interview~~(2014-2015, child participants aged 10-18 years)~~, 1,881 parents/guardians were invited to respond to a ~~comprehensive~~ supplementary interview which included a comprehensive assessment of mental health related service use (calendar year: 2014-2015, young people participants aged 10-18 years). Among those contacted, 1,400 (74.4%) guardians (in 93.1% of cases the biological mother) completed the interview –982 (70.1%) by telephone and 418 (29.9%) face-to-face– (See flow chart in S1 Fig.). There were no significant differences in persistence of psychopathology or impact of psychopathology on adolescents' lives among respondents versus non-respondents.

Formatted: Font color: Red

This research was carried out in accordance with the latest version of the Declaration of Helsinki. ~~Child assent and P~~parental written informed consent was obtained from all the research subjects. Young people provided verbally informed assent (documented as part of the consent form, and witnessed by the interviewer), and those who were able to read and write also provided written consent. All procedures were approved by the Ethics Committee of the Federal University of São Paulo-UNIFESP (Nº 2.879.533 and ~~–~~CAAE 06457219.9.0000.5505), Hospital de Clínicas de Porto Alegre (CAAE 06457219.9.3001.5327) and the European Research Commission.

Formatted: Font color: Red

Data were provided by the Brazilian High-Risk Cohort study and are available upon request in the Open Science Framework public repository (https://osf.io/ktz5h/).

Measures

Sociodemographic Characteristics

Data on the following sociodemographic characteristics were collected: gender, age at follow-up, ethnicity (white and non-white: black, Asian, indigenous or mixed-race), socioeconomic group (SEG), and maternal educational level (no/basic, secondary or university education). SEG was defined according to a Brazilian standardized questionnaire [27]. Based on families' assets and head of household's education level, a total score ranging for 0 to 46 is given, where greater scores represent higher socioeconomic status. In this study, SEG was categorised as "low" (0-22) and "high" (23-46).

Psychopathology

Psychiatric diagnosis: Psychiatric diagnoses were assessed at baseline and follow-up using the Brazilian-Portuguese version of the Development and Well-being Assessment (DAWBA) [28,29], which is a highly structured interview used to generate DSM-IV diagnoses. Trained interviewers gathered information on current problems causing significant distress or social impairment. At baseline, diagnostic assessment and interviews were performed with guardians only. Previous literature has found that self-report on internalising conditions during adolescence is higher compared with parental report. This can be explained because internalising problems, such as anxiety or depression, would be less observable by guardians, being advisable to consider both reports to reach a reliable evaluation of adolescent mental health [30,31]. For this reason, diagnostic assessment at 3-year follow-up ~~At 3-year follow-up, diagnostic assessment~~ was performed considering guardian reports and additional information

from interviews with the young people about internalising conditions. Computerised diagnostic probabilities were then generated based on responses those were carefully evaluated by 9 trained psychiatrists who determined the diagnosis.

Broad psychiatric diagnostic categories: Based on previous literature [32], follow-up DAWBA diagnoses were grouped into three broad categories: distress-related disorders (including depression, generalised anxiety disorder, obsessive – compulsive disorder, tic, eating disorder), fear-related disorders (including panic, agoraphobia, social anxiety, specific phobia and separation anxiety) and externalising disorders (including conduct disorder, oppositional defiant disorder and attention deficit/hyperactivity disorder).

Persistence of diagnosis: Four categories of diagnostic persistence were created based on presence of diagnosis at baseline and/or follow-up: 1) no diagnosis (no diagnosis at both time points), 2) incident (no diagnosis at baseline and presence of diagnosis at follow-up), 3) remittent (presence of diagnosis at baseline and no diagnosis at follow-up), 4) persistent (presence of diagnosis at both time points).

Impact of mental health problems at follow-up: was measured according to the 'impact supplement' of the Strength and Difficulties Questionnaire (SDQ) which is part of DAWBA. This supplement assesses the impact of behavioural and emotional difficulties on ~~children's~~ adolescent's lives according to guardian reports. A total score (0-10) was generated by summing 5 items: distress, social impairment in family life, friendships, learning, and leisure activities [33]. Higher scores represent greater impact. The impact score has demonstrated internal consistency, cross-informant correlations, and stability measured across time [33].

Parent-reported stigma towards mental health problems

To assess parental stigma, we applied the Brazilian Portuguese version of the Reported and Intended Behaviour Scale (RIBS-BP) [34,35]. The intended behaviour subscale assesses future intended stigmatising behaviour across four domains: living with, working with, living nearby and continuing a relationship with someone with a mental health problem. Higher scores represent lower stigma. The RIBS-BP has demonstrated good internal consistency, and good to excellent construct validity [35].

Service use

The Service Assessment for Children and Adolescents (SACA) [36] was used to ask guardians about service contacts made in the past 12 months in response to concerns regarding their child's emotions and behaviour, including alcohol and drugs. The SACA assesses type, nature, frequency and duration of services used, treatments received and settings in which services were delivered. Overall concordance between parent report and records (kappa=0.76) [36] and test-retest reliability for 12-month (kappa=0.75–0.86) service use on the parent version of the SACA is strong [37].

We received permission from the SACA developers to translate and adapt the instrument to the Brazilian context in consultation with experts in the Brazilian mental health system to ensure we covered the relevant service types and settings in Brazil. The list of services and professionals was grouped into three sectors: 1) health care: inpatient services (psychiatric hospital, psychiatric unit in a general hospital, alcohol and drug clinic); outpatient services (Centre for psychosocial care [CAPS], which are the community mental health services in Brazil; mental health clinics; specialist mental health professionals (psychiatrists and

psychologists in settings other than CAPS and mental health clinics); general health services and professionals (emergency room, paediatrician, general practitioner [GP] or family doctor); 2) education: special school and special education in regular school (special room and special needs class assistant); 3) social care and criminal justice: overnight stay in a shelter or detention centre; probation programme contact; and home visit of the guardianship council (services responsible for child-rights protection).

Estimation of costs

Data collected on use of services from the BHRC were combined with unit costs to derive service use costs in Brazilian Reals for the financial year 2018 and then converted to US dollars (based on December 31 2018 conversion rate 1 Real=0.2581 dollars, according to the Brazilian Central Bank) [38].

Unit costs: Detailed information on source of information and unit cost values for each service is available in S1 Table. Where available, we applied unit costs previously reported in the Brazilian literature [39,40]. However, as costs of many services have not previously been reported, we performed a thorough consultation process gathering relevant data from public databases of the Ministries of Education and Health, and the social care departments of the municipalities of Porto Alegre and São Paulo (S1 Table).

Unit costs were attached to data on service use frequencies for each type of service (based on the SACA) based on 2018 prices or the latest available year converted to 2018 prices using the Nationwide Consumer Price Index. The Brazil Central Bank's calculator was used to apply the index [41]. Once obtained, information on the unit cost of each service was used to calculate the total annual cost by sector (health, education, social care and criminal justice) for each participant by multiplying the frequency of use (e.g. number of visits, nights) by unit cost.

Data Analysis

Data were analysed using STATA, version 14. First, we described prevalence of socio-demographic and clinical characteristics overall and by persistence of psychopathology. Between-group differences were compared using chi-squared tests. For interval variables, means and standard deviations were calculated and overall significance was tested using one-way analysis of variance. A significance parameter of $p < .05$ (two-tailed) was applied for all tests.

Unadjusted odds ratios and coefficients for each predictor and covariate in relation to mental health service use and costs are presented in S2 and S3 Tables. To compare the relative impact between our three main predictors (i.e., psychopathological trajectories, impact of the disorder and parental stigma) of service use and costs we also present logistic regression models for each of these variables adjusting for sociodemographic characteristics (gender, age, mother's education, ethnicity and SEG) and dummy variables (mode of data collection and city of residence) (S4-S6 Tables for service use and S7-S9 Tables for costs). We then used multivariable analyses to examine the association between guardian and adolescent characteristics with service use (logistic regression models) and associated costs (generalised linear models – GLM), overall and by sector: 1) health; 2) education; and 3) social care and criminal justice. All multivariable analyses were adjusted by socio-demographic characteristics, mode of data collection and city.

For costs GLM, we analysed the subset of participants who used services in the previous 12 months (n=143). Annual costs for each participant were included in the models as a scalar dependent variable, with a Gamma distribution [42], using the log-link function.

Results

Table 1 describes sociodemographic and clinical characteristics of participants. The sample comprised 1,400 adolescents with a mean age of 14 years (s.d=1.98). The majority were white males from low SEG, and only 10% of mothers had university education.. 23.3% (n= 326) of adolescents had a psychiatric disorder in the previous 12 months, of which 177 (54.3%) were incident and 149 (45.7%) persistent cases since baseline. 213 (15.2%) participants had remitted from a baseline psychiatric diagnosis. Participants with externalising disorders were more likely to have persistent trajectories (RR=2.19, 95%CI=1.38-3.48, p<0.001). Participants categorised as persistent also reported greater disorder impact (β=2.34, 95%CI=2.11-2.58, p<0.001). 22.4% of those who presented with a psychiatric disorder reported usinged some type of service for their mental health in the previous twelve months. The. proportion of service use among those who presented a persistent psychiatric condition was 27%. Table 1 also describes the mean costs of mental health-related service use in the past year, by psychiatric trajectory (from no diagnosis to persistent psychiatric diagnosis). Bivariate analyses showed a non-significant association between psychiatric trajectory and mean annual costs.

Table 1. Sociodemographic and clinical characteristics by trajectories of psychopathology (n=1,400).

	**No psychiatric diagnosis (n=861)**	**Incident psychiatric diagnosis (n=177)**	**Remittent psychiatric diagnosis (n=213)**	**Persistent psychiatric diagnosis (n=149)**	**Overall sample (n=1,400)**	
	N (%)	N (%)	N (%)	N (%)	N (%)	p
Sociodemographic characteristics						
Male gender	503 (58.4)	81 (45.8)	134 (62.9)	83 (55.7)	801 (57.2)	**0.005**
Female gender	358 (41.6)	96 (54.2)	79 (37.1)	66 (44.3)	599 (42.8)	
Age, mean (s.d)	14.50 (2.02)	14.58 (1.90)	14.39 (1.88)	14.67 (1.99)	14.51 (1.98)	0.564
High SEG	359 (41.7)	63 (35.6)	71 (33.3)	61 (40.9)	554 (39.6)	0.095
Low SEG	502 (58.3)	114 (64.4)	142 (66.7)	88 (59.1)	846 (60.4)	
White ethnicity	484 (56.2)	106 (60.2)	116 (54.5)	84 (57.1)	790 (56.6)	0.704
Non-White ethnicity	377 (43.8)	70 (39.8)	97 (45.5)	63 (42.9)	607 (43.5)	
Guardians characteristics						
Maternal no/basic education	387 (45.1)	78 (44.6)	96 (45.3)	59 (39.9)	620 (44.5)	0.953
Maternal secondary education	384 (44.8)	78 (44.6)	93 (43.9)	71 (48.0)	626 (44.9)	
Maternal university education	87 (10.14)	19 (10.9)	23 (10.9)	18 (12.2)	147 (10.6)	
Clinical characteristics						
Any Psychiatric Diagnosis	-	177 (54.3)	-	149 (45.7)	326 (23.3)	**<0.001**
Fear-related	-	92 (52.0)	-	72 (48.3)	164 (11.7)	**<0.001**
Distress-related	-	70 (40.0)	-	60 (40.3)	130 (9.3)	**<0.001**
Externalising	-	49 (27.7)	-	68 (45.6)	117 (8.4)	**0.001**
SDQ impact mean score (s.d)	0.28 (0.73)	1.49 (1.91)	0.78 (1.51)	2.62 (2.41)	0.78 (1.52)	**<0.001**
Mental health-related service use						
12-months service use	43 (5.0)	32 (18.0)	27 (12.7)	41 (27.5)	143 (10.21)	**<0.001**
Mean service use costs USD$ (s.d)	326.41 (395.53)	581.90 (1360.19)	644.35 (795.50)	628.50 (901.02)	527.14 (908.10)	0.400

Notes: Results in bold are significant. SEG, socioeconomic group; SDQ, Strength and Difficulties Questionnaire. 3 missing data in ethnicity variable, 10 missing data in maternal education variable.

Frequency of mental health-related service use and annual service use costs

Utilisation of mental health services in the previous 12 months and associated cost by type of service are presented in Table 2. Overall, 10.0% of the sample (n=143) used some sort of health, education, criminal justice or social care service for mental health problems. Disaggregating by sectors, the health sector had highest proportion of service users (9%), while the education and social care and criminal justice sectors were less frequently contacted with a 1.8% and 1.3% of users respectively~~,~~. ~~-~~Outpatient mental health services, most notably psychologists and psychiatrists in settings other than community mental health clinics, were the most ~~prevalent~~frequently used ~~among all~~ services/professionals. Inpatient services and ~~G~~general health services such as GP/family doctor, paediatrician and emergency department, were less frequently used. In the education sector, school assistant was the most type of service used by young people, while guardianship council was the most frequently social care service contacted. The total cost of 12-month mental health-related service use for the public purse was 70,110.23 USD. The sector that presented higher total annual cost was the health sector, followed by the education and finally the social care and criminal justice sectors. The services that generated the greatest total costs for the heath sector were psychologist (11,339.64 USD) and CAPS (9,628.01 USD). Among those who used services, the average annual cost of service use amounted to 527.14 USD (SD= 908.10 USD, range=8.77- 7,605.58 USD, median=221.10 USD, interquartile range=545.28) per user.~~. Outpatient mental health services, most notably psychologists and psychiatrists in settings other than community mental health clinics, were the most prevalent among all services/professionals. General health services such as GP/family doctor, paediatrician and emergency department, were less frequently used.~~ Individuals using CAPS (specialty mental health) services (1.1% of the sample) had the highest mean number of

visits during the previous year and the highest associated costs among health services. The second highest mean costs in the health sector were related to hospitalizations in -psychiatric hospitals and alcohol and drugs clinics, while the lowest mean costs were attributed to emergency department, paediatrician, outpatient alcohol and drugs and GP/family doctor contacts. Although only 0.1% of individuals used shelters, this type of social service had the highest associated mean cost. Education services were used by 1.8% of individuals and these services had the second highest associated mean costs.

Table 2. 12-month mental health-related service use and costs by type of service (n = 143).

	Users	Number of visits/ nights	Number of nights/visits per user[a][~~1~~]	Total annual cost per service	Annual cost per user
**Type of service**	n (%)	Total	Mean (Range; s.d.)	~~USD[1]~~USD[a,~~2~~b]	Mean (Range; s.d.)
Health Sector					
Inpatient mental health services[3]					
Psychiatric hospital	7 (0.5)	73	10.6 (1 – 30;11.87)	4,015.72	573.67 (66.42-1,992.72;691.76)
Psychiatric unit in general hospital	1 (0.1)	1	1 (1)	40.49	40.49
AD clinic	3 (0.2)	48	16 (6 – 27;10.73)	1,767.90	589.30 (191.87-1,096.36;462.10)
Outpatient mental health services					
Centre for psychosocial care (CAPS)	15 (1.1)	452	30.15(1-180;49.38)	9,628.01	740.62 (24.56-4,421.03;1212.73)
Mental Health clinic	17 (1.2)	308	18.13(1-70;19.08)	5,644.64	352.79 (19.46-1,362.50;371.28)
Psychiatrist	33 (2.4)	217	6.56 (1-48;8.95)	5,803.90	181.37(27.64-1,326.60;247.29)
Psychologist	71 (5.1)	1,081	15.23 (1-60;14.97)	11,339.64	171.81(11.28-676.99;168.91)
AD clinic	2 (0.1)	2	1 (1)	14.74	14.74
General Health					
Emergency department	4 (0.3)	9	2.25 (1-4;1.50)	156.0	39.00 (17.34-69.34;26.00)
Paediatrician	3 (0.2)	10	3.33 (2-4;1.16)	120.54	40.18 (24.11-48.22;13.92)
GP/family doctor	5 (0.4)	23	4.60 (2-9;2.97)	403.25	80.65 (35.07-157.80;52.01)
Overall health service use	126 (9.0)			37,679.94	324.83 (11.28-4575.70;590.55)
**Educational sector**[4]					
Special School	7(0.5)		School Year	8,564.92	1,223.56 (1,155.72-1,250.70; 44.53)
Special Class	5 (0.4)		School Year	6,063.55	1,212.71 (1,155.72-1,250.70; 52.02)
School Assistant	12 (0.9)		School Year	14,723.52	1,226.96 (1,155.72-1,250.70; 42.95)
Overall education service use	23 (1.8)			29,351.94	1,276.17 (1,155.72-2,501.40; 270.73)
**Social care and criminal justice sector**					
Shelter	2 (0.1)	210	105 (90-120;21.21)	5,599.95	2,799.98 (2,755.34-2,888.48; 63,12)
Guardianship Council home visit	11 (0.8)	31	2.85 (1-5;1.73)	201.84	25.23 (8.77-43.87;15.15)
Probation programme	8 (0.6)		Six months	1,875.48	234.44
Overall social care and criminal justice related service use	18 (1.3)			4,687.47	334.82 (8.77-2,888.48;1,155.72)
~~**Overall service use**~~	~~143(10.2)~~			~~70,110.23~~	~~527.14 (8.77-7605.58; 908,10)~~

[~~1~~]~~Total~~ [a]Total cost health sector N=116, Total cost education sector, N=23, Total cost social care and criminal justice sector, N=14. Total cost, N=133. Cases with missing values in 'frequency of visits' were not included in costs estimates: CAPS=2, mental health clinic=1, psychiatrist=1, psychologist=5, AD clinic=1, guardianship council=3.

[~~2~~]~~Costs~~ [b]Costs are expressed U.S. Dollars, 2018 prices. Brazilian Central Bank conversion rate: Brazilian Real=0.2581, December 31st 2018[38]

Characteristics associated with mental health-related service use

Having an incident, remittent or persistent psychiatric disorder, as well as the higher impact of behavioural and emotional difficulties on the adolescents' lives and lower parental stigma, all predicted higher odds of any 12-month service use (Table 3). Service contacts in the health sector were also predicted by the same factors. Service use in the educational sector was predicted by impact, lower parental stigma and low SEG. There were no factors significantly associated with use of social care and criminal justice services.

Table 3. Multivariable logistic regression models: Predictors of 12-month mental health service utilisation (n=1,390[1]390[a]).

Predictors	Any service use		Health service use		Education service use		Social care and criminal justice service use	
	OR (95%CI)	p	OR (95%CI)	p	OR (95%CI)	p	OR (95%CI)	p
Sociodemographic characteristics								
Male gender (Ref)	-		-		-		-	
Female gender	0.87 (0.59-1.28)	0.488	0.98 (0.65-1.46)	0.901	0.67 (0.24-.84)	0.435	1.28 (0.48-3.40)	0.627
Age (in years)	1.02 (0.92-1.12)	0.703	1.00 (0.90-1.11)	0.942	0.98 (0.78-1.25)	0.886	1.25 (0.97-1.60)	0.084
High SEG (Ref)								
Low SEG	1.30 (0.86-1.98)	0.211	1.11 (0.72-1.70)	0.646	**4.31 (1.29-14.39)**	**0.018**	2.97 (0.75-11.77)	0.122
White ethnicity (Ref)	-		-		-		-	
Non-White ethnicity	1.17 (0.79-1.72)	0.442	0.97 (0.64-1.46)	0.869	0.53 (0.19-1.48)	0.225	2.59 (0.92-7.28)	0.071
Guardians characteristics								
Maternal no/basic education (Ref)	-		-		-		-	
Maternal secondary education	1.23 (0.82-1.85)	0.315	1.30 (0.84-1.99)	0.238	1.77 (0.66-4.78)	0.257	0.51 (0.17-1.54)	0.233
Maternal university education	1.14 (0.59-2.20)	0.698	1.17 (0.59-2.31)	0.658	1.33 (0.24-7.53)	0.744	1.02 (0.19-5.54)	0.981
Lower parental stigma (RIBS scores)	**1.12 (1.05-1.20)**	**0.001**	**1.11 (1.03-1.18)**	**0.003**	**1.22 (1.01-1.48)**	**0.042**	1.01 (0.94-1.25)	0.251
Clinical characteristics								
No psychiatric diagnosis (Ref)	-		-		-		-	
Incident psychiatric diagnosis	**2.49 (1.44-4.30)**	**0.001**	**2.57 (1.45-4.58)**	**0.001**	2.29 (0.51-10.97)	0.281	2.54 (0.61-10.52)	0.199
Remittent psychiatric diagnosis	**2.16 (1.27-3.69)**	**0.005**	**2.22 (1.25-3.93)**	**0.006**	3.24 (0.84-12.50)	0.087	1.98 (0.45-8.75)	0.369
Persistent psychiatric diagnosis	**3.01 (1.69-5.36)**	**<0.001**	**3.33 (1.82-6.08)**	**<0.001**	2.82 (0.65-12.37)	0.168	3.65 (0.88-15.09)	0.073
SDQ impact score	**1.32 (1.19-1.47)**	**<0.001**	**1.32 (1.19-1.47)**	**<0.001**	**1.51 (1.24-1.84)**	**<0.001**	1.22 (0.97-1.55)	0.096
Test statistics	LR $x^2(13)$= 129.35 p<0.001		LR $x^2(13)$= 122.81, p<0.001		LR $x^2(13)$= 57.46, p<0.001		LR $x^2(13)$= 28.36, p=0.008	
	Pseudo-R^2=0.14		Pseudo-R^2=0.15		Pseudo-R^2=0.25		Pseudo-R^2 =0.15	

[1]From [a]From the total sample, N=1,400, 10 cases had missing data in mother's education and 3 in ethnicity variables. Results in bold are statistically significant (p<0.05). Models adjusted by collection instrument and city.

Characteristics associated with greater mental health related service use costs

When all three sectors were combined into a single total cost variable, greater impact, lower parental stigma and white ethnicity were associated with higher costs (Table 4). Each additional impact score predicted an increase in mean costs of 142.59 USD ($p<0.001$). For parental stigma, each additional RIBS-BP score (indicating lower stigma) increased mean cost by 69.32 USD ($p=0.020$). White ethnicity was associated with having higher mean costs of 295.49 USD ($p=0.036$), compared with non-white participants. No association was found between broad diagnosis categories and costs (S10 Table).

When looking at predictors of costs according to sector, disorder impact was associated with greater health sector service use (predicted mean cost by each impact score= 66.26 USD, $p=0.019$). We did not find any significant association of psychiatric trajectories, impact of disorder or parental stigma with education or social care/criminal justice sectors' costs.

Table 4. Generalised linear models: parental and clinical characteristics associated with cost of mental health service use in the last 12 months, overall and by sector.

**Predictors**	**Any service use N= 131**		**Health service use N=115**		**Education service use N=22**		**Social care and criminal justice service use N=14**	
	β (95%CI)	p	β (95%CI)	p	β (95%CI)	p	β (95%CI)	p
Sociodemographic characteristics								
Male gender (Ref)	-		-		-		-	
Female gender	0.05 (-0.50-0.59)	0.866	0.06 (-0.61-0.73)	0.854	-0.03 (-0.29-0.24)	0.857	14.41 (-5.34-34.17)	0.153
Age (in years)	-0.05 (-0.19-0.10)	0.522	0.06 (-0.14-0.24)	0.572	0.02 (-0.02-0.06)	0.886	-2.11 (-4.87-0.65)	0.133
High SEG (Ref)								
Low SEG	0.47 (-0.08-1.03)	0.092	-0.13 (-0.78-0.53)	0.706	-0.03 (-0.36-0.29)	0.839	3.32 (-8.52-15.15)	0.583
White ethnicity (Ref)	-		-		-		-	
Non-White ethnicity	**-0.55 (-1.07- -0.04)**	**0.036**	-0.12 (-0.75-0.51)	0.707	0.09 (-0.10-0.27)	0.368	-4.28 (-9.80-1.24)	0.129
Guardians characteristics								
Maternal no/basic education (Ref)	-		-		-		-	
Maternal secondary education	0.27 (-0.29-0.82)	0.341	-0.10 (-0.78-0.58)	0.776	-0.07 (-0.23-0.10)	0.418	4.95 (-2.19-12.08)	0.174
Maternal university education	0.003 (-0.90-0.91)	0.995	-0.34 (-1.38-0.69)	0.515	0.40 (-0.07-0.87)	0.094	-	-
Lower parental stigma (RIBS score)	**0.12 (0.12-0.39)**	**0.020**	0.04 (-0.07-0.16)	0.465	0.002 (-0.06-0.06)	0.948	0.05 (-0.98-1.08)	0.922
Clinical characteristics								
No psychiatric diagnosis (Ref)	-		-		-		-	
Incident psychiatric diagnosis	-0.14 (-0.83-0.55)	0.693	0.15 (-0.71-1.00)	0.735	0.07 (-0.15-0.29)	0.548	-23.61 (-54.48-7.27)	0.134
Remittent psychiatric diagnosis	0.39 (-0.35-1.14)	0.298	0.09 (-0.85-1.04)	0.847	-0.01 (-0.23-0.21)	0.928	-2.17 (-7.42-3.07)	0.417
Persistent psychiatric diagnosis	-0.39 (-1.16-0.38)	0.315	-0.42 (-1.40- 0.58)	0.412	0.14 (-0.11-0.39)	0.276	-17.23 (-36.84-2.39)	0.085
SDQ impact score	**0.25 (0.12-0.39)**	**<0.001**	**0.20 (1.19-1.47)**	**0.019**	0.01 (-0.02-0.04)	0.458	-0.34 (-1.51-0.83)	0.569
Test statistics[a1]	AIC 16.97193<br>BIC -353.9633<br>$R^2 = 0.22$		AIC 16.24671<br>BIC -308.0671<br>$R^2 = 0.15$		AIC 20.26365<br>BIC -24.59063<br>$R^2 = 0.79$		AIC 15.83608<br>BIC 1.230216<br>$R^2 = 0.90$	

Notes: Results in bold are significant (p<0.05). Models adjusted by city and method of interview.

[a1] Cameron & Windmeijer's *R*-squared, measure of goodness of fit for the class of exponential family regression models.

Discussion

We analysed data on mental health-related service use and associated costs among a prospective community cohort of young people in Brazil. We found that impact of mental health problems on daily life and parental stigma were the most consistent and robust drivers of mental health service use and associated costs~~, above and beyond persistence of psychiatric disorder~~.

Formatted: Font color: Red

Drivers of mental-health service use costs

The association between disorder impact and mental health-related service use and costs that we found has been observed in previous research, providing further support that impact and impairment tend to be the strongest and most robust predictors of mental health service use [13,33] and costs [14]. Contrary to what we expected, we did not find an association between disorder persistence and costs. Our analyses instead found an that impact of the disorder on adolescent's life was the most important clinical predictor and that this was what seemed to drive service use rather than type or persistence of diagnosis. Nevertheless, it is important to consider that we have estimated annual costs, and these do not necessarily reflect the cumulative economic costs of persistent cases across childhood and adolescence.

A novel result we found was that lower parental stigma was associated with greater service use and higher costs. Our findings suggest that the ways in which parents perceive mental illness in adolescents may significantly influence help-seeking. We are aware of one study which showed that young people's likelihood of service use across health and education settings was greater among caregivers who reported less intended stigmatising behaviours [16] Another study indicated that low parental stigmatising attitudes toward mental disorders increased recognition of mental health problems in preadolescents (10-12 years) [43].

We did not find any study exploring the impact of parental stigmatising attitudes toward mental illness on child treatment costs. Other research -has shown that parental stigma can

impede problem recognition and help-seeking [17,43]. Higher stigma amongst parents and caregivers may discourage or delay service access for their children [16], which may reduce the short-term public sector direct costs of treatment but be detrimental in the long run.

Formatted: Font color: Red

~~Studies among adults suggest that stigma reduces help-seeking (Clement et al., 2015), and increases non-adherence to treatment and early withdrawal from services (Clement et al., 2015; Kamaradova et al., 2016). As these results come from studies conducted with adult populations with psychiatric conditions, we~~Future research needs to further explore the mechanisms through which parental stigma may be related to service/treatment selection and treatment adherence, in order to explain its impact on treatment costs. Moreover, as lower parental stigma may facilitate earlier service contact, it would be interesting to investigate if lower parental stigma may result in lower costs in the longer term.

Among sociodemographic variables, we found that low SEG predicted higher odds of educational service use. This may be related to the fact that young people living in deprived circumstances are more likely to be affected by developmental problems [44], and, therefore, are more likely to use special education services [11]. Although our study did not identify any differences in service use according to ethnicity, we found white ethnicity was associated with higher service use costs. This may reflect disparities in the type of mental health treatment offered or available to non-white children/adolescents. According to previous studies, non-white children/adolescents are less likely to receive adequate mental health treatment [45], including lower likelihood of psychopharmacological prescriptions [46], compared with white children/adolescents.

The economic impact of adolescent mental health care by sectors

We found that the health sector was clearly the main sector ~~providing mental~~accessed by youth with mental disorders ~~health care for youth~~. Within the health sector, specialty mental health care was used more frequently and was more costly than primary care. In Brazil, access to CAPS does not require any referral. However, the number of CAPS services are limited, and they are focused on treatment of severe mental disorders [24]. The high costs incurred by the mental health sector for the treatment of psychiatric disorders in CAPS may be a result of both, the severity of patients consulting these services and because these services provide intensive outpatient treatments (reflected by the highest number of visits we found), which is costly compared with no-specialized services. It is important to highlight that t~~T~~he lack of youth-oriented primary care mental health ~~programmes~~services in Brazil limits access to treatment ~~when symptoms start to have an impact on adolescent functioning~~. This could explain why we found low frequency of mental health-related contacts with GP/ family doctors. As a result, contact with specialist mental health services only happens when the disorder has significant negative impact on the lives of young people. In this sense, the organisation of a mental health network of care for adolescents, integrating primary care, social care, education, criminal justice and community youth-specialist services, according to the impact of cases, must be considered in Brazil to adequately plan and allocate scarce public budgets [47].

Formatted: Font color: Red

We found that mental-health related educational service use was less prevalent compared with health service use, nevertheless –as previous studies have shown– [11,14] educational service use was also associated with higher costs. In Brazil, while special education services are provided in regular schools, their use is restricted to students with disabilities and developmental disorders [48], so only adolescents with severe mental disorders are likely to be eligible.

Limitations

Our study has several limitations. First, the psychometric properties of the adapted version of the SACA have not been evaluated yet. Second, as we were not able to access administrative records, service use assessment was limited to guardians' reports. However, the concordance between parent report and records for service use on the parental version of the SACA is strong [36].

~~Second~~Third, as most of the unit cost were specifically identified for the cities where the HRC is being conducted, São Paulo and Porto Alegre, they are not necessarily generalisable to the whole country.

Fourth~~ Third~~, due to the limited number of participants using each type of service, we were unable to compare factors related with use and associated costs of specific types of service. Furthermore, given our estimates come from observational cohort data, we are not able to establish causality.

Conclusions

Our findings suggest that the main drivers of health-related service use costs among adolescents in Brazil were impact of mental health problems, in addition to lower stigma toward people with mental illness among guardians and White ethnicity~~lower ethnic barriers~~White ~~ethnicity~~. In the present study, only 22.4% of young people with a diagnosed mental disorder received any form of care. In addition to reducing inequality in service use among children, our findings also argue for lowering barriers to care, in particular addressing caregiver stigma. Furthermore, ~~B~~because lower use of services in adolescence may be associated with worse outcomes across the life course [47], it is needed to further explore measures to reduce

inequalities in service utilisation by ~~children~~young people, even though this implies higher short-term costs.

~~Impact of mental health problems on children's lives and guardian's~~ Guardian's lower stigmatising attitudes towards mental disorders may be crucial to support young people in accessing, engaging and maintaining contac~~ts~~ with mental health-related services. ~~In this sense, health and education policies need to better support guardians to access appropriate services in their communities. In addition, various~~ Various anti-stigma interventions have ~~been effectives~~demonstrated effectiveness for improving help-seeking [49], but few have been implemented in LMICs. Further studies are needed to design and implement anti-stigma interventions in LMICs. On the other hand,~~In this sense,~~ health and education policies need to better support guardians to access appropriate and timely services in their communities, before the symptoms have a significant impact on adolescent functioning.~~ In~~ ~~Finally,~~We conclude that the organisation of a culturally sensitive mental health network of care for adolescents, integrating primary care, social care, education, criminal justice services and ~~community youth specialist services~~CAPS, ~~according to the impact of cases,~~ must be considered in Brazil to adequately plan and allocate scarce public budgets ~~(Knapp et al., 2016).~~

Formatted: Font color: Red

Formatted: Font color: Red

Formatted: Font color: Red

Formatted: Font color: Red

Financial Support: The research presented in this article was funded by the European Research Council under the European Union's Seventh Framework Programme (FP7/2007-2013)/ERC grant agreement no 337673, and supported by the National Institute of Developmental Psychiatry for Children and Adolescents, a science and technology institute funded by Conselho Nacional de Desenvolvimento Científico e Tecnológico (CNPq; National Council for Scientific and Technological Development; grant numbers 573974/2008-0 and 465550/2014-2) and Fundação de Amparo à Pesquisa do Estado de São Paulo (FAPESP grant number 2008/57896-8 and 2014/50917-0). SEL receives support from the UK Medical Research Council in relation

to the Mentalkit-Brazil project (MR/R022763/1) and the Economic and Social Research Council. CZ received a doctoral scholarship and research abroad scholarship by the Fundação de Amparo à Pesquisa do Estado de São Paulo (grant number 2018/05586-7 and 2019/08731-0). MSH was supported by the Newton International Fellowship (Ref: NIF\R1\181942), awarded by the Academy of Medical Sciences through the UK Government's Newton Fund Programme. The funding organisations had no role in the study design; collection, analysis or interpretation of data; in the writing of this article; or in the decision to submit the article for publication.

Conflicts of interests: All authors report no conflict of interest associated with this publication. Luis Augusto Rohde has received grant or research support from, served as a consultant to, and served on the speakers' bureau of Aché, Bial, Medice, Novartis/Sandoz, Pfizer/Upjohn, and Shire/Takeda in the last three years. The ADHD and Juvenile Bipolar Disorder Outpatient Programs chaired by Dr Rohde have received unrestricted educational and research support from the following pharmaceutical companies in the last three years: Novartis/Sandoz and Shire/Takeda. Dr Rohde has received authorship royalties from Oxford Press and ArtMed. None of these commercial relationships alter our adherence to PLOS ONE policies on sharing data and materials.

~~The authors~~ **~~CZ, WR, DK, DMD, MH, RR, GS, PP, EM, JM, and SEL have no conflicts of interest to declare. LR has received grant or research support from, served as a consultant to, and served on the speakers' bureau of Medice, Novartis/Sandoz and Shire/Takeda in the last three years. The ADHD and Juvenile Bipolar Disorder Outpatient Programs chaired by LR have received unrestricted educational and research support from the following pharmaceutical companies in the last three years: Novartis/Sandoz and Shire/Takeda. LR has received authorship royalties from Oxford Press and ArtMed and~~**

Formatted: Font: Not Bold

Formatted: Font: Bold, Font color: Red

~~travel grants from Shire to take part in the 2018 APA annual meeting and from Novartis to take part of the 2017 AACAP annual meeting. RB: Pfizer, Torrent and Sanofi-Aventis.~~

Access to data and Data sharing: CZ have full access to all the data used in the study and takes responsibility for the integrity of the data and the accuracy of the data analysis. Data were provided by the Brazilian High-Risk Cohort study and are available upon request in the Open Science Framework public repository (https://osf.io/ktz5h/).

REFERENCES

1. Polanczyk G V., Salum GA, Sugaya LS, Caye A, Rohde LA. Annual Research Review: A meta-analysis of the worldwide prevalence of mental disorders in children and adolescents. J Child Psychol Psychiatry. 2015;56: 345–365. doi:10.1111/jcpp.12381
2. Knapp M, King D, Healey A, Thomas C. Economic outcomes in adulthood and their associations with antisocial conduct, attention deficit and anxiety problems in childhood. J Ment Health Policy Econ. 2011;14: 137–47.
3. Kim-Cohen J, Caspi A, Moffitt TE, Harrington H, Milne BJ, Poulton R. Prior juvenile diagnoses in adults with mental disorder: developmental follow-back of a prospective-longitudinal cohort. Arch Gen Psychiatry. 2003;60: 709–17. doi:10.1001/archpsyc.60.7.709
4. Richards, M; Abbott R. Childhood mental health and life chances in post-war Britain. Insights from three national birth cohort studies. London: The Smith Institute, Sainsbury Centre for Mental Health & Unison; 2009.
5. Chen H, Cohen P, Kasen S, Johnson JG, Berenson K, Gordon K. Impact of Adolescent Mental Disorders and Physical Illnesses on Quality of Life 17 Years Later. Arch Pediatr Adolesc Med. 2006;160: 93. doi:10.1001/archpedi.160.1.93
6. Zechmeister I, Kilian R, McDaid D. Is it worth investing in mental health promotion and prevention of mental illness? A systematic review of the evidence from economic evaluations. BMC Public Health. 2008;8: 20. doi:10.1186/1471-2458-8-20
7. McCrone P, Knapp M, Fombonne E. The Maudsley long-term follow-up of child and adolescent depression. Eur Child Adolesc Psychiatry. 2005;14: 407–413. doi:10.1007/s00787-005-0491-6
8. Knapp M; Evans-Lacko S. Health Economics. 6th ed. In: Thapar A, Pine D, editors. Rutter's Child and Adolescent Psychiatry. 6th ed. Wiley-Blackwell; 2015. pp. 227–238.
9. Knapp M, Wong G. Economics and mental health: the current scenario. World Psychiatry. 2020;19: 3–14. doi:10.1002/wps.20692
10. Beecham J. Annual Research Review: Child and adolescent mental health

interventions: a review of progress in economic studies across different disorders. J Child Psychol Psychiatry. 2014;55: 714–732. doi:10.1111/jcpp.12216

11. Snell T, Knapp M, Healey A, Guglani S, Evans-Lacko S, Fernandez JL, et al. Economic impact of childhood psychiatric disorder on public sector services in Britain: Estimates from national survey data. J Child Psychol Psychiatry. 2013; 1–9. doi:10.1111/jcpp.12055

12. Funk M, Saraceno B, Lund C, Flisher AJ, Green A, Knapp M, et al. Planning and budgeting to deliver services for mental health. Mental health policy and service guidance package. Geneva: World Health Organization; 2003.

13. Evans-Lacko S, DosReis S, Kastelic E, Riley A. Mental Health Service Use Before and After Diagnosis of Early-Onset Bipolar Disorder. J Behav Health Serv Res. 2011;38: 398–413. doi:10.1007/s11414-010-9225-8

14. Knapp M, Snell T, Healey A, Guglani S, Evans-Lacko S, Fernandez J-L, et al. How do child and adolescent mental health problems influence public sector costs? Interindividual variations in a nationally representative British sample. J Child Psychol Psychiatry. 2015;56: 667–676. doi:10.1111/jcpp.12327

15. Beecham J, Green J, Jacobs B, Dunn G. Cost variation in child and adolescent psychiatric inpatient treatment. Eur Child Adolesc Psychiatry. 2009;18: 535–542. doi:10.1007/s00787-009-0008-9

16. Gronholm PC, Ford T, Roberts RE, Thornicroft G, Laurens KR, Evans-Lacko S. Mental Health Service Use by Young People: The Role of Caregiver Characteristics. PLoS One. 2015;10: e0120004. doi:10.1371/journal.pone.0120004

17. Clement S, Schauman O, Graham T, Maggioni F, Evans-Lacko S, Bezborodovs N, et al. What is the impact of mental health-related stigma on help-seeking? A systematic review of quantitative and qualitative studies. Psychol Med. 2015;45: 11–27. doi:10.1017/S0033291714000129

18. Kamaradova D, Latalova K, Prasko J, Kubinek R, Vrbova K, Krnacova B, et al. Connection between self-stigma, adherence to treatment, and discontinuation of medication. Patient Prefer Adherence. 2016;10: 1289–1298. doi:10.2147/PPA.S99136

19. Horton S, Toledo ED la C, Mahon J, Santelli J, Waldfogel J. Identifying an Essential Package for Adolescent Health: Economic Analysis. Child and Adolescent Health and Development. Washington, D.C.: The World Bank; 2017. doi:10.1596/978-1-4648-0423-6_ch26

20. Fatori D, Salum G, Itria A, Pan P, Alvarenga P, Rohde LA, et al. The economic impact of subthreshold and clinical childhood mental disorders. J Ment Heal. 2018;27: 588–594. doi:10.1080/09638237.2018.1466041

21. Paula CS, Bordin IAS, Mari JJ, Velasque L, Rohde LA, Coutinho ESF. The Mental Health Care Gap among Children and Adolescents: Data from an Epidemiological Survey from Four Brazilian Regions. PLoS One. 2014;9: e88241. doi:10.1371/journal.pone.0088241

22. Ministério da Educação. Notas Estatísticas Censo Escolar 2018. 2019 [cited 20 Sep

2019]. Available: http://download.inep.gov.br/educacao_basica/censo_escolar/notas_estatisticas/2018/notas_estatisticas_censo_escolar_2018.pdf

23. Agência Nacional de Saúde Suplementar. Beneficiários de planos privados de saúde, por cobertura assistencial (Brasil – 2010-2020). 2020 [cited 15 Apr 2020]. Available: http://www.ans.gov.br/index.php/aans/quem-somos/227-dados-gerais

24. Paula CS, Lauridsen-Ribeiro E, Wissow L, Bordin I a. S, Evans-Lacko S. How to improve the mental health care of children and adolescents in Brazil: Actions needed in the public sector. Rev Bras Psiquiatr. 2012;34: 334–341. doi:10.1016/j.rbp.2012.04.001

25. Salum GA, Gadelha A, Pan PM, Moriyama TS, Graeff-Martins AS, Tamanaha AC, et al. High risk cohort study for psychiatric disorders in childhood: rationale, design, methods and preliminary results. Int J Methods Psychiatr Res. 2015;24: 58–73. doi:10.1002/mpr.1459

26. Weissman MM, Wickramaratne P, Adams P, Wolk S, Verdeli H, Olfson M. Brief Screening for Family Psychiatric History: The Family History Screen. Arch Gen Psychiatry. 2000;57: 675–682. doi:10-1001/pubs.Arch Gen Psychiatry-ISSN-0003-990x-57-7-yoa8214

27. Associação Brasileira de Empresas de Pesquisa. Critério de Classificação Econômica Brasil. 2018 [cited 15 Apr 2020]. Available: http://www.abep.org/criterio-brasil

28. Goodman R, Ford T, Richards H, Gatward R, Meltzer H. The Development and Well-Being Assessment: description and initial validation of an integrated assessment of child and adolescent psychopathology. J Child Psychol Psychiatry. 2000;41: 645–55.

29. Fleitlich-Bilyk B, Goodman R. Prevalence of Child and Adolescent Psychiatric Disorders in Southeast Brazil. J Am Acad Child Adolesc Psychiatry. 2004;43: 727–734. doi:10.1097/01.chi.0000120021.14101.ca

30. Sourander A, Helstelä L, Helenius H. Parent-adolescent agreement on emotional and behavioral problems. Soc Psychiatry Psychiatr Epidemiol. 1999;34: 657–663. doi:10.1007/s001270050189

31. Salbach-Andrae H, Klinkowski N, Lenz K, Lehmkuhl U. Agreement between youth-reported and parent-reported psychopathology in a referred sample. Eur Child Adolesc Psychiatry. 2009;18: 136–143. doi:10.1007/s00787-008-0710-z

32. Martel MM, Pan PM, Hoffmann MS, Gadelha A, do Rosário MC, Mari JJ, et al. A general psychopathology factor (P factor) in children: Structural model analysis and external validation through familial risk and child global executive function. J Abnorm Psychol. 2017;126: 137–148. doi:10.1037/abn0000205

33. Stringaris A, Goodman R. The Value of Measuring Impact Alongside Symptoms in Children and Adolescents: A Longitudinal Assessment in a Community Sample. J Abnorm Child Psychol. 2013;41: 1109–1120. doi:10.1007/s10802-013-9744-x

34. Evans-Lacko S, Rose D, Little K, Flach C, Rhydderch D, Henderson C, et al. Development and psychometric properties of the Reported and Intended Behaviour Scale (RIBS): a stigma-related behaviour measure. Epidemiol Psychiatr Sci. 2011;20:

263–271. doi:10.1017/S2045796011000308

35. Ribeiro WS, Gronholm PC, Silvestre de Paula C, Hoffmann MS, Rojas Vistorte AO, Zugman C, et al. Development and validation of the Brazilian Portuguese Version of the Reported and Intended Behaviour Scale (RIBS-BP). Stigma Heal. 2020;In press. doi:10.1037/sah0000224

36. Hoagwood K, Horwitz S, Stiffman A, Weisz J, Bean D, Rae D, et al. Concordance Between Parent Reports of Children's Mental Health Services and Service Records: The Services Assessment for Children and Adolescents (SACA). J Child Fam Stud. 2000;9: 315–331. doi:10.1023/A:1026492423273

37. Horwitz SM, Hoagwood K, Stiffman AR, Summerfeld T, Weisz JR, Costello EJ, et al. Reliability of the Services Assessment for Children and Adolescents. Psychiatr Serv. 2001;52: 1088–1094.

38. Banco Central do Brasil. Conversor de moedas. 2018 [cited 10 Oct 2019]. Available: https://www.bcb.gov.br/conversao

39. Razzouk D. Accommodation and Health Costs of Deinstitutionalized People with Mental Illness Living in Residential Services in Brazil. PharmacoEconomics - Open. 2019;3: 31–42. doi:10.1007/s41669-018-0078-z

40. Becker P, Razzouk D. Cost of a community mental health service: a retrospective study on a psychosocial care center for alcohol and drug users in São Paulo. Sao Paulo Med J. 2018;136: 433–441. doi:10.1590/1516-3180.2018.0164310818

41. Banco Central do Brasil. Calculadora do Ciudadão. Correção de valores. 2018 [cited 10 Sep 2019]. Available: https://www3.bcb.gov.br/CALCIDADAO

42. Mihaylova B, Briggs A, O'Hagan A, Thompson SG. Review of statistical methods for analysing healthcare resources and costs. Health Econ. 2011;20: 897–916. doi:10.1002/hec.1653

43. Villatoro AP, DuPont-Reyes MJ, Phelan JC, Painter K, Link BG. Parental recognition of preadolescent mental health problems: Does stigma matter? Soc Sci Med. 2018;216: 88–96. doi:10.1016/j.socscimed.2018.09.040

44. Blair C, Raver CC. Poverty, Stress, and Brain Development: New Directions for Prevention and Intervention. Acad Pediatr. 2016;16: S30–S36. doi:10.1016/j.acap.2016.01.010

45. Alegria M, Vallas M, Pumariega AJ. Racial and Ethnic Disparities in Pediatric Mental Health. Child Adolesc Psychiatr Clin N Am. 2010;19: 759–774. doi:10.1016/j.chc.2010.07.001

46. Leslie LK, Weckerly J, Landsverk J, Hough RL, Hurlburt MS, Wood PA. Racial/Ethnic Differences in the Use of Psychotropic Medication in High-Risk Children and Adolescents. J Am Acad Child Adolesc Psychiatry. 2003;42: 1433–1442. doi:10.1097/00004583-200312000-00010

47. Knapp M, Ardino V, Brimblecombe N, Evans-Lacko S, Lemmi V, King D, et al. Youth mental health: new economic evidence. London: Personal Social Services Research Unit; 2016.

48. Ministério da Educação. Decreto Nº 7.611. 2011 [cited 20 Sep 2019]. Available: http://www.planalto.gov.br/ccivil_03/_Ato2011-2014/2011/Decreto/D7611.htm#art11

49. Thornicroft G, Mehta N, Clement S, Evans-Lacko S, Doherty M, Rose D, et al. Evidence for effective interventions to reduce mental-health-related stigma and discrimination. Lancet. 2016;387: 1123–1132. doi:10.1016/S0140-6736(15)00298-6

Supporting information

S1 Fig. Flow chart of Brazilian High-Risk Cohort participants included in the mental-health related service use study.

S1 Table. Unit costs of health, educational, social care and criminal justice related services.

S2 Table. Bivariate analysis: Predictors of 12-month mental health service utilisation.

S3 Table. Bivariate analysis: Predictors of cost of mental health service use in the last 12 months.

S4 Table. Logistic regression models: 12-month mental health service utilisation predicted by psychiatric diagnosis trajectories.

S5 Table. Logistic regression models: 12-month mental health service utilisation predicted by impact of behavioural and emotional difficulties on child' life.

S6 Table. Logistic regression models: 12-month mental health service utilisation predicted by parental stigma.

S7 Table. Generalised linear models: cost of 12-month mental health service utilization predicted by psychiatric diagnosis trajectories.

S8 Table. Generalised linear models: cost of 12-month mental health service utilisation predicted by impact of behavioural and emotional difficulties on child' life.

S9 Table. Generalised linear models: cost of 12-month mental health service utilisation predicted by parental stigma.

S10 Table. Generalised linear models: cost of 12-month mental health service utilisation predicted by broad diagnosis categories.

January 27th, 2022

Manuscript number PONE-D-21-10663
Manuscript Title: Utilisation and costs of mental health-related service use among adolescents

We appreciate the careful revision of our manuscript and the comments of the reviewers. We are pleased to be invited to submit the revised version of our paper to PLOS ONE.

Please find attached both an unmarked version of the revised manuscript and one version with changes marked in red. Our point-by-point responses to the reviewers' comments (unquoted italics) and details of the changes we have performed to our revised manuscript are given below.

Reviewer #1:
General Comment: *Very relevant and interesting study. Well written paper, I found it pleasant to read. I would recommend some minor adjustments*
Response: We appreciate your positive feedback, the careful revision of our manuscript and your comments.

Comment 1: *Abstract- When only reading the abstract, the distinction between incident, remittent and persistent disorder in the Results section is a bit confusing. For the abstract, I would recommend rewriting this sentence for example: "Higher odds of service use were associated with having a diagnosed mental disorder (either incident, remittent or persistent), higher impact of symptoms etc."*
Response: Thank you for your comment. We have rewritten this sentence as follows: Higher odds of service use were associated with having a diagnosed mental disorder (either incident [OR=2.49, 95%CI=1.44-4.30, p=0.001], remittent [OR=2.16, 95%CI=1.27-3.69, p=0.005] or persistent [OR=3.01, 95%CI=1.69-5.36, p<0.001]), higher impact of symptoms..

Comment 2: *Introduction- This study focuses on the economic cost of mental disorders in young people (line 52). Therefore, it should be better introduced why, in addition to (mental) health services, also education, criminal justice and social care services were investigated.*
Response: We have edited the introduction as follows:
The high prevalence and potentially enduring nature of these impacts make addressing youth mental health conditions particularly important, but this is a challenge for public systems with limited resources (Knapp M; Evans-Lacko S, 2015). Economic costs associated with youth mental health conditions involve a wide range of sectors including health, educational, social care, and criminal justice services [9,10]. This can

represent a substantial cost to the public purse, yet it could also be considered a wise investment given the evidence that effective treatment can mitigate the impact of poor mental health (Knapp et al., 2011).

Comment 3: *Line 55: male gender is mostly not associated with higher use of mental health services. Please specify the association between these factors and specific services.*

Response: We appreciate your suggestion. We have edited this paragraph in the revised version of the manuscript:

Some studies from high–income countries suggest that lower socioeconomic status, as well as clinical features (illness severity and impact of disorders) are associated with use of health, special education, and social care services, while male gender and older age are associated with more criminal justice services contacts [11,13,14]. These sociodemographic and clinical characteristics are also associated with greater mental health-related treatment costs among young people [11,14,15]

Comment 4: *Methods: Data and participants. I understand that not all information about the Brazilian High-Risk Cohort was included in this paper. I would want to know, however, based on what information the children became part of this high risk cohort. Are they COPMI?*

Response: Thanks for the important point you raised. We have added information in the methods on the Brazilian High-Risk cohort sampling procedures as follows:

This study is nested within the Brazilian High-Risk Cohort (BHRC), which is an ongoing prospective longitudinal study that comprises a community sample and a high-risk sub-sample (a sample at increased risk of mental disorders) of young people from Sao Paulo and Porto Alegre, Brazil. A detailed description of the sample and procedures can be found elsewhere [25]. Briefly, during the registry day, 12,500 parents of young people aged 6 to 14 years attending 57 schools (22 in Porto Alegre and 35 in São Paulo) were invited to a screening of mental health disorders using the Family History Screen (FHS) [26]. A total of 8,012 families (9,937 eligible children, 45,394 family members) were interviewed. Based on the percentage of members in the family that screened positively for psychiatric disorders, an index of family load for each potential eligible child was computed. The final cohort comprised 2,511 young people; 957 were randomly selected, and 1,554 were a sub-sample at increased risk of mental disorders based on the FHS.

Comment 5: *Methods: Measures. Why only maternal educational level?*

- Furthermore, this paragraph forms a clear description of appropriate measures.

Response: As stated in the methods section, the socioeconomic group variable comprised head of household educational level in addition to other household socioeconomic indicators. As some research suggests that mothers educational level is particularly important for recognition and help-seeking, we also included this variable as a separate indicator. As the vast majority of caregiver respondents were mothers (in 93% of cases the biological mother [information included in the revised manuscript])

we focused on maternal education rather than estimating the educational level of other caregivers.

Comment 6: *Results. Very clear description and informative tables.*
Response: Thank you very much for your positive feedback.

Comment 7: *Discussion- Line 325: "We found that the health sector was clearly the main sector providing mental health care for youth." That's quite obvious. I would recommend rewriting this, for example: "We found that the health sector was clearly the main sector accessed by youth with mental disorders."*
Response: Thank you very much for your suggestion. We rewrote this sentence as follows:
We found that the health sector was clearly the main sector accessed by youth with mental disorders.

Comment 8: *In the present study, only 20% of young people with a diagnosed mental disorder received any form of care. In addition to reducing inequality in service use among children, these data also argue for lowering barriers to care for young people in general. I would recommend stating this in the conclusion as well.*
Response: Thanks for your suggestion. We have edited the first paragraph of the conclusions as follows:

Our findings suggest that the main drivers of health-related service use costs among adolescents in Brazil were impact of mental health problems, in addition to lower stigma toward people with mental illness among guardians and White ethnicity. In the present study, only 22.4% of young people with a diagnosed mental disorder received any form of care. In addition to reducing inequality in service use among children, our findings also argue for lowering barriers to care, in particular addressing caregiver stigma. Furthermore, because lower use of services in adolescence may be associated with worse outcomes across the life course [47], it is needed to further explore measures to reduce inequalities in service utilisation by young people, even though this implies higher short-term costs.

Comment 9: *Line 329: "The lack of youth-oriented primary care mental health programmes". Is this also the reason why GP's/family doctors were less frequently visited*?
Response: We appreciate your comment, and we agree with your interpretation of this result. We have edited the referred sentence:
The lack of youth-oriented primary care mental health programmes limits access to treatment when symptoms start to have an impact on adolescent functioning. This can explain why we found a low rate of mental health-related contacts with GP/ family doctors. As a result, contact with specialist mental health services only happens when the disorder has significant negative impact on the lives of young people.

Comment 10: *Line 359-361: this reads like the impact of mental health problems on*

children's lives should be increased because it would support help-seeking. Please, rewrite.
Response: We have rewritten this paragraph:
Guardian's lower stigmatising attitudes towards mental disorders may be crucial to support young people in accessing, engaging and maintaining contact with mental health-related services. Various anti-stigma interventions have demonstrated effectiveness for improving help-seeking [49], but few have been implemented in LMICs. Further studies are needed to design and implement anti-stigma interventions in LMICs. On the other hand, health and education policies need to better support guardians to access appropriate and timely services in their communities, before the symptoms have a significant impact on adolescent functioning.

Comment 11: *Line 363: effectives should be effective*
Response: Thank you very much, we have corrected this error.

Comment 12: *In future research, it would be interesting to not only assess parental stigma but also stigma among the adolescents themselves.*
Response: We agree with you, and we are planning to evaluate the association between mental health-related service use and youth stigma towards mental illness in future cohort's assessments.

Reviewer #2:

General comment: *It's good to see more representative research from LMICs, trying to bridge the existing knowledge gap. This study's most significant plus point is that it looks at service use and service cost from multiple angles, shedding light on demographic, clinical and systemic factors that contribute to service use cost. However, this manuscript does require significant improvement in language and content. Here are my main suggestions:*
Response: We appreciate your positive opinion of our work, the careful revision of our manuscript and your valuable comments.

Comment 1: *The language of the manuscript can be crisper. Multiple places sentences look disjointed or elongated. The paragraphs are changed too frequently in some places, with each of these paragraphs containing only one or two sentences.*
Response: Thanks for your comment. We have revised and edited the language through the manuscript.

Comment 2: *Introduction: In line 57, please clarify whether by 'education services' authors mean remedial education services or some other kind of services?*
Response: Thanks for your comment. We have indicated 'special education' in the

revised version of the manuscript.

Comment 3: *Introduction: The lines 55-58 are difficult to follow: authors claim that certain demographic and clinical characteristics are associated with a greater likelihood of using certain services as per existing research. However, it's not clear how this connects with the assertion about young people in the same sentence.*
Response: Thanks for your comment. We have edited and separated these sentences: Some studies from high–income countries suggest that lower socioeconomic status, as well as clinical features (illness severity and impact of disorders) are associated with use of health, special education, and social care services, while male gender and older age are associated with more criminal justice service contacts [11,13,14]. These sociodemographic and clinical characteristics are also associated with greater mental health-related treatment costs among young people [11,14,15].

Comment 4: *Introduction: The importance of studying parental stigma needs to be built better.*
Response: We appreciate your suggestion. We have included the following changes: Families also play a central role in young people's contact with services. One study from the UK found that lower mental illness-related stigma among caregivers was associated with an increased likelihood of young people's mental health service use [16]. Stigmatising attitudes toward mental illness amongst parents may influence service contacts due to shame and fears of labelling their child's mental health condition [16]. There are clear links between stigma and reduced help-seeking [17], reduced adherence to treatment and early withdrawal from services [17,18]. However, little is known about/ how parental stigma could impact on young people service use and costs.

Comment 5: *Introduction: I'm not sure what is meant by 'beyond diagnosis', are authors implying the existing studies cover the cost of diagnosis only or for limited kinds of disorders. Some clarification here would be helpful.*
Response: Thanks for your suggestion. We have edited this sentence as follows: Additionally, little is known about how, in addition to the type of disorder, whether persistence of psychopathology from childhood to adolescence, disorders' impact on adolescent's daily life (i.e., functioning), and key barriers to care such as stigma, could influence costs.

Comment 6: *Introduction: The way lines 72-73 are written makes it sound like Brazil is a high-income country*
Response: We appreciate your comment. We have deleted 'Similar to most high income countries' in the revised version of the manuscript.

Comment 7: *Introduction: In line 88, it's unclear what characteristics the authors are referring to and whether the following hypothesis is related to a subset of these characteristics?*
Response: We have rewritten this sentence to clarify the characteristics under study:

Second, we examine how costs vary according to: mental health trajectories, impact of the disorder on everyday life, and parent/guardian stigma towards mental illness.

Comment 8: *Methods: In line 96, some information on how these children were classified as high risk will be helpful. The authors have said the details are somewhere else, but a brief description here will make it easier for the reader to understand the sample.*
Response: Thanks for your suggestion. As explained in response to Reviewer 1's comment 4, we have included a brief description of the Brazilian High-Risk Cohort sampling procedures.

Comment 9: *Methods: In line 99, it was slightly hard to follow study timelines. Was this study carried out after the first follow-up in 2014-2015 or as part of the follow-up?*
Response: We have tried to clarify this including the following information:
Cohort participants were interviewed at baseline (aged 6-14 years, calendar year:2010-2011, n=2,511), and at first follow-up (N=2010, aged 9-17 years, calendar year 2014). After completing the BHRC first follow-up interview, 1,881 parents/guardians were invited to respond to a supplementary interview which included a comprehensive assessment of mental health related service use (calendar year: 2014-2015, young people participants aged 10-18 years).

Comment 10: *Methods: The authors can use consistent terminology: children or young people. As of now, this has varied from one sentence to another.*
Response: Thanks for your comment. We have revised and edited the methods section in order to use consistently the term young people.

Comment 11: *The '-' in line 102 seems typo.*
Response: We appreciate your comment. We have deleted this typo.

Comment 12: *Methods: In lines 127-130, it's unclear why young people were not interviewed at baseline but were included during the 3-year follow-up?*
Response: This was because participants were younger at baseline and so we relied on parent's report, given limitations in funding and resources. Given that older adolescents are better at reporting internalising symptoms, both guardian and youth interviews were performed at 3-year follow-up. We included this explanation in the revised version of the manuscript:
At baseline, diagnostic assessment and interviews were performed with guardians only. Previous literature has found that self-reports on internalising conditions during adolescence is higher compared with parental report. This can be explained because internalising problems, such as anxiety or depression, would be less observable by guardians, being advisable to consider both reports to reach a reliable evaluation of adolescent mental health [30,31]. For this reason, diagnostic assessment at 3-year follow-up was performed considering guardian reports and additional information from interviews with the young people about internalising conditions.

Comment 13: *Methods: Do authors have any psychometric properties of the adapted version of Service Assessment for Children and Adolescents that can be reported in this publication?*

Response:

The parent-report SACA has been shown to be a valid measure of young people's service use (kappa = 0.76; [Hoagwood et al., 2000]) with test-retest reliability for past-year reports (ranging from 0.75 to 0.86; [Horwitz et al., 2001]). We have not assessed the psychometric properties of the adapted version of the Service Assessment for Children and Adolescents for Brazilian participants yet. We have included this limitation in the revised version of the manuscript.

Comment 14: *Results: In line 224, the authors refer to Table 1. However, without any commentary on the significance of data in this table, the authors jump to a new set of findings. All this makes it slightly hard to follow what is being presented.*

Response: We appreciated your comment. We have edited this paragraph:

Table 1 describes sociodemographic and clinical characteristics of participants. The sample comprised 1,400 adolescents with a mean age of 14 years (s.d=1.98). The majority were white males from low SEG, and only 10% of mothers had university education. 23.3% (n= 326) of adolescents had a psychiatric disorder in the previous 12 months, of which 177 (54.3%) were incident and 149 (45.7%) persistent cases since baseline. 213 (15.2%) participants had remitted from a baseline psychiatric diagnosis. Participants with externalising disorders were more likely to have persistent trajectories (RR=2.19, 95%CI=1.38-3.48, $p<0.001$). Participants categorised as persistent also reported greater disorder impact (β=2.34, 95%CI=2.11-2.58, $p<0.001$). 22.4% of those who presented with a psychiatric disorder reported using some type of service for their mental health in the previous twelve months. The proportion of service use among those who presented a persistent psychiatric condition was 27%. Table 1 also describes the mean costs of mental health-related service use in the past year, by psychiatric trajectory (from no diagnosis to persistent psychiatric diagnosis). Bivariate analyses showed a non-significant association between psychiatric trajectory and mean annual costs.

Comment 15: *The 12-month service use and service use cost means are presented in Tables 1 and 2. Repeating the same findings across two tables should be avoided*

Response: We have deleted the last line of Table 2 (overall services cost).

Comment 16: *The paragraph on page 12 lacks a description of the cost associated with each service? For e.g., although CAPS is not a highly prevalent service, the associated cost makes for a lion contribution to the public purse. This needs to be presented and discussed.*

Response: Thank you very much for your suggestion. We edited this paragraph as follows:

Utilisation of mental health services in the previous 12 months and associated cost by type of service are presented in Table 2. Overall, 10.0% of the sample (n=143)

used some sort of health, education, criminal justice or social care service for mental health problems. Disaggregating by sectors, the health sector had highest proportion of service users (9%), while the education and social care and criminal justice sectors were less frequently contacted with a 1.8% and 1.3% of users, respectively. Outpatient mental health services, most notably psychologists and psychiatrists in settings other than community mental health clinics, were the most frequently used services/professionals. Inpatient services and general health services such as GP/family doctor, paediatrician and emergency department, were less frequently used.

In the education sector, school assistant was the most type of service used by young people, while guardianship council was the most frequently social care service contacted. The total cost of 12-month mental health-related service use for the public purse was 70,110.23 USD. The sector that presented higher total annual cost was the health sector, followed by the education and finally the social care and criminal justice sectors. The services that generated the greatest total costs for the heath sector were psychologist (11,339.64 USD) and CAPS (9,628.01 USD). Among those who used services, the average annual cost of service use amounted to 527.14 USD (SD= 908.10 USD, range=8.77- 7,605.58 USD, median=221.10 USD, interquartile range=545.28) per user. Individuals using CAPS (specialty mental health) services (1.1% of the sample) had the highest mean number of visits during the previous year and the highest associated costs among health services. The second highest mean costs in the health sector were related to hospitalizations in psychiatric hospitals and alcohol and drugs clinics, while the lowest mean costs were attributed to emergency department, paediatrician, outpatient alcohol and drugs and GP/family doctor contacts. Although only 0.1% of individuals used shelters, this type of social service had the highest associated mean cost. Education services were used by 1.8% of individuals and these services had the second highest associated mean costs.

Comment 17: *Discussion: In line 288, the use of the terms 'above and beyond' doesn't convey much. To the best of my knowledge, the current analysis nowhere helps to reach this conclusion of above and beyond. I am requesting authors to look at terminology closely.*
Response: Thanks for your suggestion. We have removed this language.
We found that impact of mental health problems on daily life and parental stigma were the most consistent and robust drivers of mental health service use and associated costs..

Comment 18: *Discussion: Lines 301-312 can be streamlined and better organised.*
Response: We have edited the cited lines as follows:

We did not find any study exploring the impact of parental stigmatising attitudes toward mental illness on child treatment costs. Other research has shown that parental stigma can impede problem recognition and help-seeking [17,43]. Higher stigma amongst parents and caregivers may discourage or delay service access for their children [16], which may reduce the short-term public sector direct costs of treatment but be detrimental in the long run. Future research needs to further explore the mechanisms through which parental stigma may be related to service/treatment

selection and treatment adherence, in order to explain its impact on treatment costs. Moreover, as lower parental stigma may facilitate earlier service contact, it would be interesting to investigate if lower parental stigma may result in lower costs in the longer term.

Comment 19: *Discussion: Line 327: The number of CAPS users was less, but the number of visits and costs for those who used it were very high. These were not reflected in the discussion, nor were its implication for the restructuring health system.*
Response: We appreciate your comment. We have edited the discussion as suggested: In Brazil, access to CAPS does not require any referral. However, the number of CAPS services are limited, and they are focused on treatment of severe mental disorders [24]. The high costs incurred by the mental health sector for the treatment of psychiatric disorders in CAPS may be a result of both, the severity of patients consulting these services and because these services provide intensive outpatient treatments (reflected by the highest number of visits we found), which is costly compared with no-specialized services. It is important to highlight that the lack of youth-oriented primary care mental health services in Brazil which limits access to treatment. This could explain why we found low frequency of mental health-related contacts with GP/ family doctors. As a result, contact with specialist mental health services only happens when the disorder has significant negative impact on the lives of young people. (Moved from the conclusion as suggested in your last comment). In this sense, the organisation of a mental health network of care for adolescents, integrating primary care, social care, education, criminal justice and community youth-specialist services, according to the impact of cases, must be considered in Brazil to adequately plan and allocate scarce public budgets [47].

Comment 20: *Discussion: The hypothesis stated that researchers were interested in examining the impact of persistence of psychiatric disorders from childhood to adolescence on service costs; however, the discussion did not give much attention to this part.*
Response: Thanks for rising this important comment. We have included the following paragraph:
Contrary to what we expected, we did not find an association between disorder persistence and costs. Our analyses instead found that impact of the disorder on adolescent's life was the most important clinical predictor and that this was what seemed to drive service use rather than type or persistence of diagnosis. Nevertheless, it is important to consider that we have estimated annual costs, and these do not necessarily reflect the cumulative economic costs of persistent cases across childhood and adolescence.

Comment 21: *Conclusion: Some of the text in the last paragraph of the conclusion, i.e. those referring to implications, can be moved to discussion and expanded further.*
I'm not able to comment on cost analysis as this is not my area of expertise.

Response: Thanks for your suggestions, we have moved some conclusions to the discussion as explained in response to your Comment #20.
