# Supplementary material for: Utilisation and costs of mental health-related service use among adolescents
Source: PLoS One. 2022 Sep 9;17(9):e0273628. doi: 10.1371/journal.pone.0273628 (PMC9462733; doi:10.1371/journal.pone.0273628)
Supplement: Attachment — Submitted filename: Response to Reviewers.docx [file pone.0273628.s012.docx]

January 27th, 2022

**Manuscript number PONE-D-21-10663**

**Manuscript Title: Utilisation and costs of mental health-related service use among adolescents**

We appreciate the careful revision of our manuscript and the comments of the reviewers. We are pleased to be invited to submit the revised version of our paper to PLOS ONE.

Please find attached both an unmarked version of the revised manuscript and one version with changes marked in red. Our point-by-point responses to the reviewers’ comments (unquoted italics) and details of the changes we have performed to our revised manuscript are given below.

**Reviewer #1:**

**General Comment:** *Very relevant and interesting study. Well written paper, I found it pleasant to read. I would recommend some minor adjustments*

**Response:** We appreciate your positive feedback, the careful revision of our manuscript and your comments.

**Comment 1:** *Abstract- When only reading the abstract, the distinction between incident, remittent and persistent disorder in the Results section is a bit confusing. For the abstract, I would recommend rewriting this sentence for example: “Higher odds of service use were associated with having a diagnosed mental disorder (either incident, remittent or persistent), higher impact of symptoms etc.”*
**Response:** Thank you for your comment. We have rewritten this sentence as follows:

Higher odds of service use were associated with having a diagnosed mental disorder (either incident [OR=2.49, 95%CI=1.44-4.30, p=0.001], remittent [OR=2.16, 95%CI=1.27-3.69, p=0.005] or persistent [OR=3.01, 95%CI=1.69-5.36, p<0.001]), higher impact of symptoms..

**Comment 2:** *Introduction- This study focuses on the economic cost of mental disorders in young people (line 52). Therefore, it should be better introduced why, in addition to (mental) health services, also education, criminal justice and social care services were investigated.*

**Response:** We have edited the introduction as follows:

The high prevalence and potentially enduring nature of these impacts make addressing youth mental health conditions particularly important, but this is a challenge for public systems with limited resources (Knapp M; Evans-Lacko S, 2015). Economic costs associated with youth mental health conditions involve a wide range of sectors including health, educational, social care, and criminal justice services [9,10]. This can represent a substantial cost to the public purse, yet it could also be considered a wise investment given the evidence that effective treatment can mitigate the impact of poor mental health (Knapp et al., 2011).

**Comment 3:** *Line 55: male gender is mostly not associated with higher use of mental health services. Please specify the association between these factors and specific services.*

**Response:** We appreciate your suggestion. We have edited this paragraph in the revised version of the manuscript:

Some studies from high–income countries suggest that lower socioeconomic status, as well as clinical features (illness severity and impact of disorders) are associated with use of health, special education, and social care services, while male gender and older age are associated with more criminal justice services contacts [11,13,14]. These sociodemographic and clinical characteristics are also associated with greater mental health-related treatment costs among young people [11,14,15]

**Comment 4:** *Methods: Data and participants. I understand that not all information about the Brazilian High-Risk Cohort was included in this paper. I would want to know, however, based on what information the children became part of this high risk cohort. Are they COPMI?*

**Response:** Thanks for the important point you raised. We have added information in the methods on the Brazilian High-Risk cohort sampling procedures as follows:

This study is nested within the Brazilian High-Risk Cohort (BHRC), which is an ongoing prospective longitudinal study that comprises a community sample and a high‐risk sub‐sample (a sample at increased risk of mental disorders) of young people from Sao Paulo and Porto Alegre, Brazil. A detailed description of the sample and procedures can be found elsewhere [25]. Briefly, during the registry day, 12,500 parents of young people aged 6 to 14 years attending 57 schools (22 in Porto Alegre and 35 in São Paulo) were invited to a screening of mental health disorders using the Family History Screen (FHS) [26]. A total of 8,012 families (9,937 eligible children, 45,394 family members) were interviewed. Based on the percentage of members in the family that screened positively for psychiatric disorders, an index of family load for each potential eligible child was computed. The final cohort comprised 2,511 young people; 957 were randomly selected, and 1,554 were a sub‐sample at increased risk of mental disorders based on the FHS.

**Comment 5:** *Methods: Measures. Why only maternal educational level?
- Furthermore, this paragraph forms a clear description of appropriate measures.*

**Response:** As stated in the methods section, the socioeconomic group variable comprised head of household educational level in addition to other household socioeconomic indicators. As some research suggests that mothers educational level is particularly important for recognition and help-seeking, we also included this variable as a separate indicator. As the vast majority of caregiver respondents were mothers (in 93% of cases the biological mother [information included in the revised manuscript]) we focused on maternal education rather than estimating the educational level of other caregivers.

**Comment 6:** *Results. Very clear description and informative tables.*
**Response:** Thank you very much for your positive feedback.

**Comment 7:** *Discussion- Line 325: “We found that the health sector was clearly the main sector providing mental health care for youth.” That’s quite obvious. I would recommend rewriting this, for example: “**We found that the health sector was clearly the main sector accessed by youth with mental disorders.”*

**Response:** Thank you very much for your suggestion. We rewrote this sentence as follows:

We found that the health sector was clearly the main sector accessed by youth with mental disorders.

**Comment 8:** *In the present study, only 20% of young people with a diagnosed mental disorder received any form of care. In addition to reducing inequality in service use among children, these data also argue for lowering barriers to care for young people in general. I would recommend stating this in the conclusion as well.*

**Response:** Thanks for your suggestion. We have edited the first paragraph of the conclusions as follows:

Our findings suggest that the main drivers of health-related service use costs among adolescents in Brazil were impact of mental health problems, in addition to lower stigma toward people with mental illness among guardians and White ethnicity. In the present study, only 22.4% of young people with a diagnosed mental disorder received any form of care. In addition to reducing inequality in service use among children, our findings also argue for lowering barriers to care, in particular addressing caregiver stigma. Furthermore, because lower use of services in adolescence may be associated with worse outcomes across the life course [47], it is needed to further explore measures to reduce inequalities in service utilisation by young people, even though this implies higher short-term costs.

**Comment 9:** *Line 329: “The lack of youth-oriented primary care mental health programmes”. Is this also the reason why GP’s/family doctors were less frequently visited*?

**Response:** We appreciate your comment, and we agree with your interpretation of this result. We have edited the referred sentence:

The lack of youth-oriented primary care mental health programmes limits access to treatment when symptoms start to have an impact on adolescent functioning. This can explain why we found a low rate of mental health-related contacts with GP/ family doctors. As a result, contact with specialist mental health services only happens when the disorder has significant negative impact on the lives of young people.

**Comment 10:** *Line 359-361: this reads like the impact of mental health problems on children’s lives should be increased because it would support help-seeking. Please, rewrite.*

**Response:** We have rewritten this paragraph:

Guardian’s lower stigmatising attitudes towards mental disorders may be crucial to support young people in accessing, engaging and maintaining contact with mental health-related services. Various anti-stigma interventions have demonstrated effectiveness for improving help-seeking [49], but few have been implemented in LMICs. Further studies are needed to design and implement anti-stigma interventions in LMICs. On the other hand, health and education policies need to better support guardians to access appropriate and timely services in their communities, before the symptoms have a significant impact on adolescent functioning.

**Comment 11:** *Line 363: effectives should be effective*

**Response:** Thank you very much, we have corrected this error.

**Comment 12:** *In future research, it would be interesting to not only assess parental stigma but also stigma among the adolescents themselves.*

**Response:** We agree with you, and we are planning to evaluate the association between mental health-related service use and youth stigma towards mental illness in future cohort’s assessments.

**Reviewer #2:**

**General comment:** *It's good to see more representative research from LMICs, trying to bridge the existing knowledge gap. This study's most significant plus point is that it looks at service use and service cost from multiple angles, shedding light on demographic, clinical and systemic factors that contribute to service use cost. However, this manuscript does require significant improvement in language and content. Here are my main suggestions:*

**Response:** We appreciate your positive opinion of our work, the careful revision of our manuscript and your valuable comments.

**Comment 1:** *The language of the manuscript can be crisper. Multiple places sentences look disjointed or elongated. The paragraphs are changed too frequently in some places, with each of these paragraphs containing only one or two sentences.*
**Response:** Thanks for your comment. We have revised and edited the language through the manuscript.

**Comment 2:** *Introduction: In line 57, please clarify whether by 'education services' authors mean remedial education services or some other kind of services?*
**Response:** Thanks for your comment. We have indicated ‘special education’ in the revised version of the manuscript.

**Comment 3:** *Introduction: The lines 55-58 are difficult to follow: authors claim that certain demographic and clinical characteristics are associated with a greater likelihood of using certain services as per existing research. However, it's not clear how this connects with the assertion about young people in the same sentence.*
**Response:** Thanks for your comment. We have edited and separated these sentences:

Some studies from high–income countries suggest that lower socioeconomic status, as well as clinical features (illness severity and impact of disorders) are associated with use of health, special education, and social care services, while male gender and older age are associated with more criminal justice service contacts [11,13,14]. These sociodemographic and clinical characteristics are also associated with greater mental health-related treatment costs among young people [11,14,15].

**Comment 4:** *Introduction: The importance of studying parental stigma needs to be built better.*

**Response:** We appreciate your suggestion. We have included the following changes:
Families also play a central role in young people’s contact with services. One study from the UK found that lower mental illness-related stigma among caregivers was associated with an increased likelihood of young people’s mental health service use [16]. Stigmatising attitudes toward mental illness amongst parents may influence service contacts due to shame and fears of labelling their child’s mental health condition [16]. There are clear links between stigma and reduced help-seeking [17], reduced adherence to treatment and early withdrawal from services [17,18]. However, little is known about/ how parental stigma could impact on young people service use and costs.

**Comment 5:** *Introduction: I'm not sure what is meant by 'beyond diagnosis', are authors implying the existing studies cover the cost of diagnosis only or for limited kinds of disorders. Some clarification here would be helpful.*
**Response:** Thanks for your suggestion. We have edited this sentence as follows:

Additionally, little is known about how, in addition to the type of disorder, whether persistence of psychopathology from childhood to adolescence, disorders’ impact on adolescent’s daily life (i.e., functioning), and key barriers to care such as stigma, could influence costs.

**Comment 6:** *Introduction: The way lines 72-73 are written makes it sound like Brazil is a high-income country*
**Response:** We appreciate your comment. We have deleted ‘Similar to most high income countries’ in the revised version of the manuscript.

**Comment 7:** *Introduction: In line 88, it's unclear what characteristics the authors are referring to and whether the following hypothesis is related to a subset of these characteristics?*
**Response:** We have rewritten this sentence to clarify the characteristics under study:
Second, we examine how costs vary according to: mental health trajectories, impact of the disorder on everyday life, and parent/guardian stigma towards mental illness.

**Comment 8:** *Methods: In line 96, some information on how these children were classified as high risk will be helpful. The authors have said the details are somewhere else, but a brief description here will make it easier for the reader to understand the sample.*
**Response:** Thanks for your suggestion. As explained in response to Reviewer 1’s comment 4, we have included a brief description of the Brazilian High-Risk Cohort sampling procedures.

**Comment 9:** *Methods: In line 99, it was slightly hard to follow study timelines. Was this study carried out after the first follow-up in 2014-2015 or as part of the follow-up?*
**Response:** We have tried to clarify this including the following information:
Cohort participants were interviewed at baseline (aged 6-14 years, calendar year:2010-2011, n=2,511), and at first follow-up (N=2010, aged 9-17 years, calendar year 2014). After completing the BHRC first follow-up interview, 1,881 parents/guardians were invited to respond to a supplementary interview which included a comprehensive assessment of mental health related service use (calendar year: 2014-2015, young people participants aged 10-18 years).

**Comment 10:** *Methods: The authors can use consistent terminology: children or young people. As of now, this has varied from one sentence to another.*
**Response:** Thanks for your comment. We have revised and edited the methods section in order to use consistently the term young people.

**Comment 11:** *The '-' in line 102 seems typo.*
**Response:** We appreciate your comment. We have deleted this typo.

**Comment 12:** *Methods: In lines 127-130, it's unclear why young people were not interviewed at baseline but were included during the 3-year follow-up?*
**Response:** This was because participants were younger at baseline and so we relied on parent’s report, given limitations in funding and resources. Given that older adolescents are better at reporting internalising symptoms, both guardian and youth interviews were performed at 3-year follow-up. We included this explanation in the revised version of the manuscript:

At baseline, diagnostic assessment and interviews were performed with guardians only. Previous literature has found that self-reports on internalising conditions during adolescence is higher compared with parental report. This can be explained because internalising problems, such as anxiety or depression, would be less observable by guardians, being advisable to consider both reports to reach a reliable evaluation of adolescent mental health [30,31]. For this reason, diagnostic assessment at 3-year follow-up was performed considering guardian reports and additional information from interviews with the young people about internalising conditions.

**Comment 13:** *Methods: Do authors have any psychometric properties of the adapted version of Service Assessment for Children and Adolescents that can be reported in this publication?*
**Response:**

The parent-report SACA has been shown to be a valid measure of young people’s service use (kappa = 0.76; [Hoagwood et al., 2000]) with test-retest reliability for past-year reports (ranging from 0.75 to 0.86; [Horwitz et al., 2001]). We have not assessed the psychometric properties of the adapted version of the Service Assessment for Children and Adolescents for Brazilian participants yet. We have included this limitation in the revised version of the manuscript.

**Comment 14:** *Results: In line 224, the authors refer to Table 1. However, without any commentary on the significance of data in this table, the authors jump to a new set of findings. All this makes it slightly hard to follow what is being presented.*
**Response:** We appreciated your comment. We have edited this paragraph:
Table 1 describes sociodemographic and clinical characteristics of participants. The sample comprised 1,400 adolescents with a mean age of 14 years (s.d=1.98). The majority were white males from low SEG, and only 10% of mothers had university education. 23.3% (n= 326) of adolescents had a psychiatric disorder in the previous 12 months, of which 177 (54.3%) were incident and 149 (45.7%) persistent cases since baseline. 213 (15.2%) participants had remitted from a baseline psychiatric diagnosis. Participants with externalising disorders were more likely to have persistent trajectories (RR=2.19, 95%CI=1.38-3.48, p<0.001). Participants categorised as persistent also reported greater disorder impact (β=2.34, 95%CI=2.11-2.58, p<0.001). 22.4% of those who presented with a psychiatric disorder reported using some type of service for their mental health in the previous twelve months. The proportion of service use among those who presented a persistent psychiatric condition was 27%. Table 1 also describes the mean costs of mental health-related service use in the past year, by psychiatric trajectory (from no diagnosis to persistent psychiatric diagnosis). Bivariate analyses showed a non-significant association between psychiatric trajectory and mean annual costs.

**Comment 15:** *The 12-month service use and service use cost means are presented in Tables 1 and 2. Repeating the same findings across two tables should be avoided*
**Response:** We have deleted the last line of Table 2 (overall services cost).

**Comment 16:** *The paragraph on page 12 lacks a description of the cost associated with each service? For e.g., although CAPS is not a highly prevalent service, the associated cost makes for a lion contribution to the public purse. This needs to be presented and discussed.*
**Response:** Thank you very much for your suggestion. We edited this paragraph as follows:

Utilisation of mental health services in the previous 12 months and associated cost by type of service are presented in Table 2. Overall, 10.0% of the sample (n=143) used some sort of health, education, criminal justice or social care service for mental health problems. Disaggregating by sectors, the health sector had highest proportion of service users (9%), while the education and social care and criminal justice sectors were less frequently contacted with a 1.8% and 1.3% of users, respectively. Outpatient mental health services, most notably psychologists and psychiatrists in settings other than community mental health clinics, were the most frequently used services/professionals. Inpatient services and general health services such as GP/family doctor, paediatrician and emergency department, were less frequently used.

In the education sector, school assistant was the most type of service used by young people, while guardianship council was the most frequently social care service contacted. The total cost of 12-month mental health-related service use for the public purse was 70,110.23 USD. The sector that presented higher total annual cost was the health sector, followed by the education and finally the social care and criminal justice sectors. The services that generated the greatest total costs for the heath sector were psychologist (11,339.64 USD) and CAPS (9,628.01 USD). Among those who used services, the average annual cost of service use amounted to 527.14 USD (SD= 908.10 USD, range=8.77- 7,605.58 USD, median=221.10 USD, interquartile range=545.28) per user. Individuals using CAPS (specialty mental health) services (1.1% of the sample) had the highest mean number of visits during the previous year and the highest associated costs among health services. The second highest mean costs in the health sector were related to hospitalizations in psychiatric hospitals and alcohol and drugs clinics, while the lowest mean costs were attributed to emergency department, paediatrician, outpatient alcohol and drugs and GP/family doctor contacts. Although only 0.1% of individuals used shelters, this type of social service had the highest associated mean cost. Education services were used by 1.8% of individuals and these services had the second highest associated mean costs.

**Comment 17:** *Discussion: In line 288, the use of the terms 'above and beyond' doesn't convey much. To the best of my knowledge, the current analysis nowhere helps to reach this conclusion of above and beyond. I am requesting authors to look at terminology closely.*
**Response:** Thanks for your suggestion. We have removed this language.
We found that impact of mental health problems on daily life and parental stigma were the most consistent and robust drivers of mental health service use and associated costs..

**Comment** **18:** *Discussion: Lines 301-312 can be streamlined and better organised.*
**Response:** We have edited the cited lines as follows:

We did not find any study exploring the impact of parental stigmatising attitudes toward mental illness on child treatment costs. Other research has shown that parental stigma can impede problem recognition and help-seeking [17,43]. Higher stigma amongst parents and caregivers may discourage or delay service access for their children [16], which may reduce the short-term public sector direct costs of treatment but be detrimental in the long run. Future research needs to further explore the mechanisms through which parental stigma may be related to service/treatment selection and treatment adherence, in order to explain its impact on treatment costs. Moreover, as lower parental stigma may facilitate earlier service contact, it would be interesting to investigate if lower parental stigma may result in lower costs in the longer term.

**Comment 19:** *Discussion: Line 327: The number of CAPS users was less, but the number of visits and costs for those who used it were very high. These were not reflected in the discussion, nor were its implication for the restructuring health system.*
**Response:** We appreciate your comment. We have edited the discussion as suggested:

In Brazil, access to CAPS does not require any referral. However, the number of CAPS services are limited, and they are focused on treatment of severe mental disorders [24]. The high costs incurred by the mental health sector for the treatment of psychiatric disorders in CAPS may be a result of both, the severity of patients consulting these services and because these services provide intensive outpatient treatments (reflected by the highest number of visits we found), which is costly compared with no-specialized services. It is important to highlight that the lack of youth-oriented primary care mental health services in Brazil which limits access to treatment. This could explain why we found low frequency of mental health-related contacts with GP/ family doctors. As a result, contact with specialist mental health services only happens when the disorder has significant negative impact on the lives of young people. (Moved from the conclusion as suggested in your last comment). In this sense, the organisation of a mental health network of care for adolescents, integrating primary care, social care, education, criminal justice and community youth-specialist services, according to the impact of cases, must be considered in Brazil to adequately plan and allocate scarce public budgets [47].

**Comment 20:** *Discussion: The hypothesis stated that researchers were interested in examining the impact of persistence of psychiatric disorders from childhood to adolescence on service costs; however, the discussion did not give much attention to this part.*
**Response:** Thanks for rising this important comment. We have included the following paragraph:
Contrary to what we expected, we did not find an association between disorder persistence and costs. Our analyses instead found that impact of the disorder on adolescent’s life was the most important clinical predictor and that this was what seemed to drive service use rather than type or persistence of diagnosis. Nevertheless, it is important to consider that we have estimated annual costs, and these do not necessarily reflect the cumulative economic costs of persistent cases across childhood and adolescence.

**Comment 21:** *Conclusion: Some of the text in the last paragraph of the conclusion, i.e. those referring to implications, can be moved to discussion and expanded further.
I'm not able to comment on cost analysis as this is not my area of expertise.*

**Response:** Thanks for your suggestions, we have moved some conclusions to the discussion as explained in response to your Comment #20.
