# Supplementary material for: Utilisation and costs of mental health-related service use among adolescents
Source: PLoS One. 2022 Sep 9;17(9):e0273628. doi: 10.1371/journal.pone.0273628 (PMC9462733; doi:10.1371/journal.pone.0273628)
Supplement: Attachment — Submitted filename: Response to reviewers.docx [file pone.0273628.s014.docx]

**Responses to reviewer**

**Manuscript number PONE-D-21-10663R1**

**Manuscript Title: Utilisation and costs of mental health-related service use among adolescents**

We appreciate the careful revision of our manuscript and the comments of the reviewers. We are pleased to be invited to submit the revised version of our paper to PLOS ONE.

Please find attached both an unmarked version of the revised manuscript and one version with changes marked in red. Our point-by-point responses to the reviewers’ comments (unquoted italics) and details of the changes we have performed to our revised manuscript are given below.

***Reviewer’s general comment:*** *It's good to see the quality of the manuscript and the text organisation has been improved. I'm sharing a few follow-up queries. A sincere request to authors to include line number where revisions are reflected on the non-tracked version. Without line numbers, it’s difficult to locate revisions.*

We appreciate your careful revision of our manuscript and all your comments and suggestions. We have included the line number to locate the revisions of our manuscript.

***Follow-up query to comment 3:*** *In lines 57-59, the sentence's meaning is still not very clear. Are the mentioned demographic and clinical features associated with the frequent use of these services or the number of services used? Also, are these findings coming from adult literature, youth literature or across the age span?*

Thanks for your comment. We have edited this paragraph as follows:

Some studies from high–income countries about mental health-related service use among young people suggest that lower socioeconomic status, as well as clinical features (illness severity and impact of disorders) increase the likelihood of health, special education, and social care services, while male gender and older age are associated with more criminal justice services contacts [11,13,14]. (Introduction, lines 58-60)

***Follow-up query to comment 4****: In lines 65-69, the revised reasoning looks circular. The authors say that stigmatizing attitude among parents was associated with poor help-seeking. But in the following line states that little is known about the impact of parental stigma on help-seeking. Are authors implying that what is known about children is not applicable to adolescents? It's unclear how the existing findings relate to the gap that the author asserts in this para.*

Thanks for your comment. We have asserted this paragraph in order to explain that there is a clear link established between stigma influencing help-seeking among adults and adolescents (we have added one reference on the later topic), however, much less data on parental stigma in relation to their child’s service use has been published. We have added the only study that investigated the association between parental stigma and young people’s service use in the UK, published on January 27^th^, 2022. This paragraph was edited as follows:

Stigma can also influence help-seeking. Research in adult populations show clear links between stigma and reduced help-seeking [16], reduced adherence to treatment and early withdrawal from services [16,17]. Additionally, stigma is one of the most reported barriers to help-seeking amongst adolescents [18]. Families also play a central role in young people’s contact with services. However, there is limited research about how parental stigma could impact on service use and costs. Stigmatising attitudes toward mental illness amongst parents may influence service contacts due to shame and fear of labelling their youth’s mental health condition [19]. We know of two papers from the UK (using the same data), where lower mental illness-related stigma among caregivers (n=407), was associated with an increased likelihood of young people’s mental health service use [19], though it was not associated with costs [20]. We know of no studies on the association between mental illness-related stigma among caregivers and young people’s service use and costs outside the UK or in low and middle-income countries (LMICs), where families and young people may face different types of barriers to mental health care. (Introduction, lines 64-77)

New References were updated:

18. Aguirre Velasco A, Cruz ISS, Billings J, Jimenez M, Rowe S. What are the barriers, facilitators and interventions targeting help-seeking behaviours for common mental health problems in adolescents? A systematic review. BMC Psychiatry. 2020;20: 293. doi:10.1186/s12888-020-02659-0

20.	Ribeiro WS, Romeo R, King D, Owens S, Gronholm PC, Fisher HL, et al. Influence of stigma, sociodemographic and clinical characteristics on mental health-related service use and associated costs among young people in the United Kingdom. Eur Child Adolesc Psychiatry. 2022. doi:10.1007/s00787-022-01947-2

*The organization of the text in the introduction section can be improved as a couple of paragraphs have just one or two lines.*

Thanks for your suggestion. We have merged two paragraphs: lines 77 and 92.

***Methods*** *In line 121- is this '-' a typo?*

We have edited this sentence as follows:

Among those contacted, 1,400 (74.4%) guardians (in 93.1% of cases the biological mother) completed the interview, 982 (70.1%) by telephone and 418 (29.9%) face-to-face (See flow chart in S1 Fig.). (Line 126)

***In Line 116-121****, it is not clear to the reader how the study sample was reduced to 1881 from the original 2251. I'm guessing the authors included a sub-group of individuals meeting the age criteria. This needs to be clearly defined in the manuscript.*

We appreciate your comment. The Brazilian High-Risk Cohort (BHRC) comprises 2511 children, 2010 of them (80%) participated at the first follow-up. Due to an administrative error, we were only able to contact a subsample of those who participated at first follow-up (94%, n=1881) to participate in our additional research and thus we invited 1881 guardians to complete further questions on mental health-related service use and associated barriers to care. Of those participants who we contacted, 1,400 (74%) agreed to participate in our study.

We have included the following changes (in red) in lines 119-128:

Cohort participants were interviewed at baseline (aged 6-14 years, calendar year: 2010-2011, n=2,511), and at first follow-up (n=2010, aged 9-17 years, calendar year 2014, 80% retention rate). Due to an administrative error, we were only able to contact a subsample of those who participated at the first follow-up (94%, n=1,881) to respond to a supplementary interview which included a comprehensive assessment of mental health-related service use (calendar year: 2014-2015, young people participants aged 10-18 years). Among those contacted, 1,400 (74.4%) guardians (in 93.1% of cases the biological mother) completed the interview, 982 (70.1%) by telephone and 418 (29.9%) face-to-face (See flow chart in S1 Fig.). There were no significant differences in persistence of psychopathology or impact of psychopathology on adolescents’ lives among respondents versus non-respondents.

***Results
Follow-up query to comment 14:*** *It was helpful to see the description of the results in text. A few comments on the revised text: In line 253, the value in the text doesn't match with the value given in the table. If the rounding up was done, the number of decimal places should be consistent across the text. In line 262, there is no description of whether this service use is significantly different from other trajectories?*

We appreciate your comment. We have edited the value in line 259 (10.6%) and we have included the description of whether the service use was significantly different by trajectories:

Unadjusted odds ratios of any service use among participants with persistent diagnosis were 7.14 (95%CI=4.50-11.58, p<0.001) compared with participants with no diagnosis, OR=1.72 (95%CI=1.02-2.91, p=0.043) compared with incident and OR=2.62 (95%CI=1.52-4.49, p<0.001) with remittent diagnosis. (Lines 267-270).

***Follow-up query to comment 16:*** *Again, it was helpful to see the description of the results in text. A few comments on the revised text: In line 276, there should be a full stop before starting the following sentence. Also, it may be good to start as "within the health sector, the outpatient…", so the reader is clear that now you are looking at the frequency of usage within each sector.*

Thank you very much for your suggestion, we have corrected this as requested. (Line 284)

***In line 289****, it might be good to specify the mean visit per person, so it's clear to the reader that the mean refers to the mean visit per person.*

Thank you very much for your suggestion, we have corrected this as requested. (Line 297)

*The source of data in lines 334-337 is not provided.*

We appreciate your comment. Due to lines 334-337 are part of the first paragraph of the discussion, we wonder whether you are referring to lines 324-327. If so, we have edited this sentence as follows:
When looking at predictors of costs according to sector (Table 4), disorder impact was associated with greater health sector service use (predicted mean cost by each impact score= 66.26 USD, p=0.019). (Lines 343-345)

*There are a lot of supplementary data files, but except for two supplementary files, others were not referenced or explained in the results section. It would be helpful to understand the reason for including all this analysis but not using them in results.*

Supplementary analyses were explained in the methods section. Following your suggestion, we have included the description of supplementary tables in the results sections as follows:

Regarding to service use, lines 312-326:

Having an incident, remittent or persistent psychiatric disorder, as well as the higher impact of behavioural and emotional difficulties on the adolescents’ lives and lower parental stigma, all predicted higher odds of any 12-month service use in unadjusted analyses (S2 Table), in models adjusted by sociodemographic characteristics (S4-S6 Tables) and multivariable analyses (Table 3). Service contacts in the health sector were also predicted by the same factors. Service use in the educational sector was predicted by diagnosis trajectory, impact and lower stigma in the unadjusted analyses (S2 Table), and in the models adjusted by sociodemographic characteristics (S4-S6 Tables). However, disorder persistence did not remain significant in multivariable analyses, where impact, lower parental stigma and low SEG showed significant association with education service use (Table 3). Although persistence of the disorder and higher impact of behavioural and emotional difficulties on the adolescents’ lives were associated with social care and criminal justice service use in the unadjusted analyses (S2 Table), and in the models adjusted by sociodemographic characteristics (S4-S5 Table), there were no factors significantly associated with use of social care and criminal justice services in multivariable analyses (Table 3).

Additionally, to clarify that adjusted estimates are showed in Table 3, we have changed AOR (adjusted odds rations) instead OR (Line 328).

Regarding to services costs, lines 334-338:

When all three sectors were combined into a single total cost variable, greater impact and lower parental stigma were associated with higher costs in unadjusted analyses (S3 Table), in the models adjusted by sociodemographic characteristics (S8-S9 Tables) and multivariable analyses (Table 4). White ethnicity was also associated with higher costs in multivariable analyses (Table 4)….

Lines 345-350:

We did not find any significant association of psychiatric trajectories, impact of disorder or parental stigma with education sector’s costs (S3 Table, S7-S9 Tables, and Table 4). Although persistence of the disorder was associated with social care and criminal justice service’s costs in the unadjusted analyses (S3 Table), and in the models adjusted by sociodemographic characteristics (S7 Table), we did not find any significant factors associated with social care/criminal justice sectors’ costs in multivariable analyses (Table 4).

***Discussion*** *The findings discussed in lines 342-346 don't match the result. The service use was different among those with different trajectories. However, the costs were not different among these groups. Does any of the existing analysis carried out by the author can explain why the difference in frequency of use didn't result in differences in cost between these groups.*
Thanks for your comment. Apologise for the confusion. We have corrected that we were discussing in this part the results of service costs instead of service use, as follows:

Our analyses instead found that impact of the disorder on adolescent’s life was the most important clinical predictor and that this was what seemed to drive service costs rather than type or persistence of diagnosis. (Lines 366-368)

Regarding to your question about whether our analyses can explain why the difference in frequency of use did not result in differences in cost between these groups, service costs reflect the intensity of care (number of visits, as explained in the introduction) while service use reflect the probability of having any contact with the services. In this sense, our results suggest that the persistence of a diagnosis can increase the likelihood of service use but does not necessarily have an impact on the number of contacts per year. Unfortunately, as pointed out in the limitations of our study (lines 433-435), the scarce number of service users by specific services did not allow us to perform analyses to compare, for example, whether the trajectory it is associated to the selection of CAPS (high-costs services) instead primary care (low-costs services) services contacts. We have added the following sentence to the discussion:

This finding suggests that although the trajectory of the diagnosis is associated with a greater likelihood of having any contact with mental health-related services, this association did not translate to costs which also reflects the intensity and / or type of care and number of visits per year. Nevertheless, it is important to consider that we have estimated annual costs, and these do not necessarily reflect the cumulative economic costs of persistent cases across childhood and adolescence. (Lines 368-374).

***Follow-up query to comment 18****: The revision looks good. However, in line 347, the authors' assertion that these findings are novel seems misleading. As in quick succession, they have indicated that other studies have also found similar findings.*

Thanks for your comment. We have edited this sentence as follows:

We also found that lower parental stigma was associated with greater service use and higher costs. (Line 371)

Additionally, as we are aware of a new paper (published on January 27^th^, 2022) that investigate the association between parental stigma and service use costs, we have edited the paragraph in lines (381-384):

We only found one study [20] exploring the impact of parental stigmatising attitudes toward mental illness on child service use costs. The cited study, conducted in the UK, with a smaller sample size, did not find an association between parental stigma and young people’s service use costs.

***Follow-up query to comment 19****: The discussion points on CAPS results in lines 380-385 are difficult to follow. There is no mention in results about the severity of disorders and types of service accessed. So understanding this link in discussion is difficult.*

We appreciate your comment. We eliminated the sentence that connected severity of cases with CAPS’s costs and the sentence in the revised version appears as follows:

The high costs incurred by the mental health sector for the treatment of psychiatric disorders in CAPS may be because these services provide intensive outpatient treatments (reflected by the highest number of visits we found), which is costly compared with no-specialized services. (Lines 408-411)
