# Supplementary material for: Utilisation and costs of mental health-related service use among adolescents
Source: PLoS One. 2022 Sep 9;17(9):e0273628. doi: 10.1371/journal.pone.0273628 (PMC9462733; doi:10.1371/journal.pone.0273628)
Supplement: Attachment — Submitted filename: Response to Reviewers.docx [file pone.0273628.s015.docx]

**Responses to reviewer**

**Manuscript number PONE-D-21-10663R2**

**Manuscript Title: Utilisation and costs of mental health-related service use among adolescents**

We appreciate the careful revision of our manuscript and the comments of the reviewers. We are pleased to be invited to submit the revised version of our paper to PLOS ONE.

Please find attached both an unmarked version of the revised manuscript and one version with changes marked in red. Our point-by-point responses to the reviewers’ comments (unquoted italics) and details of the changes we have performed to our revised manuscript are given below.

*Reviewer #1: My comments were already addressed, to my satisfaction, in the previous version of the manuscript.*

Many thanks for your revision and we are glad of having addressed your comments appropriately.

*Reviewer #2: Thank you for addressing most of my comments. The manuscript is looking well. Here are a suggested few minor revisions:
In lines 59-61, it is still unclear whether these features increase the likelihood of service usage or the number of services used.*

Thanks for your suggestion. We have edited this paragraph as follows:

Lines 59-62: Some studies from high–income countries about mental health-related service use among young people suggest that lower socioeconomic status, as well as clinical features (illness severity and impact of disorders) increase the likelihood of service usage in the health, special education, and social care sectors, while male gender and older age are associated with more criminal justice services contacts [11,13,14].

*In lines 121-122, the authors have stated that 'due to an administrative error, we were only able to contact a subsample of those who participated at the first follow-up'. It will be good to have more details about this error in the study limitation and how this may have affected the validity of the results.*

We appreciate your comment. We have provided more details about this error in lines 121-123 (Methods section):

Due to an administrative error, the service use questions were not included in the interview schedule for the first 129 participants (6%) of the BHRC first follow-up. Therefore, we were only able to contact a subsample of those who participated at the first follow-up (94%, n=1,881) to respond to a supplementary interview which included a comprehensive assessment of mental health-related service use (calendar year: 2014-2015, young people participants aged 10-18 years).

And we added in the Limitations subheading the following sentences:

Lines 440-442: Fifth, due to an administrative error we were unable to contact the first 6% of first follow-up BHRC participants. This could reduce our sample size, but as this was a random error, we do not believe that it affected the results.

*For lines 261-272, I'm really struggling to figure out where all these percentage values, OR, and RR are coming from, as they are not given in Table 1. If it's from some supplementary table, please cite it.*

We appreciate the comment of the reviewer.

To clarify the percentage of service utilization we edited the text as follows:

Lines 261-267: 23.3% (n= 260) of adolescents had a psychiatric disorder in the previous 12 months, of which (54.3%) 261 were incident and 149 (45.7%) persistent cases since baseline. 213 (15.2%) participants had remitted from a baseline psychiatric diagnosis. **[We moved the next sentence here]** 73 (22.4%) of those who presented with a psychiatric disorder (32 incident and 41 persistent) reported using some type of service for their mental health in the previous twelve months. The proportion of service use among those who presented a persistent psychiatric condition was 27.5%.

Regarding to the following text added in our second revision:

Lines 267-270: Unadjusted odds ratios of any service use among participants with persistent diagnosis were 7.22 **[we amended these decimals]** (95%CI=4.50-11.58, p<0.001) compared with participants with no diagnosis, OR=1.72 (95%CI=1.02-2.91, p=0.043) compared with incident and OR=2.62 (95%CI=1.52-4.49, p<0.001) with remittent diagnosis.

These results were added with the aim of addressing your comment on whether there were differences in service use between trajectories. In Supplementary Table 2 we presented these differences taking as reference ‘no diagnosis’, and the results presented in this text (Lines 267-270) would be taking as reference ‘persistent psychiatric conditions’. These results were not presented in a specific table.

Regarding to other statistics informed in the lines cited by the reviewer, Table 1 describes sociodemographic and clinical characteristics of participants, according to mental health conditions trajectories. We added only in the text (i.e. not in a specific table) the results of bivariate statistics for disorder impact (one of our main predictors) and type of broad diagnostic group by trajectories. To clarify this, we decided to only maintain the mean differences in disorder impact by trajectories and we removed the differences in terms of type of diagnostic group, as this was not the focus of our study. Changes were added as follows:

Lines 271-275: Table 1 present mean disorder impact by psychiatric trajectories. Unadjusted generalised regression models showed that persistent cases presented greater mean difference in disorder impact (SDQ scores) by 2.34, (95%CI=2.11-2.58, p<0.001) compared with no diagnosis, 1.14 (95% CI=0.85-1.42, p<0.001), compared with incident cases, and 1.84 (95% CI=1.56-2.12, p<0.001), compared with remittent cases.

*In lines 258 and 267, the value in the text doesn't match the value given in the table. If the rounding up was done, the number of decimal places should be consistent across the text*

Thanks for your suggestion, we amended these values as follows:

Line 260

14.51 years (s.d=1.98).

Line 267

27.5%.

And we also corrected the decimal places in the percentage of overall service use in Line 285 and Table 1

10.2%
